# Novel Camptothecin-Based Lipid Conjugated with Steroids and Triterpene Acids: Enhanced Cellular Uptake and Topoismerase I Inhibition

**DOI:** 10.3390/pharmaceutics18070867

**Published:** 2026-07-16

**Authors:** Nuri M. Chobanov, Konstantin O. Vakhnin, Stepan E. Logunov, Alexander E. Fedorov, Georgii V. D’yakonov, Usein M. Dzhemilev, Lilya U. Dzhemileva, Vladimir A. D’yakonov

**Affiliations:** 1 N.D. Zelinsky Institute of Organic Chemistry, Russian Academy of Sciences, 47 Leninsky Prosp., Moscow 119991, Russia; 2 Institute of Physiologically Active Compounds at Federal Research Center of Problems of Chemical Physics and Medicinal Chemistry, Russian Academy of Sciences (IPAC RAS), Severniy Proezd 1, Chernogolovka 142432, Russia

**Keywords:** camptothecin, camptothecin–lipid conjugates, steroidal carriers, triterpenoids, topoisomerase I inhibitors, cell cycle arrest, apoptosis, ADME profiling

## Abstract

**Background/Objectives**: The clinical application of the potent antitumor alkaloid camptothecin is severely limited by its high systemic toxicity, poor solubility, and rapid inactivation. This study aimed to design, synthesize, and evaluate a novel series of camptothecin–lipid conjugates where natural steroids and triterpenoids serve as lipophilic carriers to enhance cellular penetration and mitigate cardiotoxicity. Herein, we implemented an endogenous targeting strategy by conjugating camptothecin with natural steroids and triterpenoids, which serve as biomimetic lipophilic vectors to achieve tumor-selective intracellular delivery. **Methods**: Eleven novel camptothecin-based hybrids linked via succinic acid or ethylene glycol spacers were synthesized and characterized. Their in vitro cytotoxicity was evaluated against eight cancer cell lines of varied embryologic origin (Jurkat, HCT 116, A549, HL60, K562, HeLa, HEK293, and Fibroblasts). Cell cycle distribution, apoptosis induction, and topoisomerase I inhibitory activity were assessed using flow cytometry and enzyme assays. In silico pharmacokinetic profiling, including P-glycoprotein interaction and cardiotoxicity mitigation, was substantiated via ADME algorithms. **Results**: Among the series, compounds **12** (cholestanol conjugate) and **26** (betulin derivative) emerged as the primary lead candidates based on an optimal composite of submicromolar cytotoxicity, exceptional tumor selectivity indices (up to 7.0 for **12**), and active topoisomerase I inhibition. Compounds **11**–**14** generally shared prominent S-phase cell cycle arrest, while the lead hybrids **12** and **26** successfully combined high apoptotic triggering with favorable in silico ADME safety profiling, including eliminated cardiotoxicity for the structural analogs. **Conclusions**: The integration of steroidal and triterpenoid pharmacophores via strategic spacers provides a reliable foundation for developing targeted, low-toxicity camptothecin–lipid conjugates with optimized safety profiles for cancer therapy.

## 1. Introduction

**Figure 1:**
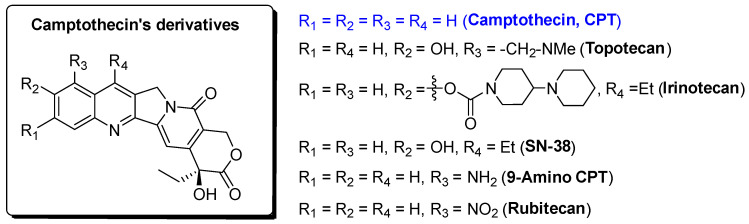
The topoisomerase I inhibitor camptothecin (CPT) and some of its derivatives.

Camptothecin (CPT) is an alkaloid that is produced by certain species of coated seeds from diverse families, including *Camptotheca acuminate Decne* (*Nyssaceae*), *Nothapodytes foetida*, *Pyrenacantha klaineana*, *Merrilliodendron megacarpum* (*Icacinaceae*), *Ophiorrhiza pumila* (*Rubiaceae*), *Ervatamia heyneana* (*Apocynaceae*), and *Mostuea brunonis* (*Gelsemiaceae*) [1]. CPT is a molecule with a pronounced anticancer effect based on irreversible inhibition of topoisomerase I. In 1958, the compound was derived from *Camptotheca acuminata*, a deciduous tree that is native to China and Tibet and is widely used in traditional Chinese medicine. The tree is also referred to as “Si Shu” or “tree of joy.” It is evident that CPT and its synthetic derivatives possess a distinctive pentacyclic structure (Figure 1) [2]. CPT has gained renown for its antitumor activity, which has been demonstrated in a wide range of human cancers [3,4,5,6,7]. Moreover, a plethora of studies have documented the multifaceted pharmacological properties of this compound, including its pesticidal, antipsoriatic, antiparasitic, antifungal, antimicrobial, and antiviral activities [8].

**Figure 2:**
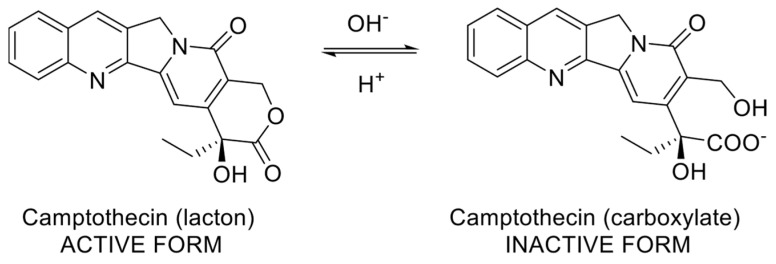
The equilibrium between the active lactone form of camptothecin and its inactive carboxylate derivative.

However, CPT exhibits certain drawbacks that compromise its anticipated efficacy, including pH dependence and inadequate aqueous solubility. At physiological pH, camptothecin undergoes hydrolysis, resulting in partial conversion to the carboxylate form. This process involves the opening of the lactone ring, leading to the molecule’s biological inactivity (Figure 2) [9].

Advancements in the medicinal chemistry of CPT in the late 1980s and early 1990s resulted in the availability of semi-synthetic, water-soluble analogs, irinotecan and topotecan. These are used in clinical practice for the treatment of solid tumors of the gastrointestinal tract and respiratory system [10,11,12,13]. Camptothecin-based peptide–drug conjugates (PDCs) targeting integrin αvβ3 represent a promising strategy for the treatment of pancreatic and lung cancers. The authors designed and synthesized a series of integrin αvβ3-selective PDCs using a 1,3,5-triazine-based linker to conjugate a camptothecin derivative with the c(RGDfC) peptide [5]. A notable drawback of camptothecin derivatives is the rapid development of resistance, which necessitates the continuous pursuit of novel soluble camptothecin analogs that exhibit both high efficacy and reduced toxicity [14,15,16]. In addition, numerous research groups are engaged in the continuous pursuit of novel topoisomerase inhibitors. These inhibitors, in addition to their efficacy, possess enhanced penetration ability and more effective and faster action on tumor cells [3,17,18]. A prodrug of irinotecan, 7-ethyl-10-hydroxycamptothecin (SN38), has demonstrated high antitumor activity. It is this active metabolite that is predominantly responsible for the antitumor effect [19]. According to experimental data, SN38 is approximately 100 times more active than irinotecan itself, and its effect varies greatly depending on the histological type of tumor [20]. Enhancing the therapeutic potential of camptothecin is also achieved by attaching this pharmacophore to monoclonal antibodies that are tropic to solid tumor proteins. This strategy has proven highly successful for a novel methylenedioxy-camptothecin analog [21]. Unlike macromolecular ADCs or peptide-drug conjugates (PDCs) that rely on specific receptor-mediated protein endocytosis, the small-molecule lipophilic hybrids designed in this work utilize biomimetic lipid vectors. This approach targets overexpressed lipid rafts directly through membrane microdomain partitioning, offering a distinct, non-macromolecular strategy for tumor-selective delivery.

In light of the aforementioned studies, this research endeavored to integrate a series of steroidal (cholesterol, cholestanol, lithocholic acid) and triterpenoid (betulinic acid, oleanolic acid, ursolic acid, and 18β-glycyrretinic acid) pharmacophores into the camptothecin molecule at disparate locations. The objective was to facilitate the assembly of the two preferred pharmacophores into a unified molecular structure. The objective of this hybridization process is to enhance the anticancer activity of camptothecin while simultaneously improving its physicochemical properties. The deployment of these natural carrier molecules exploits the metabolic reliance of tumor cells on exogenous lipids, as the high density of lipoprotein receptors and lipid rafts on malignant membranes provides a robust rationale for receptor-mediated targeted delivery and accelerated intracellular uptake of the engineered hybrids. The pharmacophores presented in this study have not been previously studied or reported in the literature. Our research group has previously presented a number of papers on topoisomerase inhibitors I and II, as well as detailed studies on the biological properties of many reversible topoisomerase inhibitors [22,23]. This study is pioneering the development of such hybrids to assess the potential synergism in anticancer activity. The designed compounds are expected to possess distinct anticancer activity associated with both fused scaffolds, resulting in enhanced efficacy and unique physicochemical properties. These hybrids are potential candidates for the development of effective and low-toxicity next-generation anticancer drugs.

## 2. Materials and Methods

### 2.1. General

All commercial reagents were purchased from Sigma-Aldrich (St. Louis, MO, USA) and Acros Organics (Geel, Belgium). All commercially available solvents and reagents used were of analytical grade and without further purification. Reactions were monitored by TLC on Sorbfil plates (IMID Ltd., Krasnodar, Russia). Column chromatography was carried out on Acrus silica gel (Geel, Belgium) (0.060–0.200 mm). Purity of all final compounds was 97% or higher. Melting points were recorded on Stuart SMP3 (Bibby Scientific, Stone, UK). IR spectra were recorded on Bruker VERTEX 70V spectrometer (Bruker Optik GmbH, Ettlingen, Germany) using KBr discs over the range of 400–4000 cm^−1^. ^1^H and ^13^C NMR spectra were obtained using a Bruker AVANCE 300 spectrometer (Bruker BioSpin GmbH, Rheinstetten, Germany) in CDCl_3_ operating at 300 MHz for ^1^H and 75 MHz for ^13^C. High-resolution mass spectra with electrospray ionization were measured with a BrukerMicroOTOF II instrument (Bruker Daltonik GmbH, Bremen, Germany); external calibration of the mass spectrometer was performed using the Electrospray Tuning mix (Agilent Technologies, Santa Clara, CA, USA).

### 2.2. Synthesis and Purification

General procedure for the synthesis of conjugates (**6**–**10**). Derivatives (**1**, **2**, **4**–**6**), respectively (0.24 mmol) were dissolved in 5 mL of DMF, then EDC*HCl (37.3 mg, 0.24 mmol) and DMAP (29.3 mg, 0.24 mmol) were successively added to the resulting solution at 0 °C and stirred for 15 min. at 0 °C. Then the reaction was brought to room temperature and CPT (41.80 mg, 0.12 mmol) was added and stirred at r.t. under (Ar) atmosphere for 48 h. Upon completion, the resulting reaction mass was filtered and concentrated. The resulting crude product was purified by column chromatography on SiO_2_ (EtOAc) to obtain hybrid molecules (**8**, **9**, **11**–**13**).

(S)-4-ethyl-3,14-dioxo-3,4,12,14-tetrahydro-1H-pyrano[3′,4′:6,7]indolizino[1,2-b]quinolin-4-yl (methyl 3β-O-carboxypropionyl)urs-12-en-28-oate (**8**). Light yellow solid (168.1 mg, 76% yield). ^1^H NMR (300 MHz, Chloroform-*d*) δ 8.43 (s, 1H), 8.30 (d, *J* = 8.6 Hz, 1H), 7.95 (d, *J* = 8.1 Hz, 1H), 7.85 (t, *J* = 7.7 Hz, 1H), 7.67 (t, *J* = 7.6 Hz, 1H), 7.39 (s, 1H), 5.67 (d, *J* = 17.2 Hz, 1H), 5.39 (d, *J* = 17.3 Hz, 1H), 5.29 (s, 2H), 5.23 (d, *J* = 3.7 Hz, 1H), 4.38 (dd, *J* = 10.3, 5.7 Hz, 1H), 3.60 (s, 3H), 2.95–2.77 (m, 2H), 2.70–2.56 (m, 2H), 2.35–2.08 (m, 3H), 2.06–1.88 (m, 1H), 1.88–1.13 (m, 18H), 1.07–0.92 (m, 10H), 0.88 (d, *J* = 6.4 Hz, 3H), 0.80 (d, *J* = 7.1 Hz, 6H), 0.70 (d, *J* = 10.2 Hz, 6H), 0.62 (d, *J* = 10.5 Hz, 1H). ^13^C NMR (75 MHz, CDCl_3_) δ 178.06, 171.56, 171.40, 167.38, 157.32, 151.99, 148.22, 145.97, 145.62, 138.15, 131.71, 130.97, 129.22, 128.59, 128.23, 128.18, 125.45, 120.45, 97.10, 81.51, 76.13, 67.01, 55.24, 52.87, 51.45, 49.97, 48.08, 47.38, 41.95, 39.43, 39.06, 38.88, 38.14, 37.63, 36.72, 36.64, 32.81, 31.76, 30.65, 29.22, 29.02, 28.05, 28.00, 24.21, 23.64, 23.40, 23.24, 21.20, 18.10, 17.07, 16.83, 16.75, 15.33, 7.62. ESI-MS: *m*/*z* Calcd for C_55_H_68_N_2_O_9_ [M + Na]^+^: 923.4817; Found: 923.4827.

(S)-4-ethyl-3,14-dioxo-3,4,12,14-tetrahydro-1H-pyrano[3′,4′:6,7]indolizino[1,2-b]quinolin-4-yl (methyl 3β-O-carboxypropionyl)olean-12-en-28-oate (**9**). Light yellow solid (175.5 mg, 78% yield). ^1^H NMR (300 MHz, Chloroform-*d*) δ 8.40 (s, 1H), 8.26 (d, *J* = 8.5 Hz, 1H), 7.94 (d, *J* = 8.1 Hz, 1H), 7.84 (t, *J* = 7.7 Hz, 1H), 7.67 (t, *J* = 7.5 Hz, 1H), 7.30 (s, 1H), 5.67 (d, *J* = 17.3 Hz, 1H), 5.41 (d, *J* = 17.2 Hz, 1H), 5.28 (s, 2H), 4.42 (t, *J* = 8.0 Hz, 1H), 3.63 (s, 3H), 2.92–2.80 (m, 3H), 2.64 (q, *J* = 6.3 Hz, 2H), 2.30 (dd, *J* = 14.0, 7.3 Hz, 1H), 2.23–2.10 (m, 1H), 2.05–1.89 (m, 1H), 1.79 (d, *J* = 10.1 Hz, 2H), 1.71–0.53 (m, 44H). ^13^C NMR (75 MHz, CDCl_3_) δ 178.29, 171.49, 171.38, 167.43, 157.37, 152.31, 148.76, 146.10, 145.83, 143.81, 131.27, 130.74, 129.63, 128.51, 128.22, 128.18, 128.09, 122.27, 120.36, 96.48, 81.46, 76.16, 67.10, 55.27, 51.55, 49.96, 47.48, 46.74, 45.93, 41.63, 41.32, 39.24, 38.00, 37.68, 36.82, 33.89, 33.15, 32.54, 32.40, 31.85, 30.73, 29.27, 29.07, 28.03, 27.70, 25.97, 23.68, 23.42, 23.37, 23.09, 18.14, 16.79, 16.73, 15.22, 7.63. ESI-MS: *m*/*z* Calcd for C_55_H_68_N_2_O_9_ [M + Na]^+^: 923.4817; Found: 923.4817.

(S)-4-ethyl-3,14-dioxo-3,4,12,14-tetrahydro-1H-pyrano[3′,4′:6,7]indolizino[1,2-b]quinolin-4-yl (methyl 3β-O-carboxypropionyl)-11-oxoolean-12-en-30-oate (**11**). Yellow solid (175.8 mg, 80% yield). ^1^H NMR (300 MHz, Chloroform-*d*) δ 8.44 (s, 1H), 8.28 (d, *J* = 8.5 Hz, 1H), 7.95 (d, *J* = 8.1 Hz, 1H), 7.85 (t, *J* = 7.7 Hz, 1H), 7.67 (t, *J* = 7.6 Hz, 1H), 7.35 (s, 1H), 5.66 (d, *J* = 2.8 Hz, 2H), 5.44 (s, 1H), 5.30 (s, 2H), 4.47 (dd, *J* = 10.8, 5.5 Hz, 1H), 3.71 (s, 3H), 2.96–2.80 (m, 3H), 2.75–2.55 (m, 3H), 2.40–2.16 (m, 3H), 2.16–2.04 (m, 1H), 2.04–1.95 (m, 3H), 1.95–1.73 (m, 1H), 1.64 (d, *J* = 13.5 Hz, 2H), 1.60–1.54 (m, 6H), 1.51–1.28 (m, 8H), 1.17 (s, 3H), 1.10–1.03 (m, 3H), 1.02 (s, 1H), 0.98 (d, *J* = 7.6 Hz, 5H), 0.86–0.74 (m, 8H), 0.66 (d, *J* = 11.1 Hz, 1H). ^13^C NMR (75 MHz, CDCl_3_) δ 200.04, 176.98, 171.47, 171.38, 169.33, 167.45, 157.32, 152.17, 148.52, 145.96, 145.75, 131.56, 130.84, 129.45, 128.56, 128.45, 128.23, 128.19, 128.10, 120.43, 96.61, 81.11, 76.12, 67.10, 61.61, 54.94, 51.84, 49.99, 48.39, 45.34, 44.06, 43.15, 41.10, 38.69, 38.03, 37.75, 36.79, 32.60, 31.84, 31.13, 29.27, 29.02, 28.54, 28.37, 28.01, 26.44, 26.39, 23.40, 18.60, 17.27, 16.73, 16.23, 7.64. ESI-MS: *m*/*z* Calcd for C_55_H_66_N_2_O_10_ [M + Na]^+^: 937.4610; Found: 937.4603.

(S)-4-ethyl-3,14-dioxo-3,4,12,14-tetrahydro-1H-pyrano[3′,4′:6,7]indolizino[1,2-b]quinolin-4-yl-cholestan-3β-hemisuccinate (**12**). White solid (180.9 mg, 92% yield). ^1^H NMR (300 MHz, Chloroform-*d*) δ 8.42 (s, 1H), 8.30 (d, *J* = 8.4 Hz, 1H), 7.96 (d, *J* = 8.0 Hz, 1H), 7.85 (t, *J* = 7.8 Hz, 1H), 7.69 (t, *J* = 7.7 Hz, 1H), 7.40 (s, 1H), 5.69 (d, *J* = 17.2 Hz, 1H), 5.40 (d, *J* = 17.3 Hz, 1H), 5.28 (s, 2H), 4.61 (s, 1H), 0.64 (d, *J* = 18.4 Hz, 6H). ^13^C NMR (75 MHz, CDCl_3_) δ 171.56, 171.37, 167.45, 157.40, 152.25, 148.60, 146.14, 145.83, 131.45, 130.87, 129.60, 128.59, 128.28, 128.22, 128.16, 120.22, 97.03, 76.22, 74.26, 67.01, 56.46, 56.35, 54.01, 49.98, 44.36, 42.59, 40.02, 39.55, 36.57, 36.21, 35.82, 35.32, 35.25, 33.80, 31.77, 29.26, 29.01, 28.42, 28.26, 28.05, 27.32, 24.20, 23.90, 22.86, 22.60, 21.11, 18.72, 12.05, 7.67. MS (ESI): *m*/*z* Calcd for C_51_H_66_N_2_O_7_ [M + H] ^+^: 819.4942; Found: 819.4943.

(S)-4-ethyl-3,14-dioxo-3,4,12,14-tetrahydro-1H-pyrano[3′,4′:6,7]indolizino[1,2-b]quinolin-4-yl-cholesteryl hemisuccinate (**13**). White solid (174.4 mg, 89% yield). ^1^H NMR (300 MHz, Chloroform-*d*) δ 8.38 (s, 1H), 8.24 (d, *J* = 8.6 Hz, 1H), 7.93 (d, *J* = 8.2 Hz, 1H), 7.82 (t, *J* = 7.7 Hz, 1H), 7.66 (t, *J* = 7.5 Hz, 1H), 7.32 (s, 1H), 5.68 (d, *J* = 17.3 Hz, 1H), 5.39 (d, *J* = 17.1 Hz, 1H), 5.27 (s, 2H), 5.04 (d, *J* = 4.9 Hz, 1H), 4.52 (dd, *J* = 11.2, 5.9 Hz, 1H), 2.93–2.73 (m, 2H), 2.61 (q, *J* = 6.2 Hz, 2H), 2.28 (dd, *J* = 14.3, 7.4 Hz, 1H), 2.14 (dt, *J* = 14.8, 7.5 Hz, 3H), 1.97 (d, *J* = 12.4 Hz, 1H), 1.89–1.72 (m, 3H), 1.65 (d, *J* = 13.3 Hz, 1H), 1.58–1.45 (m, 3H), 1.30 (dq, *J* = 16.0, 8.3, 7.3 Hz, 9H), 1.19–1.04 (m, 8H), 0.99 (t, *J* = 7.6 Hz, 4H), 0.88–0.84 (m, 12H), 0.63 (s, 3H). ^13^C NMR (75 MHz, CDCl_3_) δ 171.48, 171.23, 167.48, 157.43, 152.38, 148.86, 146.11, 146.02, 139.52, 131.22, 130.73, 129.80, 128.55, 128.22, 128.07, 122.36, 120.20, 96.65, 76.22, 74.50, 67.10, 56.73, 56.22, 49.99, 49.84, 42.33, 39.77, 39.57, 37.90, 36.81, 36.44, 36.24, 35.84, 31.85, 31.80, 31.76, 29.24, 29.03, 28.27, 28.07, 27.65, 24.31, 23.90, 22.89, 22.63, 20.96, 19.19, 18.78, 11.86, 7.67. MS (ESI): *m*/*z* Calcd for C_51_H_64_N_2_O_7_ [M + Na]^+^: 839.4606; Found: 839.4623.

Procedure for the synthesis of hybrid molecule **10**. Acid **3** (80 mg, 0.14 mmol) was dissolved in 5 mL of DMF, then EDC*HCl (21.73 mg, 0.14 mmol) and DMAP (17.1 mg, 0.14 mmol) were successively added to resulting solution at 0 °C and stirred for 20 min. at 0 °C. Then the reaction was brought to rt. and CPT (camptothecin) (24.40 mg, 0.07 mmol) was added and stirred at r.t. under an Ar atmosphere for 48 h. Upon completion, the resulting reaction mass was filtered and concentrated. The resulting crude product was purified by column chromatography on SiO_2_ (EtOAc) to obtain conjugate **10**.

(S)-4-ethyl-3,14-dioxo-3,4,12,14-tetrahydro-1H-pyrano[3′,4′:6,7]indolizino[1,2-b]quinolin-4-yl (methyl (3β-O-carboxypropionyl))lup20(29)-en-28-oate (**10**). Yellow solid (93.6 mg, 74% yield). ^1^H NMR (300 MHz, Chloroform-*d*) δ 8.40 (s, 1H), 8.27 (d, *J* = 8.5 Hz, 1H), 7.95 (d, *J* = 8.2 Hz, 1H), 7.85 (t, *J* = 7.7 Hz, 1H), 7.67 (t, *J* = 7.5 Hz, 1H), 7.32 (s, 1H), 5.68 (d, *J* = 17.3 Hz, 1H), 5.41 (d, *J* = 17.2 Hz, 1H), 5.29 (s, 2H), 4.76 (s, 1H), 4.63 (s, 1H), 4.38 (dd, *J* = 10.6, 5.2 Hz, 1H), 3.67 (s, 3H), 3.07–2.93 (m, 1H), 2.93–2.79 (m, 2H), 2.70–2.58 (m, 3H), 2.30 (dd, *J* = 14.5, 7.7 Hz, 1H), 2.17 (dd, *J* = 17.2, 10.3 Hz, 3H), 1.90 (d, *J* = 11.6 Hz, 2H), 1.69–1.47 (m, 4H), 1.45–1.34 (m, 6H), 1.34–1.29 (m, 3H), 1.27 (s, 4H), 1.13 (s, 3H), 1.05–0.82 (m, 10H), 0.77 (s, 3H), 0.70 (d, *J* = 5.1 Hz, 6H), 0.57 (d, *J* = 8.7 Hz, 1H). ^13^C NMR (75 MHz, CDCl_3_) δ 176.68, 171.51, 171.41, 167.45, 157.41, 152.40, 150.63, 148.85, 146.12, 145.90, 131.22, 130.73, 129.74, 128.55, 128.22, 128.09, 120.38, 109.65, 96.58, 81.52, 76.21, 67.12, 56.61, 55.45, 51.30, 50.44, 49.98, 49.56, 47.04, 42.43, 40.70, 38.34, 38.30, 37.81, 37.02, 34.25, 32.23, 31.88, 30.69, 29.72, 29.32, 29.12, 27.95, 25.56, 23.61, 20.90, 19.48, 18.14, 16.56, 16.05, 15.94, 14.85, 7.66. MS (ESI): *m*/*z* Calcd for C_55_H_68_N_2_O_9_ [M + Na] ^+^: 923.4817; Found: 923.4817.

Procedure for the synthesis of hybrid molecule **14**. Steroid derivative **5** (0.24 mmol) were dissolved in 7 mL of DMF, then EDC*HCl (37.3 mg, 0.24 mmol) and DMAP (29.3 mg, 0.24 mmol) were successively added to the resulting solution at 0 °C and stirred for 20 min. Then the reaction was brought to room temperature and CPT (41.80 mg, 0.12 mmol) was added and stirred at rt. under (Ar) atmosphere for 48 h. Upon completion, the resulting reaction mass was filtered and concentrated. The resulting crude product was purified by column chromatography on SiO_2_ (EtOAc) to obtain conjugate **14**.

(S)-4-ethyl-3,14-dioxo-3,4,12,14-tetrahydro-1H-pyrano[3′,4′:6,7]indolizino[1,2-b]quinolin-4-yl-3α-(hydroxysuccinyl)oxy-5β-cholan-24-oate (**14**). Light yellow solid (167.4 mg, 85% yield). ^1^H NMR (300 MHz, Chloroform-*d*) δ 8.40 (s, 1H), 8.27 (d, *J* = 8.5 Hz, 1H), 7.95 (d, *J* = 8.2 Hz, 1H), 7.84 (t, *J* = 7.7 Hz, 1H), 7.68 (t, *J* = 7.5 Hz, 1H), 7.34 (s, 1H), 5.69 (d, *J* = 17.2 Hz, 1H), 5.40 (d, *J* = 17.3 Hz, 1H), 5.28 (s, 1H), 4.75–4.54 (m, 2H), 3.67 (s, 3H), 2.97–2.74 (m, 2H), 2.73–2.53 (m, 2H), 2.45–2.03 (m, 2H), 2.00–0.95 (m, 30H), 0.90 (d, *J* = 6.1 Hz, 3H), 0.79 (s, 3H), 0.68 (d, *J* = 13.8 Hz, 1H), 0.62 (s, 3H). ^13^C NMR (75 MHz, CDCl_3_) δ 174.80, 171.51, 171.41, 167.43, 157.41, 152.39, 148.83, 146.09, 131.21, 130.68, 129.71, 128.52, 128.22, 128.18, 128.05, 120.08, 96.63, 76.24, 74.96, 67.02, 56.41, 55.97, 51.50, 49.95, 42.72, 41.81, 40.31, 40.07, 35.77, 35.37, 34.81, 34.44, 32.13, 31.76, 31.08, 31.03, 29.25, 29.02, 28.19, 27.02, 26.45, 26.29, 24.18, 23.20, 20.79, 18.29, 12.04, 7.66. MS (ESI): *m*/*z* Calcd for C_49_H_60_N_2_O_9_[M + Na] ^+^: 843.4191; Found: 843.4198.

Procedure for the synthesis conjugate **17**. To a stirred solution of acid **16** (120 mg, 0.221 mmol) in 30 mL of anhydrous CH_2_Cl_2_, followed by *N*-[3-(methylamino)propyl]-*N*’-ethylcarbodiimide hydrochloride (EDC·HCl) (35.0 mg, 0.221 mmol) and 4-dimethylaminopyridine (DMAP) (27.0 mg, 0.221 mmol) were successively added to resulting solution at 0 °C and stirred for 20 min. at 0 °C. Then the reaction was brought to rt. and CPT (camptothecin) (38.32 mg, 0.11 mmol) was added and stirred at r.t. under (Ar) atmosphere for 24 h. Upon completion, the resulting reaction mass was filtered and concentrated. The resulting crude product was purified by column chromatography on SiO_2_ (EtOAc) to obtain conjugate **17**.

(S)-4-ethyl-3,14-dioxo-3,4,12,14-tetrahydro-1H-pyrano[3′,4′:6,7]indolizino[1,2-b]quinolin-4-yl(3β)-3-(oxo)lup-20(29)-en-28-O-hemisuccinate (**17**). Light yellow solid (134.6 mg, 70% yield). ^1^H NMR (300 MHz, Chloroform-*d*) δ 8.43 (s, 1H), 8.30 (d, *J* = 8.6 Hz, 1H), 7.96 (d, *J* = 8.1 Hz, 1H), 7.86 (t, *J* = 7.7 Hz, 1H), 7.69 (t, *J* = 7.5 Hz, 1H), 7.37 (s, 1H), 5.69 (d, *J* = 17.2 Hz, 1H), 5.41 (d, *J* = 17.2 Hz, 1H), 5.30 (s, 2H), 4.66 (d, *J* = 2.3 Hz, 1H), 4.58 (s, 1H), 4.22–4.07 (m, 2H), 3.99–3.89 (m, 1H), 3.50 (s, 1H), 2.95–2.83 (m, 2H), 2.75–2.64 (m, 2H), 2.56–2.26 (m, 3H), 2.26–2.09 (m, 1H), 2.06 (s, 1H), 2.02–1.87 (m, 1H), 1.87–1.68 (m, 2H), 1.65 (s, 3H), 1.62–1.48 (m, 4H), 1.44–1.14 (m, 12H), 1.13–0.93 (m, 10H), 0.89 (d, *J* = 10.3 Hz, 6H), 0.81 (s, 3H). ^13^C NMR (75 MHz, CDCl_3_) δ 218.02, 172.18, 171.38, 167.38, 157.35, 152.29, 150.05, 148.62, 145.95, 131.41, 130.80, 129.47, 128.58, 128.25, 128.20, 128.16, 120.28, 109.88, 96.69, 76.24, 67.03, 63.12, 54.91, 49.96, 49.63, 48.70, 47.67, 47.34, 46.36, 42.66, 40.70, 39.58, 37.56, 36.81, 34.49, 34.14, 33.36, 31.80, 29.63, 29.54, 29.01, 28.98, 26.93, 26.61, 25.11, 21.20, 21.07, 19.57, 19.09, 15.90, 15.53, 14.61, 7.64. MS (ESI): *m*/*z*: Calcd for C_54_H_66_N_2_O_8_ [M + H]^+^: 871.4892; Found: 871.4895.

General procedure for the synthesis of derivatives **24** and **25**. To a stirred solution of **21** (**22**) (271. 4 mg, 0.5 mmol) and DMAP (74.03 mg, 0.6 mmol) in dry CH_2_Cl_2_ (15 mL) was added CPT (camptothecin) at room temperature. The resulting reaction was refluxed for 10 h. After completion of the reaction, the solvent was evaporated under reduced pressure. The residue was diluted with EtOAc (25 mL) and washed with water (2 × 20 mL) and saturatedNaCl solution (2 × 20 mL). The organic layer was dried over MgSO_4_ the solvent was evaporated. The resulting crude product was purified by column chromatography on SiO_2_ using petroleum ether/EtOAc = 4/1 as the elution solvent to afford derivatives **24** and **25**.

(S)-4-ethyl-3,14-dioxo-3,4,12,14-tetrahydro-1H-pyrano[3′,4′:6,7]indolizino[1,2-b]quinolin-4-(3β)-3-(acetyloxy)urs-12-en-28-carbonyloxyethyl)hemisuccinate (**24**). Yellow solid (350.1 mg, 72% yield). ^1^H NMR (300 MHz, Chloroform-*d*) δ 8.42 (s, 1H), 8.25 (d, *J* = 8.6 Hz, 1H), 7.96 (d, *J* = 8.1 Hz, 1H), 7.85 (t, *J* = 7.7 Hz, 1H), 7.69 (t, *J* = 7.6 Hz, 1H), 7.33 (s, 1H), 5.69 (d, *J* = 17.3 Hz, 1H), 5.40 (d, *J* = 17.3 Hz, 1H), 5.30 (s, 2H), 5.22 (d, *J* = 3.5 Hz, 1H), 4.48 (t, *J* = 7.9 Hz, 1H), 4.36–4.00 (m, 6H), 2.97–2.78 (m, 2H), 2.68 (t, *J* = 7.5 Hz, 2H), 2.40–2.09 (m, 2H), 2.05 (s, 3H), 1.82–0.87 (m, 25H), 0.93 (d, *J* = 6.7 Hz, 8H), 0.85 (d, *J* = 4.6 Hz, 10H), 0.70 (s, 3H). ^13^C NMR (75 MHz, CDCl_3_) δ 177.27, 171.55, 171.24, 171.04, 167.34, 157.39, 152.31, 148.65, 146.03, 138.12, 131.45, 130.87, 129.45, 128.62, 128.30, 128.25, 128.16, 125.59, 120.24, 96.54, 80.97, 76.35, 67.05, 62.69, 61.81, 55.35, 52.85, 50.01, 48.16, 47.51, 42.06, 39.59, 39.07, 38.87, 38.33, 37.72, 36.89, 36.64, 33.00, 31.83, 30.65, 29.74, 28.95, 28.81, 28.12, 27.98, 24.17, 23.60, 23.54, 23.35, 21.36, 21.19, 18.23, 17.07, 17.04, 16.78, 15.55, 7.68. MS (ESI): *m*/*z* Calcd for C_58_H_72_N_2_O_11_ [M + Na] ^+^: 995.5028; Found: 995.5021.

(S)-4-ethyl-3,14-dioxo-3,4,12,14-tetrahydro-1H-pyrano[3′,4′:6,7]indolizino[1,2-b]quinolin-4-(3β)-3-(acetyloxy)olean-12-en-28-carbonyloxyethyl)hemisuccinate (**25**). Yellow solid (340.4 mg, 70% yield) ^1^H NMR (300 MHz, Chloroform-*d*) δ 8.42 (s, 1H), 8.25 (d, *J* = 8.5 Hz, 1H), 7.96 (d, *J* = 8.2 Hz, 1H), 7.85 (t, *J* = 7.7 Hz, 1H), 7.68 (t, *J* = 7.5 Hz, 1H), 7.31 (s, 1H), 5.69 (d, *J* = 17.2 Hz, 1H), 5.40 (d, *J* = 17.2 Hz, 1H), 5.31–5.21 (m, 3H), 4.48 (t, *J* = 7.9 Hz, 1H), 4.36–4.04 (m, 5H), 2.94–2.78 (m, 3H), 2.73–2.61 (m, 2H), 2.36–2.09 (m, 2H), 1.97–1.77 (m, 3H), 1.69–1.32 (m, 9H), 1.31–1.21 (m, 3H), 1.11 (s, 3H), 1.01 (t, *J* = 7.4 Hz, 3H), 0.95–0.80 (m, 15H), 0.69 (s, 3H). ^13^C NMR (75 MHz, CDCl_3_) δ 177.48, 171.52, 171.21, 171.04, 167.60, 157.38, 152.33, 148.68, 146.10, 146.01, 143.65, 131.40, 130.83, 129.48, 128.61, 128.29, 128.23, 128.14, 122.42, 120.19, 96.47, 80.96, 77.53, 77.10, 76.68, 76.34, 67.04, 62.65, 61.86, 55.34, 50.00, 47.55, 46.78, 45.83, 41.70, 41.28, 39.35, 38.14, 37.72, 36.94, 33.85, 33.09, 32.69, 32.38, 31.82, 30.70, 28.92, 28.78, 28.07, 27.63, 25.84, 23.62, 23.56, 23.44, 22.98, 21.35, 18.23, 16.93, 16.71, 15.39, 7.66. MS (ESI): *m*/*z* Calcd for C_58_H_72_N_2_O_11_ [M + Na] ^+^: 995.50288; Found: 995.5028.

Procedure for the synthesis of conjugate **26**. Acid **23** (210 mg, 0.35 mmol) was dissolved in 5 mL of DMF, followed by *N*-[3-(methylamino)propyl]-*N*′-ethylcarbodiimide hydrochloride (EDC·HCl) (54.0 mg, 0.35 mmol) and DMAP (43.0 mg, 0.35 mmol) were successively added to resulting solution at 0 °C and stirred for 20 min. at 0 °C. Then the reaction was brought to rt. and CPT (camptothecin) (61.0 mg, 0.175 mmol) was added and stirred at rt. under (Ar) atmosphere for 48 h. Upon completion, the resulting reaction mass was filtered and concentrated. The resulting crude product was purified by column chromatography on SiO_2_ (EtOAc) to obtain conjugate **26**.

(S)-4-ethyl-3,14-dioxo-3,4,12,14-tetrahydro-1H-pyrano[3′,4′:6,7]indolizino[1,2-b]quinolin-4-yl (3β)-3-(oxo)lup-20(29)-en-28-carbonyloxyethyl)hemisuccinate (**26**). Light yellow solid (240.83 mg, 80% yield). ^1^H NMR (300 MHz, Chloroform-*d*) δ 8.43 (s, 1H), 8.26 (d, *J* = 8.5 Hz, 1H), 7.96 (d, *J* = 8.2 Hz, 1H), 7.86 (t, *J* = 7.7 Hz, 1H), 7.69 (t, *J* = 7.5 Hz, 1H), 7.34 (s, 1H), 5.69 (d, *J* = 17.3 Hz, 1H), 5.40 (d, *J* = 17.3 Hz, 1H), 5.30 (s, 2H), 4.72 (s, 1H), 4.60 (s, 1H), 4.41–4.25 (m, 2H), 4.25 (s, 2H), 3.03–2.91 (m, 1H), 2.91–2.83 (m, 2H), 2.74–2.63 (m, 2H), 2.54–2.10 (m, 6H), 1.94–1.81 (m, 3H), 1.60 (t, *J* = 11.3 Hz, 1H), 1.50–1.34 (m, 11H), 1.34–1.24 (m, 6H), 1.09–0.87 (m, 20H). ^13^C NMR (75 MHz, CDCl_3_) δ 218.10, 175.83, 171.53, 171.23, 167.32, 157.40, 152.29, 150.39, 148.61, 146.02, 131.51, 130.91, 129.40, 128.64, 128.32, 128.26, 128.20, 120.31, 109.79, 96.62, 77.52, 77.10, 76.68, 76.37, 67.07, 62.79, 61.53, 56.62, 55.07, 50.02, 49.96, 49.40, 47.39, 46.99, 42.52, 40.70, 39.68, 38.43, 36.97, 34.20, 33.69, 32.06, 31.85, 30.61, 29.64, 28.97, 28.82, 26.66, 25.60, 21.47, 21.09, 19.69, 19.41, 15.97, 15.81, 14.68, 7.67. MS (ESI): *m*/*z* Calcd for C_56_H_68_N_2_O_10_ [M + Na] ^+^: 951.4766; Found: 951.4771.

### 2.3. Cells Culturing

A series of human cell lines, including Jurkat, HCT 116, A549, HL60, K562, HeLa, HEK293, and WI-38, were obtained from the European Authentic Cell Culture Collection. These cell lines were then cultivated in accordance with well-established standard protocols and sterile methods. Cells were cultivated in RPMI 1640 (for Jurkat, HL60, and K562) and DMEM (for HCT 116, A549, HeLa, HEK293, and Fibroblasts) media (Gibco, Thermo Fisher Scientific, Waltham, MA, USA), with the addition of 4 μM glutamine, 10% FBS (Sigma-Aldrich, St. Louis, MO, USA), and 100 U/mL penicillin-streptomycin (Sigma). All cell types were cultivated in a humidified atmosphere containing 5% CO_2_ at a temperature of 37 °C. Subculturing was performed at two- to three-day intervals. Subsequently, the cells were seeded into 24-well plates at a density of 5 × 10^4^ cells per well and incubated overnight. The cells were sub-cultured at two-day intervals at a seeding density of 1 × 10^5^ cells per 24-well plate in RPMI medium with 10% FBS.

### 2.4. Preparation of Effluents and Solutions of Test Compounds for Biological Testing

The dissolution of the compounds tested was initially performed in DMSO with an initial solution of 100 mM in 10% DMSO. The solution was subsequently diluted in complete culture medium, namely Dulbecco’s medium, modified Eagle’s medium (DMEM), or Roswell Park Memorial Institute medium (RPMI). Substances were added at concentrations of 10, 1, 100, 10, 1, and 0.1 μM on the day after seeding and incubated for 24 h. Subsequently, the cells were washed with PBS solution and stained with 7AAD dye in accordance with the manufacturer’s protocol for flow cytometry. The CC_50_ value, which characterizes parameters of cellular toxicity (i.e., the concentration of the compound required for 50% inhibition of cell viability in vitro), was calculated. The logC versus % inhibition was plotted, and statistical data processing was performed using Excel and GraphPad Prism v.8.0.2 (San Diego, CA, USA, 2019). The data obtained from these three independent experiments were expressed as the mean of three measurements for each concentration, with standard deviation indicated as well. These values were then relative to control values (0.1% DMSO), which were taken as 100%.

### 2.5. Cytotoxicity Assay

The assessment of cell viability was conducted by employing 7-AAD dye (eBioscience, San Diego, CA, USA). Following the incubation of the cells with the test compounds, the cells were harvested, washed with PBS, and subjected to centrifugation at 400 g for five minutes. The cell sediment was resuspended in 200 μL of staining buffer for flow cytometry (PBS without calcium and magnesium, 2.5% FBS) and stained with 1 mM 7-AAD dye solution for 15 min in the dark at room temperature. Subsequently, all experimental and control samples were analyzed on a BD FACSAria™ III Cell Sorter flow cytometer (Becton, Dickinson and Company, Franklin Lakes, NJ, USA).

### 2.6. Apoptosis Assay

The process of apoptosis was determined by means of a flow cytometric analysis of annexin V and 7-aminoactinomycin D staining. In summary, 200 µL of Guava Nexin reagent (Millipore, Bedford, MA, USA) was added to 5 × 10^5^ cells in 200 µL, and the cells were incubated with the reagent for 20 min at room temperature in the dark. The plates were treated with compounds at various concentrations for a period of 24 h. At the conclusion of the incubation period, the cells were analyzed using a BD FACSAria™ III Cell Sorter flow cytometer (Becton, Dickinson and Company, Franklin Lakes, NJ, USA). The various states of cell death were delineated as such: normal cells were localized in the lower-left quadrant (Annexin V−/PI−); early apoptotic cells were in the lower-right quadrant (Annexin V+/PI−); late apoptotic cells and necrotic cells were in the upper-right quadrant (Annexin V+/PI+); and necrotic cells were in the upper-left quadrant (Annexin V−/PI+).

### 2.7. Cell Cycle Analysis

The cell cycle was analyzed using the method of propidium iodide staining. In summary, the cells were cultivated in 24-well round-bottom plates at a density of 10^5^ cells per well. Then, the plates were rotated at 450× *g* for 5 min. Following this, the cells were fixed with ice-cold 70% ethanol for 24 h at 0 °C. Subsequently, the cells were washed with phosphate-buffered saline (PBS) and then incubated with 250 µL of Guava Cell Cycle Reagent (Millipore, Burlington, MA, USA) for a period of 30 min at room temperature and in the dark. The samples were then subjected to analysis on the NovoCyteTM 2000 Flow Cytometry System (ACEA, San Diego, CA, USA).

### 2.8. Topoisomerase I Assay

The procedure for measuring the activity of topoisomerase I and its subsequent inhibition by synthesized hybrid molecules was conducted in accordance with the method previously described [24].

### 2.9. In Silico Evaluation of Synthesized Compounds (ADME)

The Swiss Institute of Bioinformatics (SIB) offers an online tool, SwissADME (Swiss Institute of Bioinformatics, Lausanne, Switzerland, version 3.0), which is in the public domain. This tool integrates a range of computational methodologies, providing a comprehensive evaluation of the pharmacokinetic profile and drug properties of small compounds. The tool is available at http://www.swissadme.ch (accessed on 5 June 2026) [25]. The methodology was employed to ascertain the potential efficacy and suitability of newly developed compounds in terms of their pharmacokinetics.

### 2.10. Statistica

The experimental protocol was replicated thrice, with each repetition comprising six independent measurements. All statistical calculations were presented as mean ± standard deviation (SD) using GraphPad Prism^®^ software (version 6.0, GraphPad Software Inc., San Diego, CA, USA). The statistical significance of the data was determined by the *p*-value, with results deemed to be significant if *p* < 0.05, and highly significant if *p* < 0.01 or *p* > 0.001. The presence of *** (*p* < 0.001) indicates the significance of observed differences between values obtained in samples treated with synthesized substances and control compounds (comparison was performed using one-way ANOVA analysis of variance).

## 3. Results and Discussion

### 3.1. Chemistry

**Scheme 1:**
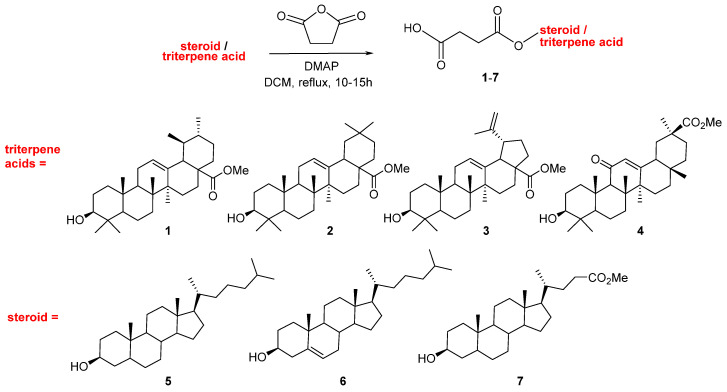
Synthesis of hemisuccinate compounds (**1**–**7**).

The strategy developed for the preparation of hybrid molecules involved the preparation of cholestanolhemisuccinates **5**, cholesterol **6**, and methyl esters of lithocholic **7** (natural lithocholic acid has an α hydroxyl configuration at atom 3, 3β-hydroxy in the scheme) and pentacyclic triterpene acids **1**–**4** by reacting succinic anhydride with the hydroxyl group in the presence of DMAP (DCM, boiling, 10–15 h) in the first step. (Scheme 1).

**Scheme 2:**
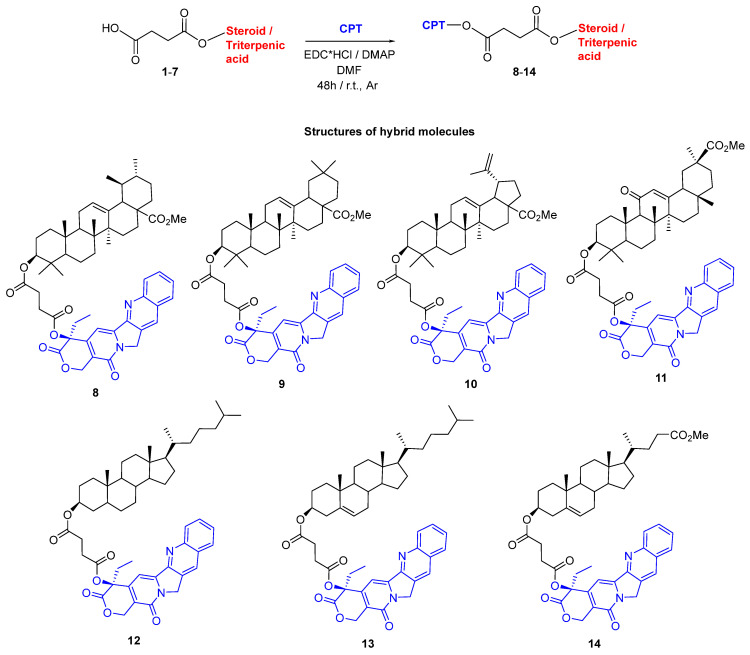
Synthesis of hybrid molecules (**8**–**14**).

In the second step of the process, the esterification of CPT by the hydroxyl group at the C-4 carbon atom of the lactone fragment with hemisuccinate **1**–**7** was carried out in the presence of EDC·HCl and DMAP under argon atmosphere (DMF, room temperature, 48 h). This process resulted in the formation of the target hybrid molecules (**8**–**14**). (Scheme 2).

**Scheme 3:**
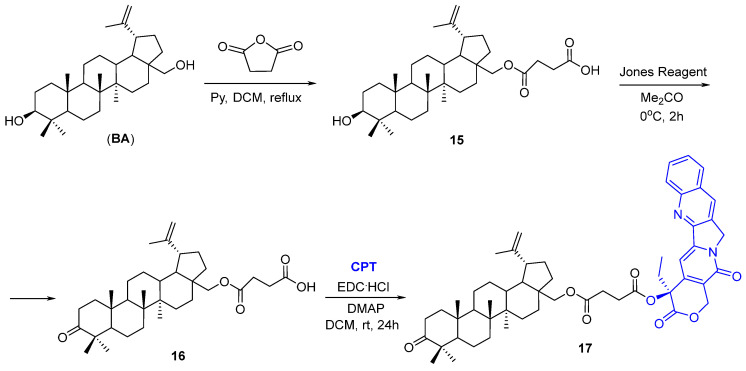
Synthesis of a hybrid molecule **17**.

A similar ideology was implemented during the preparation of a hybrid molecule based on camptothecin and betulin. However, in contrast to hybrid molecule **10**, the modification was carried out on the hydroxyl group at C-28 of the triterpene cortex (Scheme 3).

The synthesis of betulin succinate **15** commenced with the esterification of the hydroxyl group with succinic anhydride, a reaction that was carried out using a combination of pyridine, dichloromethane, and a reflux process. The oxidation of compound **15** with Jones’ reagent in acetone at 0 °C for 2 h resulted in the quantitative yield of the betulonic acid derivative, ketone **16**. The latter interaction with camptothecin led to the target hybrid molecule **17**. Concurrently, it was demonstrated that replacing DMF with DCM as the solvent resulted in a reduction of the reaction time from 48 h to 24 h (Scheme 3).

**Scheme 4:**
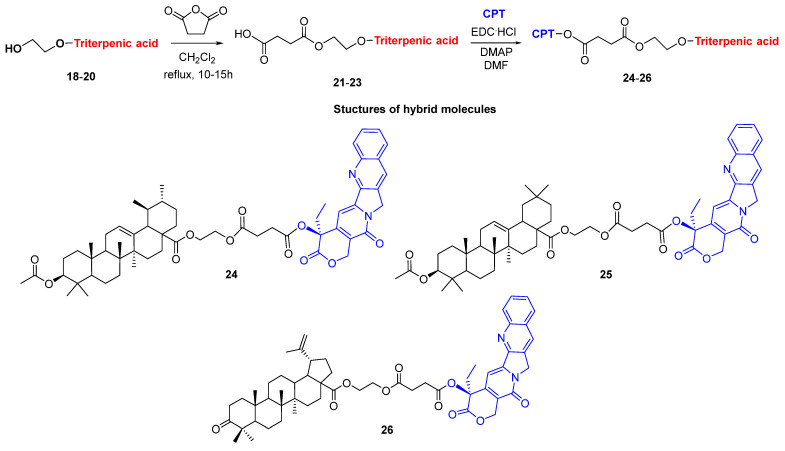
Synthesis of hybrid molecules (**24**–**26**).

In the course of these studies, we developed a synthetic approach to obtain hybrid molecules **24**–**26** that contain fragments of pentacyclic triterpene acids (ursolic, oleanolic, and betulinic acids) in their structures. The synthesis of the target compounds was carried out in three steps. First, monoacylation of ethylene glycol with the chlorohydrides of the triterpene acids was performed. The hydroxyl group was protected at C-3 and obtained in situ by the action of oxalyl chloride, according to the method described in the literature [26,27,28]. The second step was the esterification of the obtained alcohols (**18**–**20**) with succinic anhydride in the presence of DMAP to form acids (**21**–**23**). The final step was the addition of camptothecin to obtain the target hybrid molecules (**24**–**26**) (Scheme 4). The choice of succinic acid linkers and lipophilic carriers plays a strategic role in both optimizing reaction efficiency and expanding structural diversity. Across compounds **8**–**14**, the two-step direct conjugation via hemisuccinates achieved high conversion rates and yields up to 92% (e.g., for cholestanol hybrid **12**). In the case of betulin derivative **17**, optimizing the synthetic protocol by substituting DMF with DCM significantly halved the reaction time from 48 to 24 h while fully preserving product yields. For the more structurally complex triterpene series (**24**–**26**), a three-step spacer elongation utilizing an ethylene glycol-succinate bridge successfully overcame steric hindrance issues associated with pentacyclic triterpene acid chlorohydrides. From a physicochemical standpoint, inserting these flexible succinic and glycol spacers modifies the rigid, planar aromatic pentacyclic core of native CPT, noticeably facilitating its dissolution in liquid media and improving intracellular uptake.

### 3.2. Study of Biological Activity of Compounds

**Figure 3:**
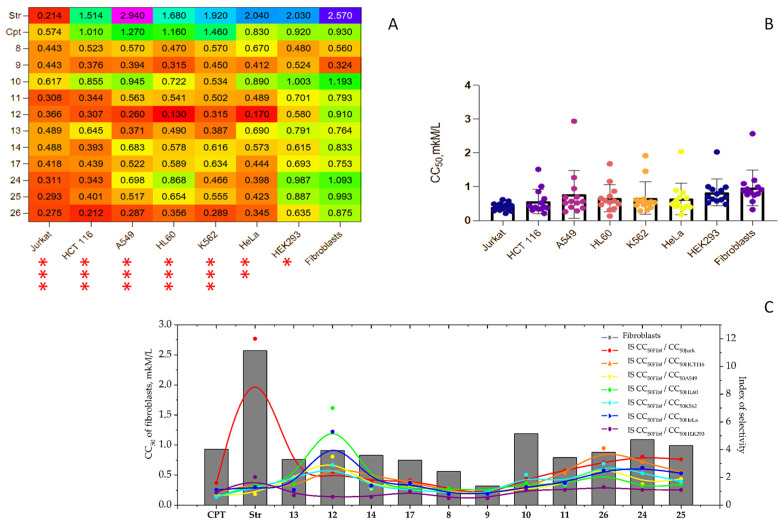
The cytotoxic activity (CC_50_) of the synthesized compound was evaluated in vitro in cell cultures (Jurkat, HCT 116, A549, HL60, K562, HeLa, HEK293 and Fibroblasts). The cytotoxicity values of the compounds under investigation are illustrated in the heat map (**A**). The median cytotoxicity values for the compounds across nine cell lines are also represented (**B**). Diagram (**C**) depicts the ratios of selectivity indices for the compound library under study. All synthesized compounds were injected into the cell samples at a concentration corresponding to the CC50 value and the incubation period was 24 h. Data were normalized to the percentage of viable cells in the control group (DMSO control) and presented as the mean ± standard deviation (SD) of three independent experiments. Statistical analysis was conducted using one-way ANOVA followed by Tukey’s post hoc test to compare the control and treated groups. * *p* < 0.05, ** *p* < 0.01, and *** *p* < 0.001, not significant.

The study demonstrated that the obtained compounds exhibited a broad spectrum of antitumor activity against eight distinct cell lines (Jurkat, HCT 116, A549, HL60, K562, HeLa, HEK293, and Fibroblast) at concentrations ranging from 0.275 ± 0.03 to 1.193 ± 0.03 µM. These findings substantiate the compounds’ selectivity for antitumor action, as evidenced by the variation in selectivity index (SI) (Table S1, Figure 3). Structural variations among the lipophilic carrier vectors dictate distinct selectivity profiles across different cancer cell lines relative to normal fibroblasts [29]. The cholestanol vector in compound **12** drives prominent growth inhibition and high selectivity in A549, HL60, K562, and HeLa lines, reaching exceptional Selectivity Index (SI) values of 7.0 for HL60 and 5.3 for HeLa. Conversely, shifting the modification to the C-28 position of betulin in hybrid 26 alters the target spectrum, showing enhanced selectivity toward HCT116 (SI = 4.1), A549 (SI = 3.0), and K562 (SI = 3.0). The enhanced cellular uptake and selectivity observed for several hybrid conjugates—particularly those bearing bulky steroidal or triterpenoid domains—are hypothesized to be driven by lipid raft-mediated endocytosis. While direct mechanistic internalization assays were not performed in this study, this assumption is strongly supported by literature precedents. Similar lipophilic small-molecule drug conjugates utilizing cholesterol or pentacyclic triterpenes have been experimentally demonstrated to partition preferentially into liquid-ordered membrane microdomains (lipid rafts), triggering safe caveolae- or clathrin-independent endocytic pathways [30,31,32].

For the purpose of comparison, camptothecin and staurosporine were selected as the standard drugs. These drugs are considered the gold standard for testing the effects of topoisomerase I inhibition and apoptosis induction [33]. The research group has conducted exhaustive studies on the mechanisms of action of camptothecin and staurosporine [22,34]. The mechanism of action of the hybrid molecules under study is likely a genotoxic effect analogous to that of the parent compound camptothecin. However, the incorporation of a steroidal or triterpenoid pharmacophore, in conjunction with a glycol bridge and a succinic acid fragment as a spacer [35], facilitates the dissolution of the compounds in liquid media, enhances their penetration into cells, and increases their selectivity of action in comparison to camptothecin (see Table S1 in the Supplementary Materials).

**Figure 4:**
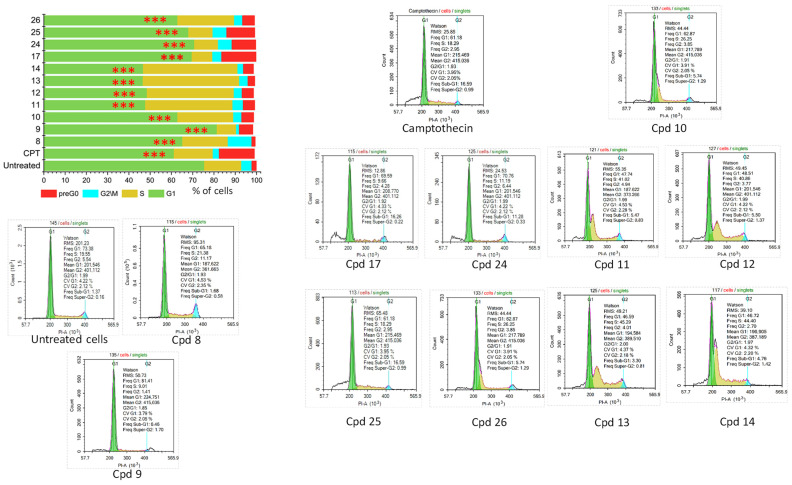
The effects of synthesized hybrid molecule compounds on the distribution of G_0_/G_1_, S, and G_2_/M cell cycle phases in Jurkat cell line control cells, as well as after treatment with the compounds at a concentration of 0.5 × CC_50_, were assessed. Detection was conducted using flow cytometry. The results are based on three independent experiments, and cell cycle histograms are provided in the Supplementary Information File. Statistical analysis was conducted using one-way ANOVA followed by Tukey’s post hoc test to compare the control sample. *** *p* < 0.001.

In numerous preclinical studies, the knockdown of DNA synthesis by camptothecin derivatives has exhibited sustained regression of tumor tissue [36]. Recent studies have revealed details of camptothecin’s action on the cell, including the ability to inhibit mitochondrial DNA, further establishing CPT and its derivatives as a pleiotropic regulator of DNA synthesis [36,37]. Given that cell proliferation is subject to regulation by the cell cycle, the effect of hybrid molecules on the distribution of cell cycle phases in Jurkat cells was studied using flow cytometric analysis. The antiproliferative activity of the synthesized hybrid molecules was evaluated against the p53-deficient Jurkat cell line. A subsequent analysis of the underlying molecular mechanisms revealed that the hybrid molecules exhibited the capacity to impede Jurkat proliferation by inducing cell cycle arrest and apoptosis. Notably, the hybrid molecules demonstrated enhanced solubility in aqueous media, a property that distinguishes them from camptothecin. As demonstrated in Figure 4, Jurkat cells exposed to hybrid molecules **17** (betulinic acid) and **25** (oleanolic acid) for 48 h exhibited a significantly higher percentage of cells in sub-G_1_ phase (16.26% and 16.59%), whereas compound **26** exhibited an elongated S phase (26.5%). Furthermore, compounds **17**, **24**, and **25** exhibited a diminished population of cells in the G_2_/M phase, with percentages of 4.28%, 2.95%, and 6.44%, respectively. A comparison of samples **17**, **24**, and **25** with camptothecin revealed no significant disparities in any of the cell cycle phases. However, a statistically significant difference (*p* < 0.001) from the control sample was observed in all three samples. Compound **9**, a hybrid of camptothecin and oleanolic acid, exhibited a significant impact on all phases of the cell cycle. The percentage of cells in the S-phase and G_2_/M phase was reduced to 9.01% and 1.41%, respectively. In contrast, the percentage of cells in the G_1_ phase increased to 81.41%, which was higher than in the control sample and the sample incubated with camptothecin (*p* < 0.001). Treatment of cells with compounds **11**–**14** for 48 h resulted in a markedly higher percentage of cells in S phase (41.02%, 40.86%, 45.29%, and 44.40%, respectively) and a lower cell population in G_2_/M phase (4.94%, 3.77%, 4.01%, and 2.70%, respectively). These results were statistically significant when compared with the control sample (Figure 4). Consequently, hybrids of CPT with triterpenoids (betulonic, oleanolic, ursolic acids) bound at the C-28 position of carbon atom **17**–**26** exhibit a comparable effect on the DNA of Jurkat cells, leading to cell cycle arrest at the S-phase level. This action is analogous to that of camptothecin, with no significant differences observed between the two compounds. The group of compounds **11**–**14** are hybrid CPT molecules with lithocholic, 18β-glycyrretinic acids, and cholestanol vectors act mainly on all phases of the cell cycle, causing an increase in the hypodiploid cell population (Figure 4).

**Figure 5:**
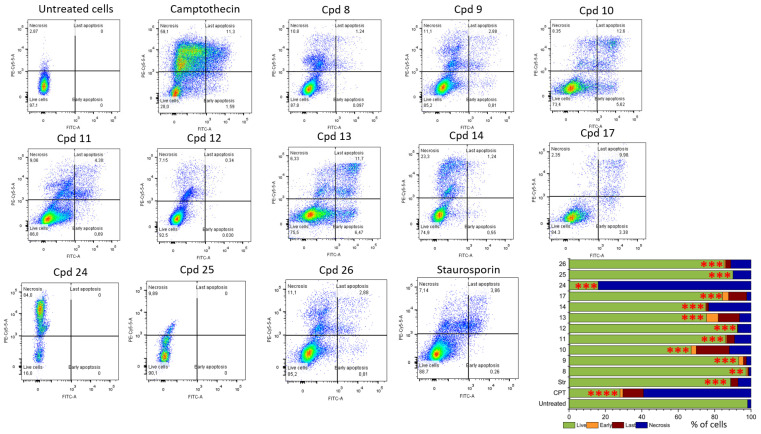
Apoptosis phase distribution in Jurkat cell culture after treatment with synthesized hybrid molecules at a concentration of 0.5CC_50_. The determination was carried out by flow cytometry. Three independent experiments were used to generate the results, and a Supplementary Information File includes histograms of the distribution of cell populations in different apoptosis phases. Statistical analysis was conducted using one-way ANOVA followed by Tukey’s post hoc test to compare the control sample. ** *p* < 0.01, *** *p* < 0.001, and **** *p* < 0.0001.

Furthermore, all synthesized hybrid molecules have been demonstrated to induce apoptosis. Apoptosis is a regulated process that leads to cell self-destruction. It has been demonstrated that any defects in the process of apoptosis can lead to increased malignization and resistance to chemotherapeutic treatment. The vast majority of neoplastic diseases are associated with either an absence or an insufficient degree of apoptosis [38]. In the course of analyzing apoptosis in the Jurkat cell line, it was observed that a considerable percentage of cells do not undergo death within the initial 24 h period of incubation. This observation indicates that the apoptosis process is likely to be relatively slow in the majority of compounds, with the exception of camptothecin and compound **24**, which is a hybrid of CPT with ursolic acid. A comparison of the number of cells in the secondary necrosis stage, in addition to camptothecin and compound **26**, reveals that the latter two compounds result in necrosis processes and the smallest number of cells in the G_1_ phase. Notably, samples **8**, **9**, **14**, and **26** (23.3%, 11.1%, 12.1%, and 10.8% of cells in the secondary necrosis stage, respectively) merit particular consideration. The initial stage of apoptosis was most prevalent in samples **10**, **13**, and **17**, with percentages of 5.82%, 6.47%, and 3.38%, respectively. In comparison with camptothecin, these compounds demonstrate a comparable effect and exhibit no substantial disparities in the phases of early and late apoptosis. However, in contrast to the control compound, they accumulate the population of cells in the secondary necrosis stage at a significantly slower rate (*p* > 0.005) (Figure 5).

**Figure 6:**
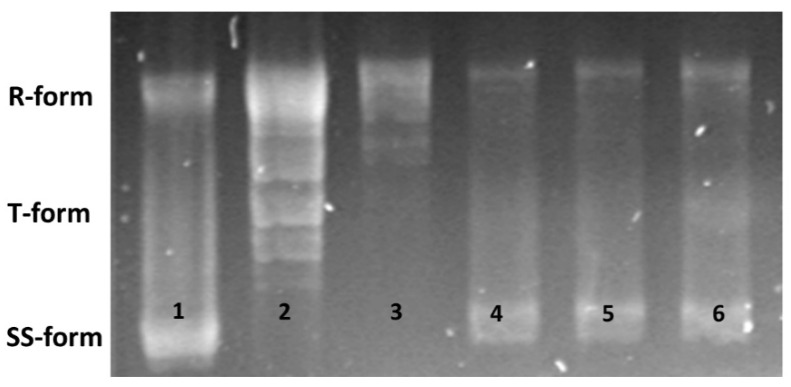
Inhibition of the catalytic activity of human (hTOPOI) DNA topoisomerase I by camptothecin and synthesized hybrid molecules measured by relaxation. The pHOT1 supercoiled plasmid DNA was relaxed by purified topoisomerase I in the absence (+hTOPOI) or in the presence of compounds at selected concentrations [µM/L]. The resulting topological forms of DNA were separated by gel electrophoresis. SC, supercoiled DNA; R, relaxed DNA; T, DNA topoisomers. The data presented are from three independent experiments. 1. Camptothecin (100 µM); 2. Relaxed pHOT1 plasmid DNA (250 ng); 3. Superhelicalized plasmid pHOT1+ 2 units of TOPOI; 4. Superhelicalized plasmid pHOT1+ 2 units of TOPOI + **9** (150 µM); 5. Superhelicalized plasmid pHOT1+ 2 units of TOPOI + **24** (150 µM); 6. Superhelicalized plasmid pHOT1+ 2 units of TOPOI + **26** (150 µM).

The basis for testing the synthesized compounds for inhibitory activity against topoisomerase I was the ability of the synthesized hybrid molecules to influence the cell cycle and induce apoptosis, with the objective of further confirming their antiproliferative effect and confirming the mechanism of action. Compounds **9**, **24**, and **26** were demonstrated to have a significant inhibitory effect against topoisomerase I (Figure 6). Notably, the strategic introduction of ursolic (**24**) and betulinic (**26**) acid fragments through ethylene glycol-succinate spacers creates a synergistic transport platform. Based on the computational predictions, the structural integration of these lipophilic vectors is hypothesized to favor P-glycoprotein (P-gp) inhibition and lipid raft partitioning. While direct mechanistic assays were not performed, this qualitative rationale aligns with established biomimetic models where bulky lipophilic domains decrease binding affinity toward the P-gp efflux pocket, potentially aiding intracellular retention. Malignant cells have a high metabolic reliance on exogenous lipids, leading to a high density of lipoprotein receptors and lipid rafts. The steroidal and triterpenoid vectors act as biomimetic carriers, exploiting this vulnerability to trigger receptor-mediated and raft-dependent endocytosis, which allows the hybrids to bypass traditional, efflux-susceptible passive diffusion pathways. Once inside, the lipophilic nature of these scaffolds heavily amplifies their capability to serve as P-glycoprotein (P-gp) inhibitors, effectively blocking membrane efflux pumps as confirmed by in silico profiling. This synergistic combination of lipid raft-mediated internalization and P-gp inhibition creates a ‘dual entrapment’ effect, preventing the drug from being pumped out and maintaining the high localized intracellular concentrations required for sustained topoisomerase I entrapment and apoptotic cascade triggering. Taking all in vitro endpoints together, compounds **12** and **26** represent the most balanced and promising scaffolds of the entire library, effectively merging high tumor-specific killing with targeted intracellular mechanism execution.

As demonstrated in Figure 6, compounds **9**, **24**, and **26** exhibit a distinct inhibitory effect on the catalytic activity of human topoisomerase I, as evidenced by the retention of the supercoiled plasmid DNA (SC-form) in lanes 4, 5, and 6. The screening concentration of 150 µM for the intact hybrid conjugates was chosen to establish an upper threshold for direct enzyme engagement. Compared to native CPT, which demonstrates complete topoisomerase I inhibition at 100 µM (lane 1), the intact lipid hybrids require a higher concentration (150 µM) to achieve a comparable level of plasmid relaxation blockade. This shift in direct potency is heavily contextualized by the significant steric bulk introduced by the C-20 steroidal and triterpenoid attachments, confirming that while the intact hybrids maintain direct topoisomerase interaction, their cytotoxic efficacy likely involves intracellular ester cleavage to release free CPT.

### 3.3. Physico-Chemical Properties and ADME Prediction

The ADMET prediction is summarized in Table S2 in the Supplementary Materials. Although the high predicted lipophilicity logD_7_._4_ > 3.5 and low aqueous solubility (<1 mg/L) restrict standard lipid bilayer passive permeability, they directly drive the high affinity of the hybrids toward lipid rafts and P-gp binding sites, translating into potent in vitro cytotoxicity CC_50_ ranging from 0.275 to 1.193 µM. This apparent discrepancy between restricted in silico passive permeability (low aqueous solubility, no human intestinal absorption, and limited bioavailability) and potent in vitro cytotoxicity highlights a fundamental shift in the internalization mechanism. While SwissADME algorithms strictly evaluate classical passive transcellular diffusion pathways, our heavy, amphiphilic conjugates are designed to bypass standard diffusion barriers entirely. The high lipophilicity blocks ordinary passive membrane crossing, shifting the internalization mode toward active, lipid raft-dependent endocytosis. Consequently, low predicted oral bioavailability profiles do not hinder their efficacy, as these lipid-guided hybrids are specifically designed as intravenous therapeutic candidates tailored for localized tumor entrapment rather than oral administration.

Furthermore, ADME algorithms successfully substantiated tissue-protective selectivity, predicting restricted blood–brain barrier (BBB) penetration for the majority of the series (except **17**) to minimize central neurotoxicity, and showing a complete elimination of predicted cardiotoxicity (hERG blockade) specifically for ursolic hybrid **24**. Finally, acute oral toxicity modeling identified compounds, **12** and **26** as low-toxic candidates, perfectly aligning with their superior in vitro therapeutic windows and favorable safety profiles. The resource enables the determination of whether the compound under study functions as a substrate or inhibitor of P-glycoprotein. P-glycoprotein has been identified as a drug transporter, with its primary localization being the membranes of intestinal mucosal cells, hepatocytes, and cells that form the blood–brain barrier. The high activity of P-glycoprotein has been demonstrated to reduce the bioavailability of drugs, decrease their ability to penetrate histohematic barriers, and cause multiple drug resistance of tumor cells. In this context, the in silico profiling suggests that, unlike native camptothecin, the synthesized hybrids are predicted to act as P-glycoprotein inhibitors rather than substrates. This theoretical trend aligns well with known biological models where conjugation with bulky natural lipophilic carriers alters drug recognition by efflux pumps [32], though it remains a prediction that requires future experimental validation via specific transporter assays.

It is worth noting that explicit molecular docking studies were not conducted for the synthesized compounds in this work, representing a limitation that we plan to address in future studies utilizing an expanded library of derivatives. Nevertheless, insights from literature on structurally related C-20 succinyl-linked camptothecin conjugates suggest that the incorporation of bulky lipid domains through succinate spacers introduces profound steric hindrance at the Topoisomerase I-DNA cleavage complex active site [39]. This computational precedent reinforces the rationale that the intact bulky hybrids may exhibit lower direct enzyme affinity compared to native CPT, thus heavily supporting the hypothesis that intracellular metabolic or enzymatic ester hydrolysis is essential to liberate the fully active topoisomerase poison inside tumor cells.

Hepatotoxicity is also characteristic of all synthesized molecules. Also these compounds are capable of causing skin sensitization. These findings do not contradict the characteristics of camptothecin itself and its synthesized derivatives, since all synthesized hybrids have a sufficiently high antitumor potential in the in vitro studies we conducted. The result of mutagenicity assessment in the calculated Ames test was positive for both camptothecin and all hybrid molecules based on it, since irreversible inhibition of topoisomerase I often leads to a mutagenic effect. No potential maximum daily doses were identified when extrapolating the dose results from animals to humans.

## 4. Conclusions

In conclusion, the hybridization of camptothecin with steroidal and triterpenoid pharmacophores via strategic succinic and glycol linkers represents a validated, highly efficient platform for tumor-targeted drug delivery. For the first time, a series of novel camptothecin hybrids featuring various steroidal (cholesterol, cholestanol) and pentacyclic triterpenoid (ursolic, betulinic, and oleanolic acids) vectors were successfully synthesized and evaluated. The strategic design effectively exploits the metabolic dependency of cancer cells on lipophilic compounds and the abundance of lipid rafts on malignant membranes. This vector-guided approach successfully transforms the non-specific systemic toxicity of native camptothecin into tumor-selective cytotoxicity, as demonstrated by the prominent selectivity index values, highlighted by the exceptional performance of cholestanol hybrid **12** (SI = 7.0) and betulin-ethylene glycol hybrid **26** (SI = 4.1), which establish them as the top lead candidates for future multi-drug-resistance-evading chemotherapeutic development. Furthermore, the demonstrated capacity of these hybrids to concurrently inhibit P-glycoprotein efflux pumps and restrict blood–brain barrier penetration ensures sustained intracellular drug entrapment while minimizing central neurotoxicity. Ultimately, this approach opens new avenues for developing safe, site-specific, and multi-drug-resistance-evading chemotherapeutic agents.

## Data Availability

The original contributions presented in this study are included in the article. Further inquiries can be directed to the corresponding authors.

## Supplementary Materials

The following supporting information can be downloaded at https://www.mdpi.com/article/10.3390/pharmaceutics18070867/s1, ^1^H NMR spectra of synthesized products: ^13^C NMR spectra of synthesized products; HRMS/MS spectra of target hybrid molecules; Cytotoxicity of synthesized hybrid molecules against eight cancer cell lines; Physico-chemical properties and in silico ADME prediction parameters.

## Abbreviations

The following abbreviations are used in this manuscript: 7AAD, 7-Aminoactinomycin D; ADMET, Absorption-Distribution-Metabolism-Excretion Toxicity; CPT, Camptothecin; DCM, Dichloromethane; DMAP, 4-Dimethylaminopyridine; DMF, Dimethylformamide; DMSO, Dimethyl sulfoxide; DNA, Deoxyribonucleic Acid; EDC·HCl, N-(3-Dimethylaminopropyl)-N′-ethylcarbodiimide hydrochloride; FBS, Fetal Bovine Serum; HIA, Human intestinal absorption; PBS, Phosphate-buffered saline; SN38, 7-ethyl-10-hydroxycamptothecin; TopI, Human topoisomerase I.
