# Supplementary material for: Novel Camptothecin-Based Lipid Conjugated with Steroids and Triterpene Acids: Enhanced Cellular Uptake and Topoismerase I Inhibition
Source: Pharmaceutics. 2026 Jul 16;18(7):867. doi: 10.3390/pharmaceutics18070867 (PMC13415453; doi:10.3390/pharmaceutics18070867)
Supplement: Supplementary file 1 [file pharmaceutics-18-00867-s001.zip › pharmaceutics-4440478-supplementary.pdf]

Supporting Information for

Novel Camptothecin-Based Lipid Conjugated with Steroids and Triterpene Acids: Enhanced Cellular Uptake and Topoismerase I Inhibition

Nuri M. Chobanov [1], Konstantin O. Vakhnin [2], Stepan E. Logunov [1], Alexander E. Fedorov [1], Georgii V. D'yakonov [1], Usein M. Dzhemilev [1], Lilya U. Dzhemileva [1,*,†] and Vladimir A. D'yakonov [1,*,†]

[1] N.D. Zelinsky Institute of Organic Chemistry, Russian Academy of Sciences, 47 Leninsky Prosp., Moscow 119991, Russia
[2] Institute of Physiologically Active Compounds at Federal Research Center of Problems of Chemical Physics and Medicinal Chemistry, Russian Academy of Sciences (IPAC RAS), Severniy Proezd 1, Chernogolovka 142432, Russia
* Correspondence: dzhemilev@mail.ru (L.U.D.); dyakonovva@rambler.ru (V.A.D.)
† These authors contributed equally to this work.

Table of contents

1. Experimental Section. General. **S2**
2. General procedure for the of derivatives synthesis and hybrid molecules. **S3 – S11**
3. Characterization of all products. **S12 – S25**
4. ^{1}H NMR, ^{13}C NMR and HRMS/MS spectra of all products. **S26 – S61**
5. Table S1. Cytotoxicity of synthesized hybrid molecules **S62**
6. Table S2. Physico-chemical properties and ADME prediction **S63**

2.2. Experimental Section

2.3. General

All commercial reagents were purchased from Sigma-Aldrich and Acros organics. Betulinic acid was prepared from commercially available betulin by a reported procedure [1]. All commercially available solvents and reagents used were of analytical grade and without further purification. Reactions were monitored by TLC on Sorbfil plates. Column chromatography was carried out on Acrus silica gel (0.060–0.200 мм). Optical rotations were measured on a Perkin–Elmer 341 polarimeter. Melting points were recorded on Stuart SMP3.IR spectra were recorded on Bruker VERTEX 70V using KBr discs over the range of 400–4000 cm^{-1}. ^{1}H and ^{13}C NMR spectra were obtained using a Bruker AVANCE 300 spectrometer in $CDCl_3$ operating at 300 MHz for ^{1}H and 75 MHz for ^{13}C. Mass spectra of MALDI TOF/TOF positive ions (matrix of sinapic acid) are recorded on a mass spectrometer BrukerAutoflex^{TM} III Smartbeam. Elemental analyses were measured on 1106 Carlo Erba apparatus.

2.3.1. General procedure for the of derivatives synthesis and hybrid molecules.

2.3.2. General procedure for the of derivatives synthesis with succinic anhydride **1, 2, 4-6.**

To a stirred solution of **steroid or triterpenoic acid** (1 mmol) and DMAP (148.06 mg, 1.2 mmol) in dry CH_2Cl_2 (10 ml), succinic anhydride (400.97 mg, 4 mmol) was added at room temperature. The resulting reaction mixture was refluxed for 10 h. After completion of the reaction, the solvent was evaporated under reduced pressure. The residue was diluted with EtOAc (20 ml) and washed with water (2x20 ml) and brine (2x20 ml).The organic layer was dried over $MgSO_4$, and concentrated. The crude product was purified by column chromatography (silica gel) using petroleum ether/EtOAc = 4/1 as the elution solvent to afford derivatives (**1, 2, 4-6)** was obtained as white solids **yields of** (88 - 95%).

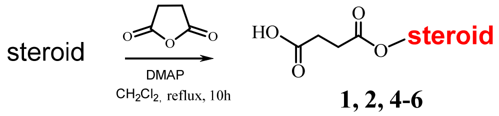

1, 2, 4-6

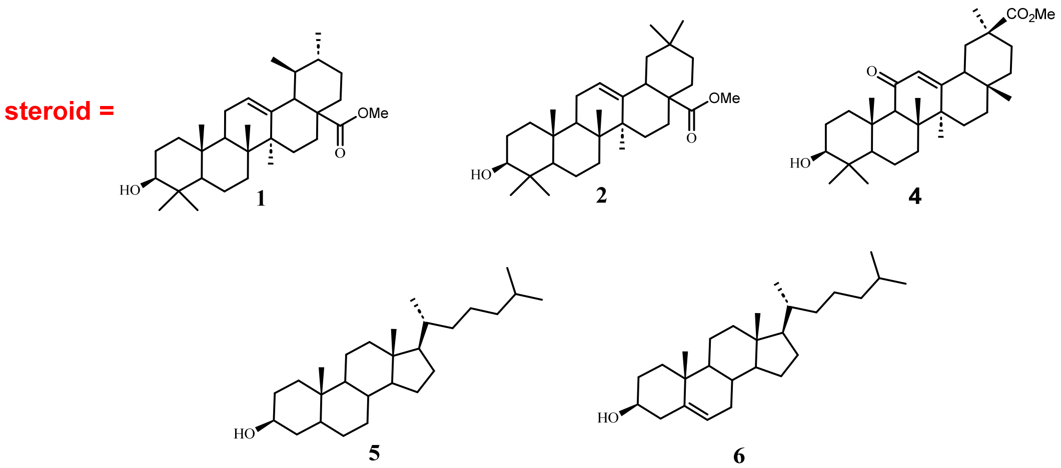

2.3.3. General procedure for the synthesis of conjugates **(8, 9, 11-13)** *with derivatives* (**1, 2, 4-6**) *and camptothecin.*

Derivatives (**1, 2, 4-6**), respectively (0.24 mmol) were dissolved in 5 ml of DMF, then EDC*HCl (37.3 mg, 0.24 mmol) and DMAP (29.3 mg, 0.24 mmol) were successively added to the resulting solution at 0°C and stirred for 15 min. at 0°C. Then the reaction was brought to room temperature and CPT (41.80 mg, 0.12 mmol) was added and stirred at rt. under (Ar) atmosphere for 48 h. Upon completion, the resulting reaction mass was filtered and concentrated. The resulting crude product was purified by column chromatography on SiO_2 (EtOAc) to obtain hybrid molecules **(8, 9, 11-13)**.

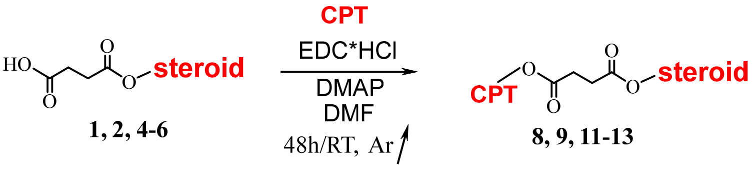

Hybrid molecules

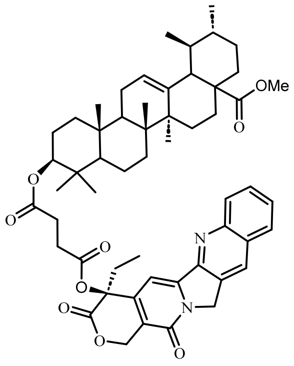

8

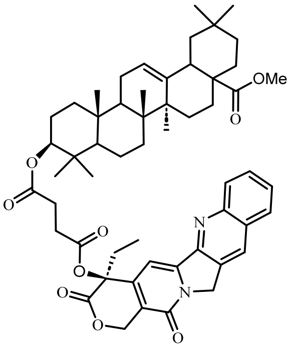

9

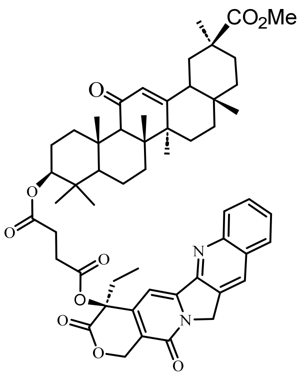

11

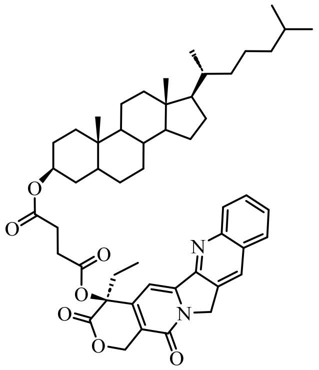

12

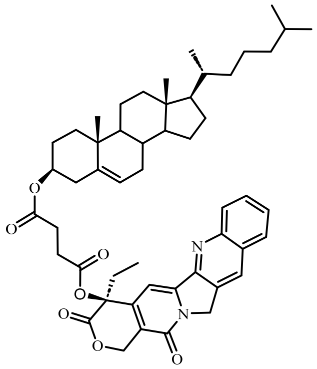

13

*2.3.4. General procedure for synthesis compound **7**.*

To a stirred solution of methyl (4R)-4-((3S,10S,13R)-3-hydroxy-10,13-dimethylhexadecahydro-1H-cyclopenta[a]phenanthren-17-yl)pentanoate (390.6 mg, 1 mmol) and DMAP (1.3 mmol) in dry CH_2Cl_2 (20 ml), succinic anhydride (4.5 mmol) was added at room temperature. The resulting reaction mixture was refluxed for 15 h. After completion of the reaction, the solvent was evaporated under reduced pressure. The residue was diluted with EtOAc (40 ml) and washed with water (2x30 ml) and brine (2x30 ml).The organic layer was dried over $MgSO_4$, and concentrated. The crude product was purified by column chromatography (silica gel) using petroleum ether/ EtOAc = 4/1 as the elution solvent to afford compound **7.**

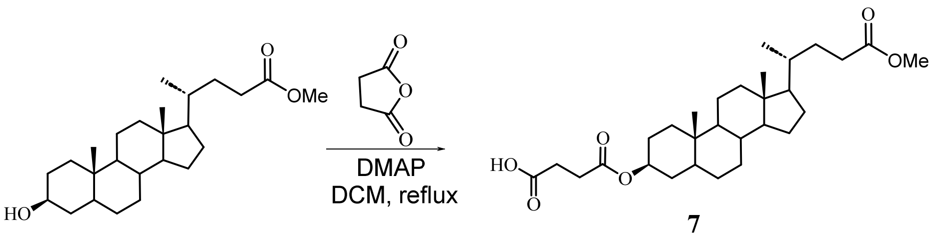

2.3.5. Procedure for synthesis compound **14.**

Derivative (**7**), respectively (0.24 mmol) were dissolved in 7 ml of DMF, then EDC*HCl (37.3 mg, 0.24 mmol) and DMAP (29.3 mg, 0.24 mmol) were successively added to the resulting solution at 0°C and stirred for 20 min. Then the reaction was brought to room temperature and CPT (41.80 mg, 0.12 mmol) was added and stirred at rt. under (Ar) atmosphere for 48 h. Upon completion, the resulting reaction mass was filtered and concentrated. The resulting crude product was purified by column chromatography on SiO_2 (EtOAc) to obtain conjugate **14**.

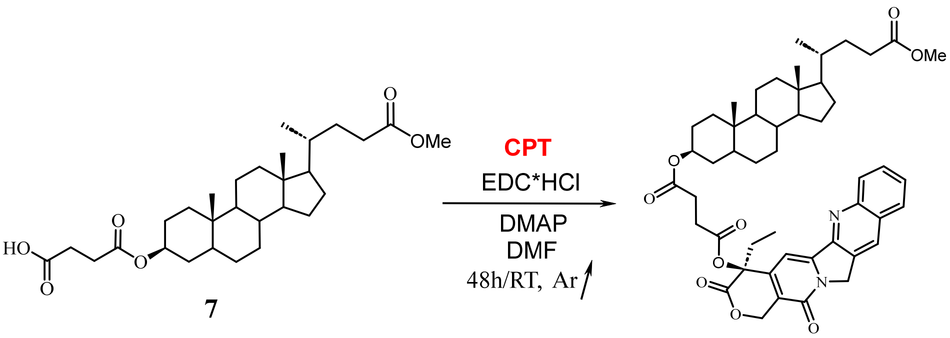

7 **14**

2.3.6. General procedure for the synthesis of 2-hydroxyethyl-(3β)-3(acetyloxy)ursane(olean)-12-ene-28-oate derivatives **18, 19.**

To a solution of (3β)-3-(acetyloxy)ursane(olean)-12-ene-28-oic acid (498 mg, 1 mmol) in dry CH_2Cl_2 (20 ml) at 0°C was added oxalyl chloride (1.70 ml, 18.0 mmol), and the mixture was stirred overnight at room temperature. The resulting reaction mixture was evaporated under reduced pressure diluted with dry hexane (3x10 ml), and re-evaporated. The residue was dissolved in dry CH_2Cl_2 (20 ml), and then DIPEA (0.52 ml, 3.00 mmol) and 1,2-ethanediol (124.1 mg, 2.00 mmol) were added at 0°C. After stirring at room temperature for 24 h, the solvent was evaporated.

The resulting crude product was purified by column chromatography on SiO_2using petroleum ether/ EtOAc = 1/1 as the elution solvent to afford derivatives **18** and **19**.

2.3.7. General procedure for the synthesis of derivatives **21, 22.**

To a stirred solution of 18 (19) (271. 4 mg, 0.5 mmol) and DMAP (74.03 mg, 0.6 mmol) in dry CH_2Cl_2 (15 ml) was added succinic anhydride (200.48 mg, 2.0 mmol) at room temperature. The resulting reaction was refluxed for 10 h. After completion of the reaction, the solvent was evaporated under reduced pressure. The residue was diluted with EtOAc (25 ml) and washed with water (2x20 ml) and saturated NaCl solution (2x20 ml). The organic layer was dried over $MgSO_4$ the solvent was evaporated.

The resulting crude product was purified by column chromatography on SiO_2 using petroleum ether/ EtOAc = 4/1 as the elution solvent to afford derivatives **21** and **22**.

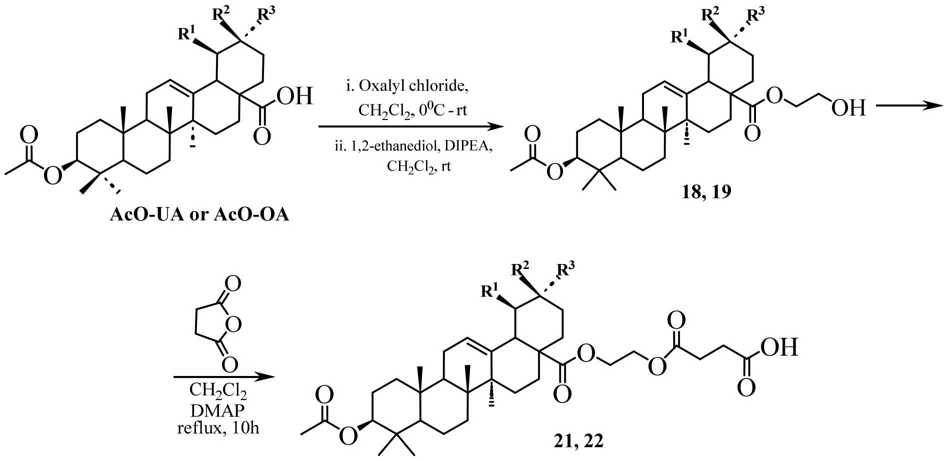

2.3.8. General procedure for the synthesis of derivatives **24** and **25.**

The hybrid molecules were obtained by the procedure from the corresponding derivatives 24 and 25 described above.

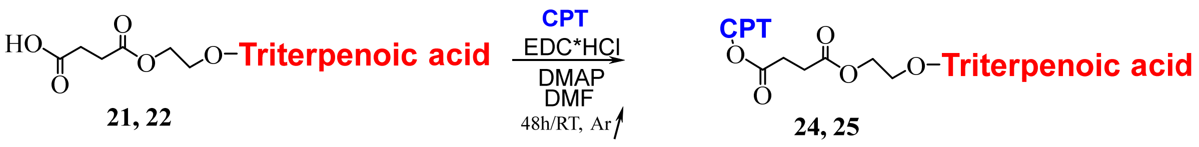

Hybrid molecules stuctures

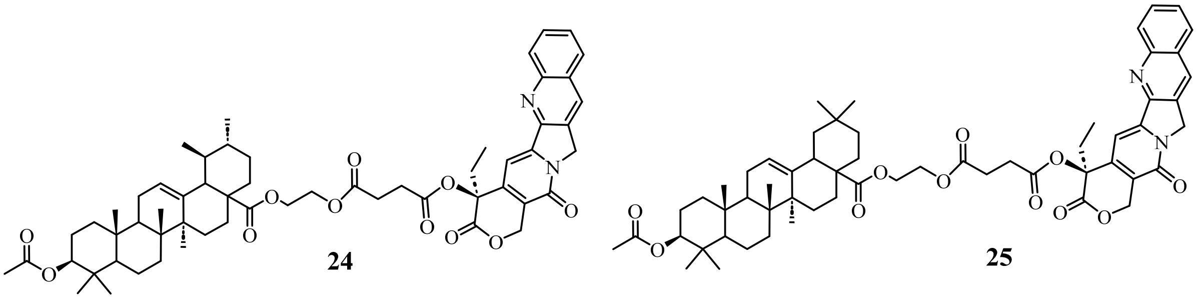

2.3.9. General procedure for the synthesis of betulonic acid.

Freshly prepared Johnson reagent (2.3 ml) was added drop wise to a solution of betulin (1 g, 2.26 mmol) in acetone (100 ml) at 0°C, and the mixture was stirred with cooling for 2 h. After the reaction, solution was quenched with methanol (25 ml), stirred for 10 min and then 50 ml H_2O was added. All solid impurities were filtrated off. After removing the solvent under reduced pressure, the aqueous layer was extracted with EtOAc (2x50 ml). The organic layer was washed with water and saturated NaCl solution, dried over $MgSO_4$, and the solvent was evaporated under reduced pressure.

The resulting crude product was purified by column chromatography on SiO_2 using petroleum ether/EtOAc = 4/1 as the elution solvent to afford 3-Oxo-20(29)-lupen-28-oic acid (**betulonic acid**).

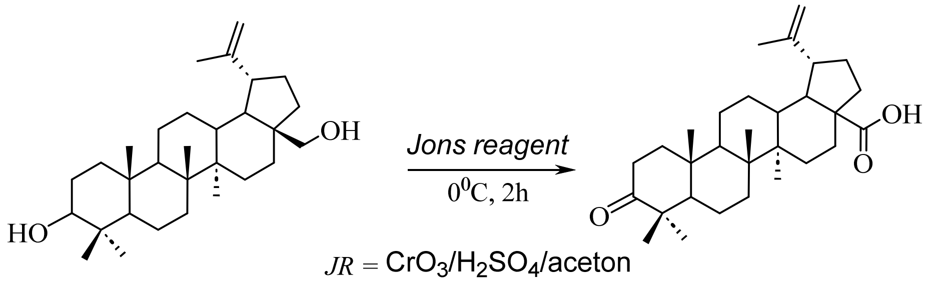

2.3.10. General procedure for the synthesis of methyl ester betulinic acid.

Betulonic acid was reduced with sodium borohydride ($NaBH_4$) in tetrahydrofuran (THF). The reaction mixture was quenched with 10% HCl solution. The solution was diluted with EtOAc (50 ml). The organic layer was washed with H_2O, brine. The resulting solution was dried over $MgSO_4$ and filtrated. The solvent was evaporated under reduced pressure. The resulting betulinic acid was used for further modification.

In the second step, K_2CO_3 (2.28 mmol, 303.24 mg) was added to a stirred solution of betulinic acid (260 mg, 0.57 mmol) in 10 ml DMF, followed by the CH_3I at 0°C. The reaction mixture was poured into 25 ml H_2O. The suspension was filtered through a Buchner funnel, the filter was washed several times with water. The resulting precipitate was dried under reduced pressure. The crude product was purified by column chromatography on SiO_2 using petroleum ether/ EtOAc = 4/1 as the elution solvent to afford betulinic acid methyl ester.

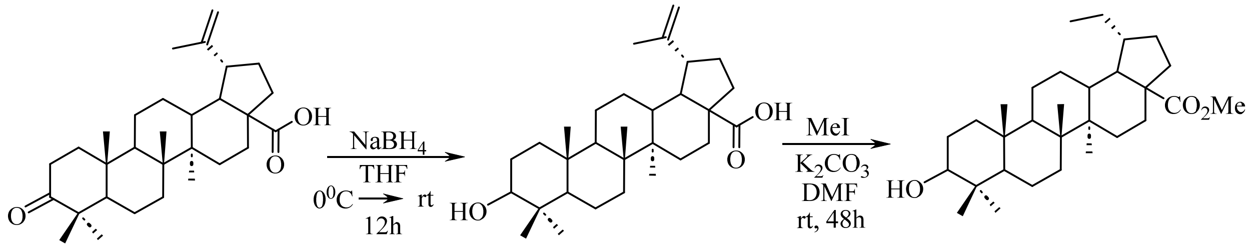

2.3.11. General procedure for the synthesis of methyl ester betulinic acid with succinic anhydride.

To a stirred solution of derivatives betulinicacid methyl ester (120 mg, 0.25 mmol) and DMAP (0.31 mmol) in dry CH_2Cl_2 (15 ml) was added excess succinic anhydride (1.00 mmol) at room temperature. The resulting reaction mixture was refluxed for 15 h. After completion of the reaction, the solvent was evaporated under reduced pressure. The residue was diluted with EtOAc (30 ml) and washed with water (2x25 ml) and brine (2x25 ml).The organic layer was dried over $MgSO_4$, and concentrated. The crude product was purified by column

chromatography (silica gel) using petroleum ether/ EtOAc = 1/1 as the elution solvent to afford betulinic acid O-hemisuccinate **3**.

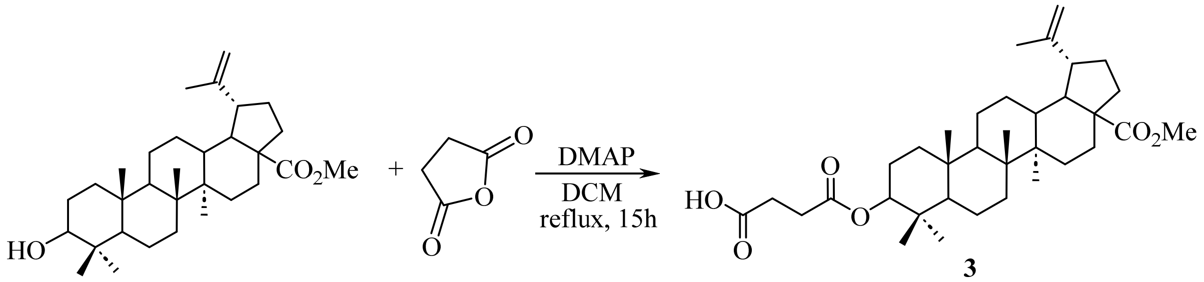

2.3.12. General procedure for the synthesis of hybrid molecule from betulinic acid O-hemisuccinate **3** *and CPT.*

Acid **3** (80 mg, 0.14 mmol) was dissolved in 5 ml of DMF, then EDC*HCl (21.73 mg, 0.14 mmol) and DMAP (17.1 mg, 0.14 mmol) were successively added to resulting solution at 0°C and stirred for 20 min. at 0°C. Then the reaction was brought to rt. and CPT (camptothecin) (24.40 mg, 0.07 mmol) was added and stirred at rt. under (Ar) atmosphere for 48 h. Upon completion, the resulting reaction mass was filtered and concentrated. The resulting crude product was purified by column chromatography on SiO_2 (EtOAc) to obtain conjugate **10**.

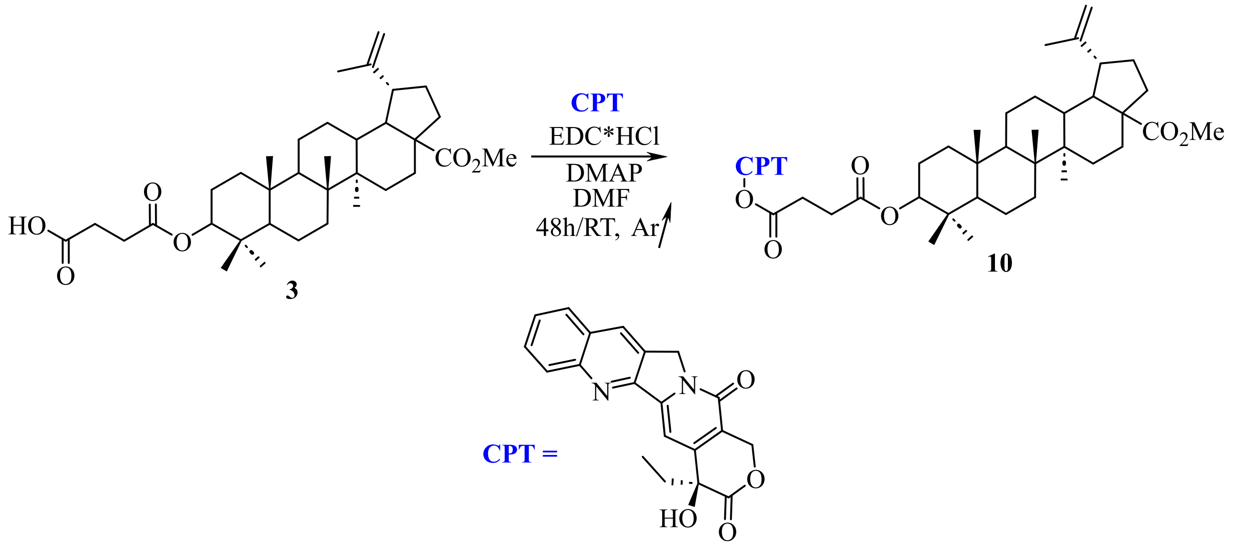

2.3.13. General procedure for the synthesis of betulonic acid derivative **20.**

To a solution of 3-Oxo-20(29)-lupen-28-oic acid (370 mg, 0.81 mmol) in anhydrous CH_2Cl_2 (20 ml) at 0°C, oxalyl chloride (0.858 ml, 10.0 mmol) was added. After stirring at room temperature overnight, the mixture was evaporated, and co-evaporated with dry hexane (3 × 10 ml). The residue was dissolved in dry CH_2Cl_2 (20 ml), and then DIPEA (0.52 ml, 3.00 mmol) and 1,2-ethanediol (124.1 mg, 2.0 mmol) were added at 0°C. After stirring at room temperature. for 24 h, the solvent was evaporated. The crude product was purified by column chromatography (silica gel) using petroleum ether/ EtOAc = 1/1 as the elution solvent to afford derivative **20**.

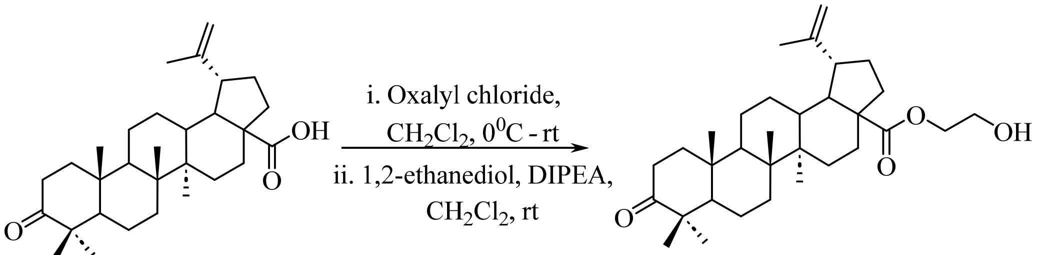

20

2.3.14. General procedure for the synthesis of betulonic derivative **23.**

To a stirred solution of 20 (200.0 mg, 0.4 mmol) and DMAP (74.03 mg, 0.6 mmol) in anhydrous CH_2Cl_2 (20 ml) was added succinic anhydride (200.14 mg, 2.0 mmol) at room temperature. The resulting reaction mixture was refluxed for 15 h. After completion of the reaction, the solvent was evaporated under reduced pressure. The residue was diluted with EtOAc (25 ml) and washed with water (2x20 ml) and saturated NaCl solution (2x30 ml). The organic layer was dried over $MgSO_4$ then evaporated under reduced pressure. The crude product was purified by column chromatography (silica gel) using petroleum ether/ EtOAc = 1/1 as the elution solvent to afford derivative **23**.

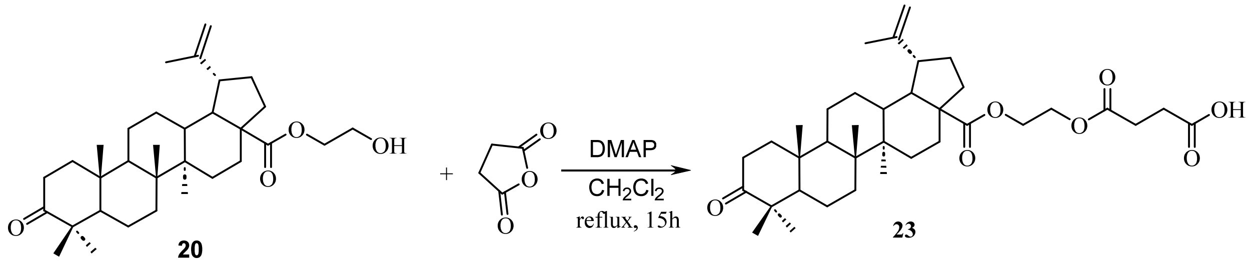

2.3.15. General procedure for the synthesis conjugates **(26)** *of from betulonic acid derivatives* **23** *andcamptothecin.*

Acid **23** (210 mg, 0.35 mmol) was dissolved in 5 ml of DMF, followed by *N*-[3-(methylamino)propyl]-*N'*-ethylcarbodiimide hydrochloride (EDC·HCl) (54.0 mg, 0.35 mmol) and DMAP (43.0 mg, 0.35 mmol) were successively added to resulting solution at 0°C and stirred for 20 min at 0°C. Then the reaction was brought to rt. and CPT (camptothecin) (61.0 mg, 0.175 mmol) was added and stirred at rt. under (Ar) atmosphere for 48 h. Upon completion, the resulting reaction mass was filtered and concentrated. The resulting crude product was purified by column chromatography on SiO_2 (EtOAc) to obtain conjugate **26**.

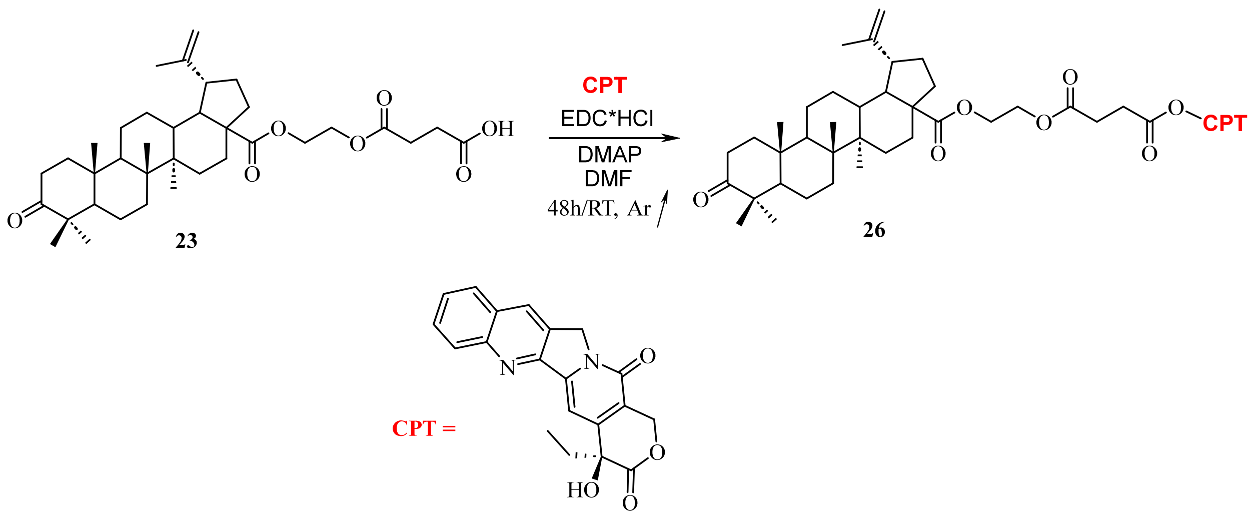

2.3.16. General procedure for the synthesis derivative **16**.

In the first stage, **preparation 28-O-succinyl betulin.**

BE (betulin) (2.5 mmol), succinic anhydride (2.6 mmol) and pyridine (5 mL) were heating refluxed in (50 ml) of anhydrous CH_2Cl_2 stirring in a round-bottom flask. The reaction was monitored by TLC. After betulin disappeared on TLC, the reactant was thoroughly washed with saturated Na_2CO_3 and NaCl solutions, and the mixture was dried over anhydrous $MgSO_4$ and subsequently filtered. After that, the solvent was removed under reduced pressure. The crude product was purified by column chromatography (silica gel) using petroleum ether/EtOAc = 2/1 as the elution solvent to afford derivative **15**. Then, oxidation with Jones reagent to obtain the starting acid **16**.

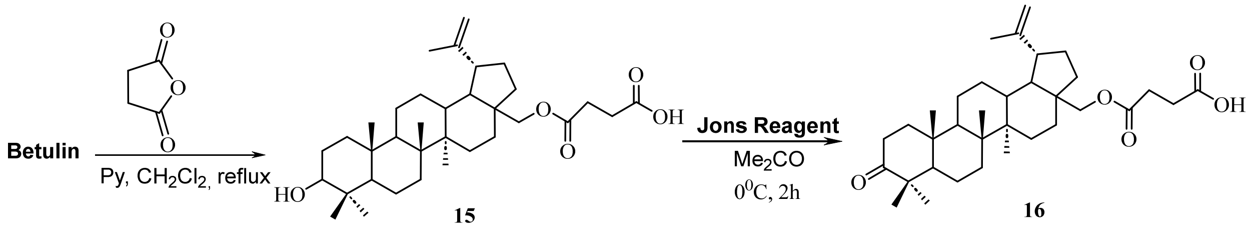

2.3.17. General procedure for the synthesis conjugates **(17)** *of from derivatives* **16** *and camptothecin.*

To a stirred solution of acid 16 (120 mg, 0.221 mmol) in 30 ml of anhydrous CH_2Cl_2, followed by *N*-[3-(methylamino)propyl]-*N*'-ethylcarbodiimide hydrochloride (EDC·HCl) (35.0 mg, 0.221 mmol) and 4-dimethylaminopyridine (DMAP) (27.0 mg, 0.221 mmol) were successively added to resulting solution at 0°C and stirred for 20 min at 0°C. Then the reaction was brought to rt. and CPT (camptothecin) (38.32 mg, 0.11 mmol) was added and stirred at rt. under (Ar) atmosphere for 24 h. Upon completion, the resulting reaction mass was filtered and concentrated. The resulting crude product was purified by column chromatography on SiO_2 (EtOAc) to obtain conjugate **17**.

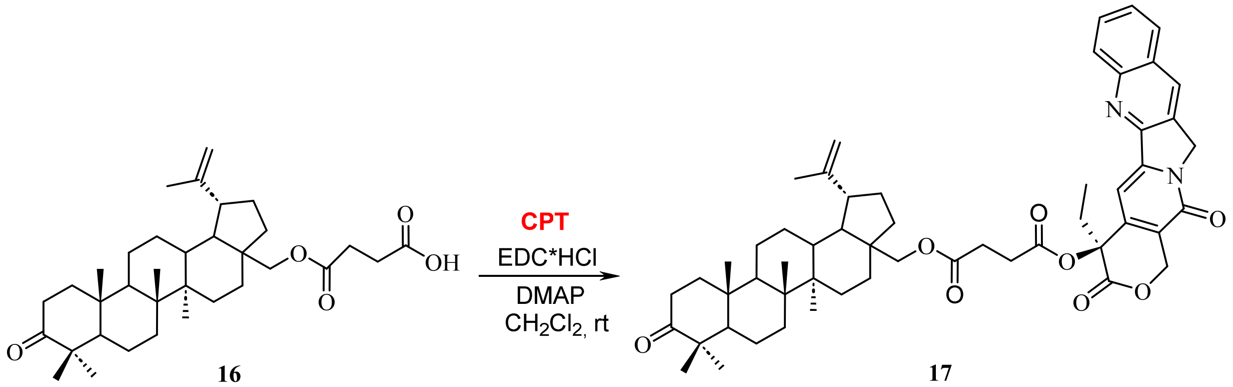

16 **17**

3. Characterization of all products.

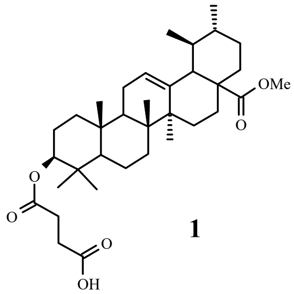

1

Methyl 3β-O-carboxypropionylurs-12-ene-28-oate.

White solid (524.76mg, 92% yield). ^{1}H NMR (300 MHz, Chloroform-*d*) δ 5.25 (t, *J* = 3.6Hz, 1H, H-12), 4.54 (dd, *J* = 9.7Hz, 7.6Hz, 1H, H-3), 3.62 (s, 3H, -OCH_3), 2.66 (dd, 4H, -$\underline{CH_2CH_2}$COOH), 2.24 (d, *J* = 11.1Hz, 1H, H-18), 1.09 (s, 3H), 0.95 (s, 3H), 0.87 (s, 3H), 0.76 (s, 3H).

^{13}C NMR (75 MHz, $CDCl_3$) δ 178.17, 177.75, 171.89, 138.24, 125.51, 81.59, 77.52, 77.09, 76.67, 55.37, 52.93, 51.52, 48.15, 47.52, 42.04, 39.56, 39.09, 38.93, 38.31, 37.78, 36.91, 36.69, 32.94, 30.70, 29.40, 29.09, 28.07, 24.28, 23.64, 23.53, 23.36, 21.24, 18.25, 17.12, 16.96, 16.80, 15.53.

ESI-MS: m/z: Calcd. for $C_{35}H_{54}O_6$ $[M-H]^-$: 569.3847; Found: 569.3848.

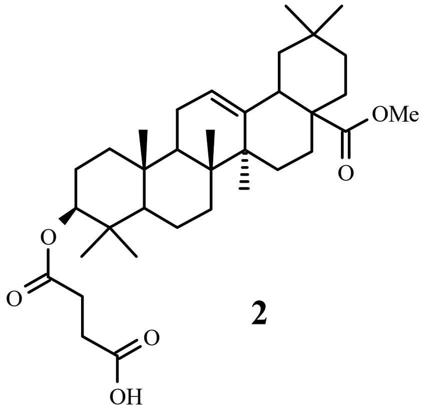

2

Methyl 3β-O-carboxypropionylolean-12-ene-28-oate.

White solid (541.87mg, 95% yield). ^{1}H NMR (300 MHz, Chloroform-*d*) δ 5.30 (t, *J* = 3.6 Hz, 1H, H-12), 4.54 (t, *J* = 7.9 Hz, 1H, H-3), 3.64 (s, 3H, -OCH_3), 2.94 – 2.81 (m, 1H, H-18), 2.75 - 2.58 (m, 4H, -$\underline{CH_2CH_2}$COOH), 1.15 (s, 3H), 0.94 (s, 3H), 0.92 (s, 3H), 0.87 (s, 3H), 0.74 (s, 3H).

^{13}C NMR (75Hz, $CDCl_3$): *δ* 178.41, 177.89, 171.89, 143.89, 122.35, 81.64, 55.42, 51.61, 47.64, 46.82, 45.94, 41.73, 41.39, 39.38, 38.18, 37.82, 37.01, 33.95, 33.18, 32.69, 32.47, 30.77, 29.42, 29.12, 28.06, 27.77, 25.99, 23.72, 23.53, 23.50, 23.16, 18.29, 16.92, 16.77, 15.42.

ESI-MS: m/z: Calcd. for $C_{35}H_{54}O_6$ $[M\text{-}H]^-$: 569.3847; Found: 569.3848.

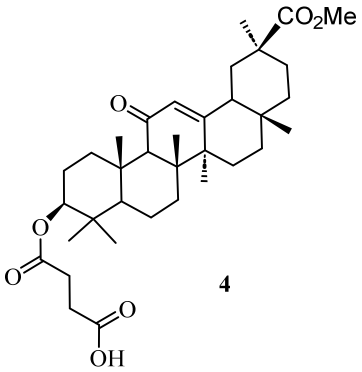

4

Methyl 3β-O-carboxypropionyl-11-oxoolean-12-ene-30-oate.

White solid (514.25mg, 88% yield). ^{1}H NMR (300 MHz, Chloroform-*d*) δ 5.70 (s, 1H, H-12), 4.58 (dd, *J* = 11.5, 4.9 Hz, 1H, H-3), 3.73 (s, 3H, OCH_3), 2.88 – 2.78 (m, 1H, H-18), 2.71 – 2.62 (m, 4H, -$\underline{CH_2CH_2}$COOH), 2.39 (s, 1H, H-9), 2.16 – 2.02 (m, 4H), 2.01 (s, 3H), 1.89 – 1.56 (m, 6H), 1.45 (s, 1H), 1.44 – 1.26 (m, 6H), 1.26 – 0.99 (m, 12H), 0.90 (s, 6H), 0.83 (s, 3H).

^{13}C NMR (75 MHz, $CDCl_3$) δ 200.24, 177.78, 177.05, 171.91, 169.46, 128.54, 81.30, 61.78, 55.12, 51.87, 48.49, 45.49, 44.12, 43.29, 41.15, 38.82, 38.18, 37.82, 37.01, 32.77, 31.91, 31.21, 29.42, 29.10, 28.60, 28.39, 28.06, 26.55, 26.49, 23.56, 23.42, 18.75, 17.44, 16.77, 16.46.

ESI-MS: m/z Calcd. for $C_{35}H_{52}O_7$ $[M\text{-}H]^-$: 583.3639; Found: 583.3640.

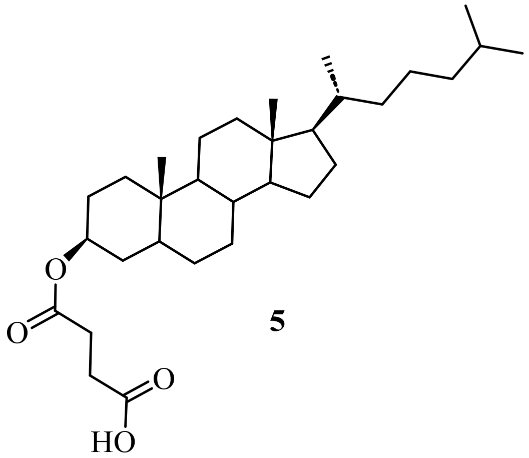

5

5α-Cholestan-3β-hemisuccinate.

White solid (464.1mg, 95% yield). ^{1}H NMR (300 MHz, Chloroform-*d*) δ 2.74 – 2.55 (m, 1H), 1.87 – 1.61 (m, 1H), 1.61 – 1.48 (m, 1H), 1.42 – 1.33 (m, 1H), 1.33 – 1.11 (m, 3H), 1.11 – 0.86 (m, 4H), 0.83 (s, 1H), 0.66 (s, 1H).

^{13}C NMR (75 MHz, $CDCl_3$) δ 178.14, 171.75, 74.45, 56.51, 56.37, 54.30, 44.74, 42.68, 40.08, 39.61, 36.81, 36.26, 35.89, 35.55, 34.01, 32.08, 29.36, 29.12, 28.68, 28.33, 28.09, 27.48, 24.30, 23.94, 22.90, 22.65, 21.30, 18.76, 12.31, 12.16.

ESI-MS: m/z Calcd. for $C_{31}H_{52}O_4$ $[M+H]^+$: 489.3938; Found: 489.3940.

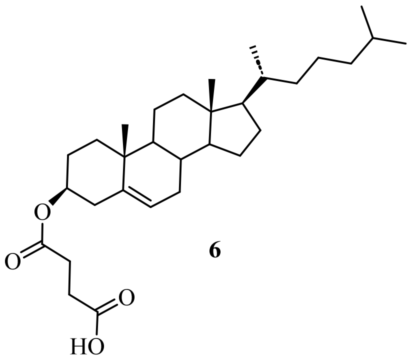

6

Cholesteryl hemisuccinate.

White solid (447.5mg, 92% yield). ^{1}H NMR (300 MHz, Chloroform-*d*) δ 5.38 (d, *J* = 5.0 Hz, 2H), 4.72 – 4.55 (m, 2H), 2.64 (dd, *J* = 16.1, 5.9 Hz, 6H), 2.32 (d, *J* = 8.1 Hz, 4H), 1.02 (s, 6H), 0.92 (d, *J* = 6.5 Hz, 5H), 0.87 (d, *J* = 6.6 Hz, 8H), 0.68 (s, 6H).

^{13}C NMR (75 MHz, $CDCl_3$) δ 178.05, 171.61, 139.62, 122.83, 74.64, 56.78, 56.24, 50.10, 42.40, 39.82, 39.61, 38.10, 37.04, 36.67, 36.28, 35.89, 31.99, 31.94, 29.32, 29.09, 28.32, 28.10, 27.78, 24.38, 23.93, 22.91, 22.65, 21.12, 19.39, 18.81, 11.95.

ESI-MS: m/z Calcd. for $C_{31}H_{50}O_4$ $[M+H]^+$: 487.3781; Found: 487.3782.

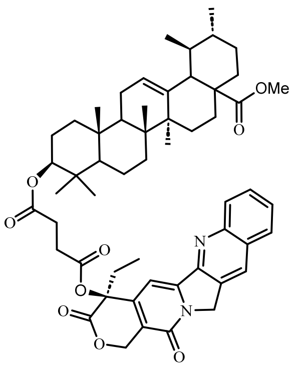

8

(*S*)-4-ethyl-3,14-dioxo-3,4,12,14-tetrahydro-1*H*-pyrano[3’,4’:6,7]indolizino[1,2-*b*]quinolin-4-yl (methyl 3β-*O*-carboxypropionyl)urs-12-en-28-oate.

Light yellow solid (168.1mg, 76% yield). ^{1}H NMR (300 MHz, Chloroform-*d*) δ 8.43 (s, 1H), 8.30 (d, *J* = 8.6 Hz, 1H), 7.95 (d, *J* = 8.1 Hz, 1H), 7.85 (t, *J* = 7.7 Hz, 1H), 7.67 (t, *J* = 7.6 Hz,

1H), 7.39 (s, 1H), 5.67 (d, J = 17.2 Hz, 1H), 5.39 (d, J = 17.3 Hz, 1H), 5.29 (s, 2H), 5.23 (d, J = 3.7 Hz, 1H), 4.38 (dd, J = 10.3, 5.7 Hz, 1H), 3.60 (s, 3H), 2.95 – 2.77 (m, 2H), 2.70 – 2.56 (m, 2H), 2.35 – 2.08 (m, 3H), 2.06 – 1.88 (m, 1H), 1.88 – 1.13 (m, 18H), 1.07 – 0.92 (m, 10H), 0.88 (d, J = 6.4 Hz, 3H), 0.80 (d, J = 7.1 Hz, 6H), 0.70 (d, J = 10.2 Hz, 6H), 0.62 (d, J = 10.5 Hz, 1H).

^{13}C NMR (75 MHz, $CDCl_3$) δ 178.06, 171.56, 171.40, 167.38, 157.32, 151.99, 148.22, 145.97, 145.62, 138.15, 131.71, 130.97, 129.22, 128.59, 128.23, 128.18, 125.45, 120.45, 97.10, 81.51, 76.13, 67.01, 55.24, 52.87, 51.45, 49.97, 48.08, 47.38, 41.95, 39.43, 39.06, 38.88, 38.14, 37.63, 36.72, 36.64, 32.81, 31.76, 30.65, 29.22, 29.02, 28.05, 28.00, 24.21, 23.64, 23.40, 23.24, 21.20, 18.10, 17.07, 16.83, 16.75, 15.33, 7.62.

ESI-MS: m/z Calcd. for $C_{55}H_{68}N_2O_9$ $[M+Na]^+$: 923.4817; Found: 923.4817.

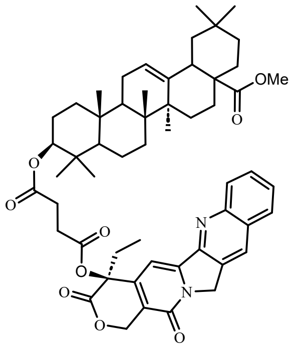

9

(*S*)-4-ethyl-3,14-dioxo-3,4,12,14-tetrahydro-1*H*-pyrano[3',4':6,7]indolizino[1,2-*b*]quinolin-4-yl (methyl 3β-*O*-carboxypropionyl)olean-12-en-28-oate.

Light yellow solid (175.5mg, 78% yield). ^{1}H NMR (300 MHz, Chloroform-*d*) δ 8.40 (s, 1H), 8.26 (d, J = 8.5 Hz, 1H), 7.94 (d, J = 8.1 Hz, 1H), 7.84 (t, J = 7.7 Hz, 1H), 7.67 (t, J = 7.5 Hz, 1H), 7.30 (s, 1H), 5.67 (d, J = 17.3 Hz, 1H), 5.41 (d, J = 17.2 Hz, 1H), 5.28 (s, 2H), 4.42 (t, J = 8.0 Hz, 1H), 3.63 (s, 3H), 2.92 – 2.80 (m, 3H), 2.64 (q, J = 6.3 Hz, 2H), 2.30 (dd, J = 14.0, 7.3 Hz, 1H), 2.23 – 2.10 (m, 1H), 2.05 – 1.89 (m, 1H), 1.79 (d, J = 10.1 Hz, 2H), 1.71 – 0.53 (m, 44H).

^{13}C NMR (75 MHz, $CDCl_3$) δ 178.29, 171.49, 171.38, 167.43, 157.37, 152.31, 148.76, 146.10, 145.83, 143.81, 131.27, 130.74, 129.63, 128.51, 128.22, 128.18, 128.09, 122.27, 120.36, 96.48, 81.46, 76.16, 67.10, 55.27, 51.55, 49.96, 47.48, 46.74, 45.93, 41.63, 41.32, 39.24, 38.00, 37.68, 36.82, 33.89, 33.15, 32.54, 32.40, 31.85, 30.73, 29.27, 29.07, 28.03, 27.70, 25.97, 23.68, 23.42, 23.37, 23.09, 18.14, 16.79, 16.73, 15.22, 7.63.

ESI-MS: m/z Calcd. for $C_{55}H_{68}N_2O_9$ $[M+Na]^+$: 923.4817; Found: 923.4817.

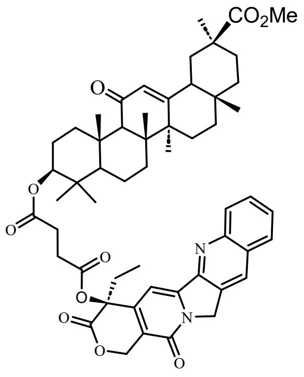

11

(*S*)-4-ethyl-3,14-dioxo-3,4,12,14-tetrahydro-1*H*-pyrano[3',4':6,7]indolizino[1,2-*b*]quinolin-4-yl (methyl 3β-*O*-carboxypropionyl)-11-oxoolean-12-en-30-oate.

Yellow solid (175.8mg, 80% yield). ^{1}H NMR (300 MHz, Chloroform-*d*) δ 8.44 (s, 1H), 8.28 (d, *J* = 8.5 Hz, 1H), 7.95 (d, *J* = 8.1 Hz, 1H), 7.85 (t, *J* = 7.7 Hz, 1H), 7.67 (t, *J* = 7.6 Hz, 1H), 7.35 (s, 1H), 5.66 (d, *J* = 2.8 Hz, 2H), 5.44 (s, 1H), 5.30 (s, 2H), 4.47 (dd, *J* = 10.8, 5.5 Hz, 1H), 3.71 (s, 3H), 2.96 – 2.80 (m, 3H), 2.75 – 2.55 (m, 3H), 2.40 – 2.16 (m, 3H), 2.16 – 2.04 (m, 1H), 2.04 – 1.95 (m, 3H), 1.95 – 1.73 (m, 1H), 1.64 (d, *J* = 13.5 Hz, 2H), 1.60 – 1.54 (m, 6H), 1.51 - 1.28 (m, 8H), 1.17 (s, 3H), 1.10 – 1.03 (m, 3H), 1.02 (s, 1H), 0.98 (d, *J* = 7.6 Hz, 5H), 0.86 – 0.74 (m, 8H), 0.66 (d, *J* = 11.1 Hz, 1H).

^{13}C NMR (75 MHz, $CDCl_3$) δ 200.04, 176.98, 171.47, 171.38, 169.33, 167.45, 157.32, 152.17, 148.52, 145.96, 145.75, 131.56, 130.84, 129.45, 128.56, 128.45, 128.23, 128.19, 128.10, 120.43, 96.61, 81.11, 76.12, 67.10, 61.61, 54.94, 51.84, 49.99, 48.39, 45.34, 44.06, 43.15, 41.10, 38.69, 38.03, 37.75, 36.79, 32.60, 31.84, 31.13, 29.27, 29.02, 28.54, 28.37, 28.01, 26.44, 26.39, 23.40, 18.60, 17.27, 16.73, 16.23, 7.64.

ESI-MS: m/z Calcd. for $C_{55}H_{66}N_2O_{10}$ $[M+H]^+$: 915.4790; Found: 915.4790.

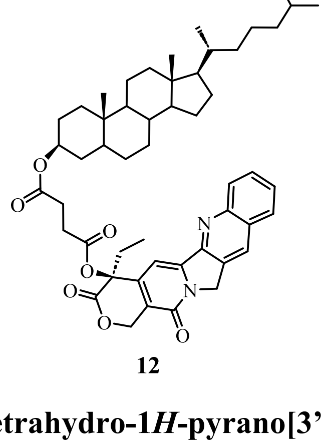

12

(*S*)-4-ethyl-3,14-dioxo-3,4,12,14-tetrahydro-1*H*-pyrano[3',4':6,7]indolizino[1,2-*b*]quinolin-4-yl-cholestan-3β-hemisuccinate.

White solid (180.9 mg, 92% yield). ^{1}H NMR (300 MHz, Chloroform-*d*) δ 8.42 (s, 1H), 8.30 (d, *J* = 8.4 Hz, 1H), 7.96 (d, *J* = 8.0 Hz, 1H), 7.85 (t, *J* = 7.8 Hz, 1H), 7.69 (t, *J* = 7.7 Hz, 1H), 7.40

(s, 1H), 5.69 (d, J = 17.2 Hz, 1H), 5.40 (d, J = 17.3 Hz, 1H), 5.28 (s, 2H), 4.61 (s, 1H), 0.64 (d, J = 18.4 Hz, 6H).

^{13}C NMR (75 MHz, $CDCl_3$) δ 171.56, 171.37, 167.45, 157.40, 152.25, 148.60, 146.14, 145.83, 131.45, 130.87, 129.60, 128.59, 128.28, 128.22, 128.16, 120.22, 97.03, 76.22, 74.26, 67.01, 56.46, 56.35, 54.01, 49.98, 44.36, 42.59, 40.02, 39.55, 36.57, 36.21, 35.82, 35.32, 35.25, 33.80, 31.77, 29.26, 29.01, 28.42, 28.26, 28.05, 27.32, 24.20, 23.90, 22.86, 22.60, 21.11, 18.72, 12.05, 7.67.

ESI-MS: m/z Calcd. for $C_{51}H_{66}N_2O_7$ $[M+H]^+$: 819.4942; Found: 819.4943.

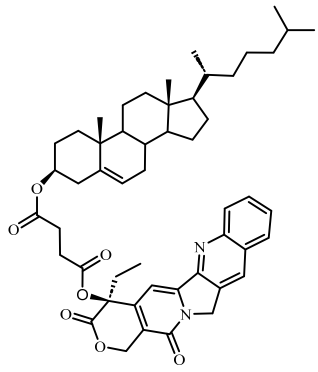

13

(*S*)-4-ethyl-3,14-dioxo-3,4,12,14-tetrahydro-1*H*-pyrano[3',4':6,7]indolizino[1,2-*b*]quinolin-4-yl-cholesteryl hemisuccinate.

White solid (174.4mg, 89% yield). ^{1}H NMR (300 MHz, Chloroform-*d*) δ 8.38 (s, 1H), 8.24 (d, J = 8.6 Hz, 1H), 7.93 (d, J = 8.2 Hz, 1H), 7.82 (t, J = 7.7 Hz, 1H), 7.66 (t, J = 7.5 Hz, 1H), 7.32 (s, 1H), 5.68 (d, J = 17.3 Hz, 1H), 5.39 (d, J = 17.1 Hz, 1H), 5.27 (s, 2H), 5.04 (d, J = 4.9 Hz, 1H), 4.52 (dd, J = 11.2, 5.9 Hz, 1H), 2.93 – 2.73 (m, 2H), 2.61 (q, J = 6.2 Hz, 2H), 2.28 (dd, J = 14.3, 7.4 Hz, 1H), 2.14 (dt, J = 14.8, 7.5 Hz, 3H), 1.97 (d, J = 12.4 Hz, 1H), 1.89 – 1.72 (m, 3H), 1.65 (d, J = 13.3 Hz, 1H), 1.58 – 1.45 (m, 3H), 1.30 (dq, J = 16.0, 8.3, 7.3 Hz, 9H), 1.19 - 1.04 (m, 8H), 0.99 (t, J = 7.6 Hz, 4H), 0.88 – 0.84 (m, 12H), 0.63 (s, 3H).

^{13}C NMR (75 MHz, $CDCl_3$) δ 171.48, 171.23, 167.48, 157.43, 152.38, 148.86, 146.11, 146.02, 139.52, 131.22, 130.73, 129.80, 128.55, 128.22, 128.07, 122.36, 120.20, 96.65, 76.22, 74.50, 67.10, 56.73, 56.22, 49.99, 49.84, 42.33, 39.77, 39.57, 37.90, 36.81, 36.44, 36.24, 35.84, 31.85, 31.80, 31.76, 29.24, 29.03, 28.27, 28.07, 27.65, 24.31, 23.90, 22.89, 22.63, 20.96, 19.19, 18.78, 11.86, 7.67.

ESI-MS: m/z Calcd. for $C_{51}H_{64}N_2O_7$ $[M+Na]^+$: 839.4606; Found: 839.4606.

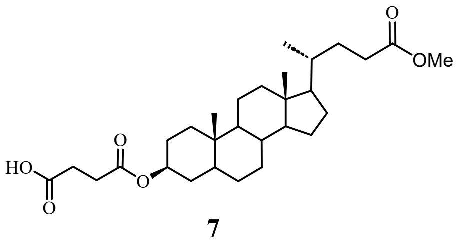

7

3α-(hydroxysuccinyl)oxy-5β-cholan-24-oate.

White solid (441.6mg, 90% yield). ^{1}H NMR (300 MHz, Chloroform-*d*) δ 3.60 (s, 1H), 2.66 - 2.47 (m, 2H), 2.37 – 2.07 (m, 1H), 1.96 – 1.55 (m, 8H), 1.33 (s, 3H), 1.24 – 1.10 (m, 1H), 1.10 – 0.96 (m, 1H), 0.96 – 0.81 (m, 1H), 0.58 (s, 1H).

^{13}C NMR (75 MHz, Chloroform-*d*) δ 177.75, 174.92, 171.75, 75.06, 56.53, 56.05, 51.57, 42.81, 41.97, 40.49, 40.19, 35.86, 35.43, 35.07, 34.65, 32.23, 31.14, 31.08, 29.34, 29.04, 28.44, 28.25, 27.08, 26.64, 26.38, 24.25, 23.38, 20.91, 18.34, 12.10.

ESI-MS: m/z Calcd. for $C_{29}H_{46}O_6$ [M+H]$^+$: 491.3367; Found: 491.3368.

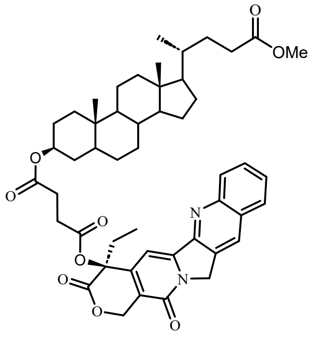

14

(*S*)-4-ethyl-3,14-dioxo-3,4,12,14-tetrahydro-1*H*-pyrano[3’,4’:6,7]indolizino[1,2-*b*]quinolin-4-yl-3α-(hydroxysuccinyl)oxy-5β-cholan-24-oate.

Light yellow solid (167.4mg, 85% yield). ^{1}H NMR (300 MHz, Chloroform-*d*) δ 8.40 (s, 1H), 8.27 (d, *J* = 8.5 Hz, 1H), 7.95 (d, *J* = 8.2 Hz, 1H), 7.84 (t, *J* = 7.7 Hz, 1H), 7.68 (t, *J* = 7.5 Hz, 1H), 7.34 (s, 1H), 5.69 (d, *J* = 17.2 Hz, 1H), 5.40 (d, *J* = 17.3 Hz, 1H), 5.28 (s, 1H), 4.75 – 4.54 (m, 2H), 3.67 (s, 3H), 2.97 – 2.74 (m, 2H), 2.73 – 2.53 (m, 2H), 2.45 – 2.03 (m, 2H), 2.00 – 0.95 (m, 30H), 0.90 (d, *J* = 6.1 Hz, 3H), 0.79 (s, 3H), 0.68 (d, *J* = 13.8 Hz, 1H), 0.62 (s, 3H).

^{13}C NMR (75 MHz, $CDCl_3$) δ 174.80, 171.51, 171.41, 167.43, 157.41, 152.39, 148.83, 146.09, 131.21, 130.68, 129.71, 128.52, 128.22, 128.18, 128.05, 120.08, 96.63, 76.24, 74.96, 67.02, 56.41, 55.97, 51.50, 49.95, 42.72, 41.81, 40.31, 40.07, 35.77, 35.37, 34.81, 34.44, 32.13, 31.76, 31.08, 31.03, 29.25, 29.02, 28.19, 27.02, 26.45, 26.29, 24.18, 23.20, 20.79, 18.29, 12.04, 7.66.

ESI-MS: m/z Calcd. for $C_{49}H_{60}N_2O_9$[M+Na]$^+$: 843.4191; Found: 843.4191.

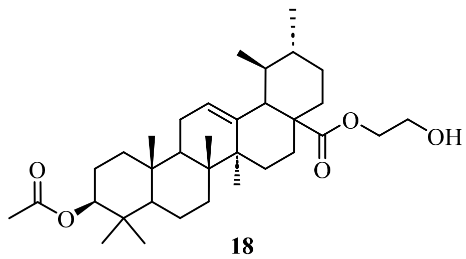

18

2-hydroxyethyl(3*β*)-3-(acetyloxy)urs-12-en-28-oate.

White solid (423.4mg, 78% yield). ^{1}H NMR (300 MHz, Chloroform-*d*) δ 5.27 (t, 1H), 4.51 (t, *J* = 7.9 Hz, 1H), 4.27 – 4.01 (m, 2H), 3.85 – 3.74 (m, 2H), 2.26 (d, *J* = 11.2 Hz, 1H), 2.04 (d,

J = 10.1 Hz, 6H), 2.00 – 1.17 (m, 16H), 1.10 (s, 6H), 0.96 (d, J = 5.3 Hz, 6H), 0.91 – 0.83 (m, 10H), 0.78 (s, 3H).

^{13}C NMR (75 MHz, $CDCl_3$) δ 177.91, 171.06, 139.01, 125.37, 80.97, 66.05, 61.40, 55.37, 53.08, 48.41, 47.56, 42.26, 39.65, 39.17, 38.95, 38.39, 37.75, 36.93, 36.80, 33.03, 30.69, 28.15, 28.02, 24.30, 23.62, 23.58, 23.39, 21.35, 21.21, 18.25, 17.19, 17.09, 16.80, 15.57.

ESI-MS: m/z Calcd. for $C_{35}H_{54}O_5$[M+H]$^+$:543.4044; Found: 543.4044.

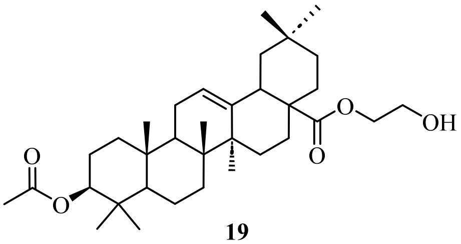

19

2-hydroxyethyl(3*β*)-3-(acetyloxy)olean-12-en-28-oate.

White solid (434.2mg, 80% yield). ^{1}H NMR (300 MHz, Chloroform-*d*) δ 5.31 (t, J = 3.8 Hz, 1H), 4.50 (t, J = 7.9 Hz, 1H), 4.27 – 4.08 (m, 6H), 3.80 (t, J = 4.7 Hz, 3H), 2.89 (dd, J = 13.5, 4.5 Hz, 1H), 2.05 (s, 3H), 2.02 – 0.99 (m, 20H), 0.97 – 0.90 (m, 10H), 0.87 (d, J = 3.5 Hz, 6H), 0.76 (s, 3H).

^{13}C NMR (75 MHz, $CDCl_3$) δ 178.19, 171.06, 144.19, 122.36, 80.99, 77.53, 77.10, 76.68, 66.11, 61.50, 55.38, 47.61, 47.02, 45.85, 41.88, 41.58, 39.44, 38.23, 37.76, 37.00, 33.91, 33.13, 32.76, 32.56, 30.76, 28.12, 27.72, 25.91, 23.69, 23.60, 23.50, 23.13, 21.34, 18.28, 17.10, 16.75, 15.44.

ESI-MS: m/z Calcd. for $C_{35}H_{54}O_5$[M+H]$^+$:543.4044; Found: 543.4044.

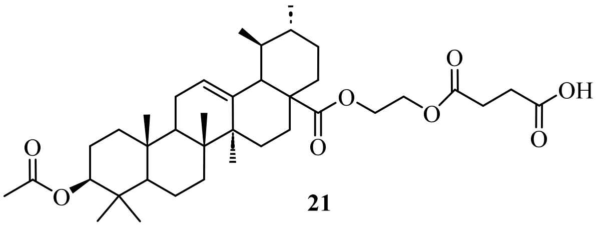

21

(3*β*)-3-(acetyloxy)urs-12-en-28-carbonyloxyethyl)succinic acid.

White solid (250.7mg, 78% yield). ^{1}H NMR (300 MHz, Chloroform-*d*) δ 5.24 (t, 1H), 4.49 (t, J = 7.9 Hz, 1H), 4.33 – 4.15 (m, 4H), 2.73 – 2.56 (m, 4H), 2.22 (d, J = 11.2 Hz, 1H), 2.01 – 1.18 (m, 21H), 1.07 (s, 3H), 1.05 – 0.90 (m, 6H), 0.90 – 0.79 (m, 14H), 0.75 (s, 3H).

^{13}C NMR (75 MHz, $CDCl_3$) δ 178.02, 177.31, 172.22, 171.16, 138.07, 125.61, 81.00, 62.65, 61.79, 55.31, 52.84, 48.17, 47.49, 42.05, 39.58, 39.05, 38.86, 38.33, 37.69, 36.87, 36.63, 33.00, 30.65, 29.71, 29.16, 28.89, 28.09, 27.98, 24.16, 23.52, 23.34, 21.29, 21.18, 18.21, 17.05, 16.76, 15.53.

ESI-MS: m/z Calcd. for $C_{38}H_{58}O_8$[M+Na]$^+$: 665.4023; Found: 665.4024.

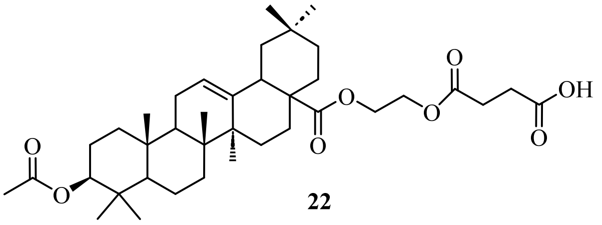

22

(3*β*)-3-(acetyloxy)olean-12-en-28-carbonyloxyethyl)succinic acid.

White solid (257.1mg, 80% yield). ^{1}H NMR (300 MHz, Chloroform-*d*) δ 5.30 (d, *J* = 3.7 Hz, 1H), 4.51 (t, *J* = 7.9 Hz, 1H), 4.37 – 4.09 (m, 4H), 2.87 (dd, *J* = 13.8, 4.4 Hz, 1H), 2.74 – 2.62 (m, 4H), 2.06 (s, 3H), 2.01 – 0.96 (m, 27H), 0.93 (d, *J* = 6.5 Hz, 8H), 0.87 (d, *J* = 3.3 Hz, 6H), 0.74 (s, 3H).

^{13}C NMR (75 MHz, $CDCl_3$) δ 177.56, 171.85, 171.19, 143.66, 122.49, 81.04, 62.63, 61.87, 55.36, 47.59, 46.84, 45.85, 41.75, 41.33, 39.40, 38.19, 37.75, 36.98, 33.90, 33.12, 32.75, 32.43, 30.73, 28.84, 28.77, 28.10, 27.68, 25.87, 23.63, 23.58, 23.48, 23.03, 21.35, 18.26, 17.00, 16.74, 15.43.

ESI-MS: m/z Calcd. for $C_{38}H_{58}O_8[M+Na]^+$: 665.4023; Found: 665.4024.

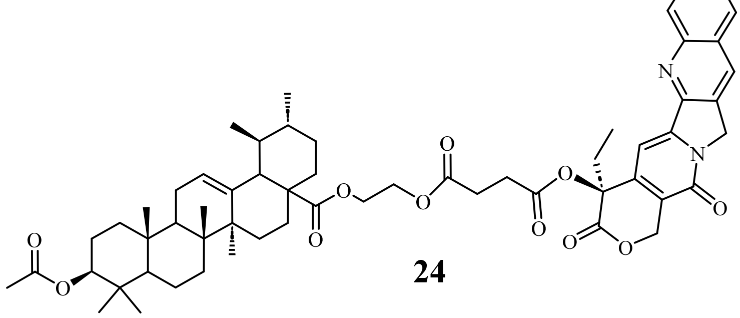

24

(*S*)-4-ethyl-3,14-dioxo-3,4,12,14-tetrahydro-1*H*-pyrano[3',4':6,7]indolizino[1,2-*b*]quinolin-4-(3*β*)-3-(acetyloxy)urs-12-en-28-carbonyloxyethyl)hemisuccinate.

Yellow solid (350.1mg, 72% yield). ^{1}H NMR (300 MHz, Chloroform-*d*) δ 8.42 (s, 1H), 8.25 (d, *J* = 8.6 Hz, 1H), 7.96 (d, *J* = 8.1 Hz, 1H), 7.85 (t, *J* = 7.7 Hz, 1H), 7.69 (t, *J* = 7.6 Hz, 1H), 7.33 (s, 1H), 5.69 (d, *J* = 17.3 Hz, 1H), 5.40 (d, *J* = 17.3 Hz, 1H), 5.30 (s, 2H), 5.22 (d, *J* = 3.5 Hz, 1H), 4.48 (t, *J* = 7.9 Hz, 1H), 4.36 – 4.00 (m, 6H), 2.97 – 2.78 (m, 2H), 2.68 (t, *J* = 7.5 Hz, 2H), 2.40 – 2.09 (m, 2H), 2.05 (s, 3H), 1.82 – 0.87 (m, 25H), 0.93 (d, *J* = 6.7 Hz, 8H), 0.85 (d, *J* = 4.6 Hz, 10H), 0.70 (s, 3H).

^{13}C NMR (75 MHz, $CDCl_3$) δ 177.27, 171.55, 171.24, 171.04, 167.34, 157.39, 152.31, 148.65, 146.03, 138.12, 131.45, 130.87, 129.45, 128.62, 128.30, 128.25, 128.16, 125.59, 120.24, 96.54, 80.97, 76.35, 67.05, 62.69, 61.81, 55.35, 52.85, 50.01, 48.16, 47.51, 42.06, 39.59, 39.07, 38.87, 38.33, 37.72, 36.89, 36.64, 33.00, 31.83, 30.65, 29.74, 28.95, 28.81, 28.12, 27.98, 24.17, 23.60, 23.54, 23.35, 21.36, 21.19, 18.23, 17.07, 17.04, 16.78, 15.55, 7.68.

ESI-MS: m/z Calcd. for $C_{58}H_{72}N_2O_{11}$ $[M+Na]^+$: 995.50288; Found: 995.5028.

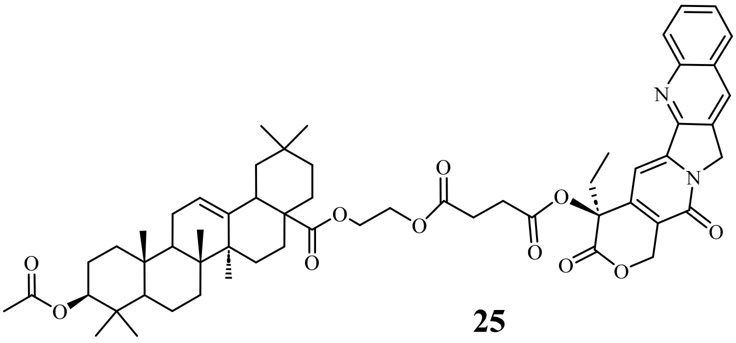

25

(*S*)-4-ethyl-3,14-dioxo-3,4,12,14-tetrahydro-1*H*-pyrano[3',4':6,7]indolizino[1,2-*b*]quinolin-4-(3*β*)-3-(acetyloxy)olean-12-en-28-carbonyloxyethyl)hemisuccinate.

Yellow solid (340.4mg, 70% yield). ^{1}H NMR (300 MHz, Chloroform-*d*) δ 8.42 (s, 1H), 8.25 (d, *J* = 8.5 Hz, 1H), 7.96 (d, *J* = 8.2 Hz, 1H), 7.85 (t, *J* = 7.7 Hz, 1H), 7.68 (t, *J* = 7.5 Hz, 1H), 7.31 (s, 1H), 5.69 (d, *J* = 17.2 Hz, 1H), 5.40 (d, *J* = 17.2 Hz, 1H), 5.31 – 5.21 (m, 3H), 4.48 (t, *J* = 7.9 Hz, 1H), 4.36 – 4.04 (m, 5H), 2.94 – 2.78 (m, 3H), 2.73 – 2.61 (m, 2H), 2.36 – 2.09 (m, 2H), 1.97 – 1.77 (m, 3H), 1.69 – 1.32 (m, 9H), 1.31 – 1.21 (m, 3H), 1.11 (s, 3H), 1.01 (t, *J* = 7.4 Hz, 3H), 0.95 – 0.80 (m, 15H), 0.69 (s, 3H).

^{13}C NMR (75 MHz, $CDCl_3$) δ 177.48, 171.52, 171.21, 171.04, 167.60, 157.38, 152.33, 148.68, 146.10, 146.01, 143.65, 131.40, 130.83, 129.48, 128.61, 128.29, 128.23, 128.14, 122.42, 120.19, 96.47, 80.96, 77.53, 77.10, 76.68, 76.34, 67.04, 62.65, 61.86, 55.34, 50.00, 47.55, 46.78, 45.83, 41.70, 41.28, 39.35, 38.14, 37.72, 36.94, 33.85, 33.09, 32.69, 32.38, 31.82, 30.70, 28.92, 28.78, 28.07, 27.63, 25.84, 23.62, 23.56, 23.44, 22.98, 21.35, 18.23, 16.93, 16.71, 15.39, 7.66.

ESI-MS: m/z Calcd. for $C_{58}H_{72}N_2O_{11}$ $[M+Na]^+$: 995.50288; Found: 995.5028.

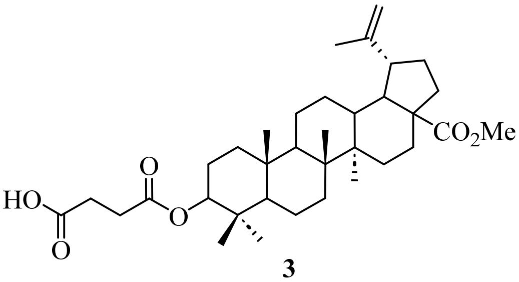

3

Methyl (3β-O-carboxypropionyl)lup20(29)-en-28-oate.

White solid (137.0 mg, 96% yield). ^{1}H NMR (300 MHz, Chloroform-*d*) δ 4.75 (d, *J* = 2.4 Hz, 1H), 4.62 (s, 1H), 4.52 (t, *J* = 8.0 Hz, 1H), 3.69 (s, 3H), 3.11 – 2.92 (m, 1H), 2.76 – 2.56 (m, 4H), 2.29 – 2.15 (m, 2H), 1.98 – 1.55 (m, 10H), 1.54 – 1.02 (m, 14H), 0.96 (d, *J* = 13.7 Hz, 7H), 0.90 – 0.74 (m, 10H).

^{13}C NMR (75 MHz, $CDCl_3$) δ 177.87, 176.77, 171.90, 150.62, 109.71, 81.67, 56.66, 55.55, 51.33, 50.55, 49.57, 47.08, 42.49, 40.79, 38.47, 38.35, 37.94, 37.20, 37.05, 34.35, 32.26, 30.70, 29.76, 29.43, 29.12, 27.97, 25.57, 23.71, 21.00, 19.44, 18.26, 16.58, 16.23, 16.04, 14.78.

ESI-MS: m/z Calcd. for $C_{35}H_{54}O_6$$[M+H]^+$: 571.3993; Found: 571.3994.

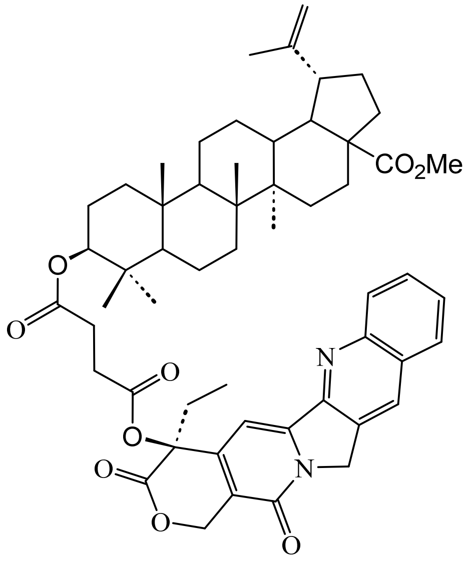

10

(*S*)-4-ethyl-3,14-dioxo-3,4,12,14-tetrahydro-1*H*-pyrano[3',4':6,7]indolizino[1,2-*b*]quinolin-4-yl(methyl (3β-O-carboxypropionyl))lup20(29)-en-28-oate.

Yellow solid (93.6 mg, 74% yield). ^{1}H NMR (300 MHz, Chloroform-*d*) δ 8.40 (s, 1H), 8.27 (d, *J* = 8.5 Hz, 1H), 7.95 (d, *J* = 8.2 Hz, 1H), 7.85 (t, *J* = 7.7 Hz, 1H), 7.67 (t, *J* = 7.5 Hz, 1H), 7.32 (s, 1H), 5.68 (d, *J* = 17.3 Hz, 1H), 5.41 (d, *J* = 17.2 Hz, 1H), 5.29 (s, 2H), 4.76 (s, 1H), 4.63 (s, 1H), 4.38 (dd, *J* = 10.6, 5.2 Hz, 1H), 3.67 (s, 3H), 3.07 – 2.93 (m, 1H), 2.93 – 2.79 (m, 2H), 2.70 – 2.58 (m, 3H), 2.30 (dd, *J* = 14.5, 7.7 Hz, 1H), 2.17 (dd, *J* = 17.2, 10.3 Hz, 3H), 1.90 (d, *J* = 11.6 Hz, 2H), 1.69 – 1.47 (m, 4H), 1.45 – 1.34 (m, 6H), 1.34 – 1.29 (m, 3H), 1.27 (s, 4H), 1.13 (s, 3H), 1.05 – 0.82 (m, 10H), 0.77 (s, 3H), 0.70 (d, *J* = 5.1 Hz, 6H), 0.57 (d, *J* = 8.7 Hz, 1H).

^{13}C NMR (75 MHz, $CDCl_3$) δ 176.68, 171.51, 171.41, 167.45, 157.41, 152.40, 150.63, 148.85, 146.12, 145.90, 131.22, 130.73, 129.74, 128.55, 128.22, 128.09, 120.38, 109.65, 96.58, 81.52, 76.21, 67.12, 56.61, 55.45, 51.30, 50.44, 49.98, 49.56, 47.04, 42.43, 40.70, 38.34, 38.30, 37.81, 37.02, 34.25, 32.23, 31.88, 30.69, 29.72, 29.32, 29.12, 27.95, 25.56, 23.61, 20.90, 19.48, 18.14, 16.56, 16.05, 15.94, 14.85, 7.66.

ESI-MS: m/z Calcd. for $C_{55}H_{68}N_2O_9$ $[M+Na]^+$: 923.4817; Found: 923.4817.

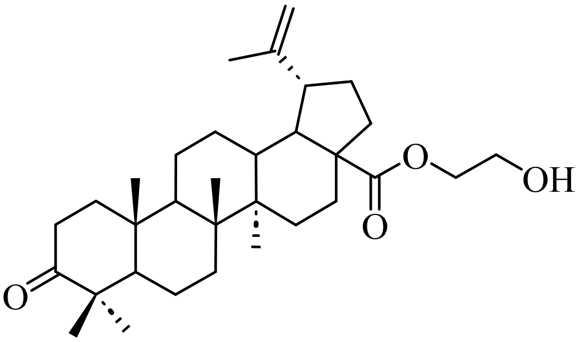

20

2-hydroxyethyl(3*β*)-3-(oxo)lup-20(29)-en-28-oate.

White solid (319.2 mg, 79% yield). ^{1}H NMR (300 MHz, Chloroform-*d*) δ 4.74 (s, 1H), 4.62 (s, 1H), 4.38 – 4.06 (m, 3H), 3.85 (t, *J* = 4.7 Hz, 2H), 3.10 – 2.89 (m, 1H), 2.59 – 2.33 (m, 2H), 2.33 – 2.18 (m, 3H), 2.05 (s, 1H), 1.99 – 1.82 (m, 3H), 1.67 – 1.14 (m, 16H), 1.05 (d, *J* = 14.4 Hz, 6H), 0.98 (d, *J* = 5.8 Hz, 6H), 0.93 (s, 4H), 0.88 (d, *J* = 7.4 Hz, 1H).

^{13}C NMR (75 MHz, $CDCl_3$) δ 218.16, 176.65, 150.39, 109.80, 65.72, 61.69, 56.70, 55.08, 50.00, 49.47, 47.40, 47.00, 42.55, 40.74, 39.71, 38.44, 37.08, 36.99, 34.20, 33.69, 32.17, 30.65, 29.73, 26.68, 25.62, 21.50, 21.10, 19.70, 19.45, 16.01, 15.81, 14.70.

ESI-MS: m/z Calcd. for $C_{32}H_{50}O_4$ [M+H]$^+$: 499.3781; Found: 499.3782.

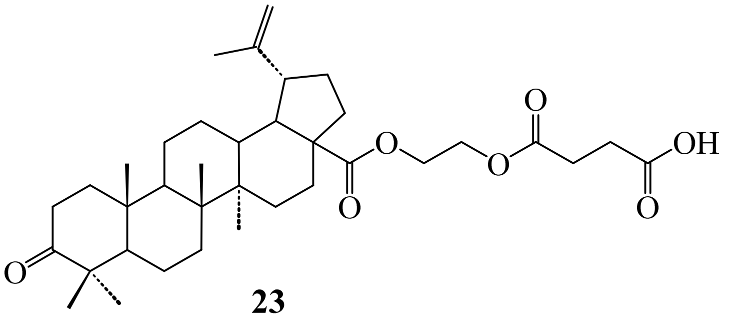

23

(3*β*)-3-(oxo)lup-20(29)-en-28-carbonyloxyethyl)succinic acid.

White solid (194.02 mg, 81% yield). ^{1}H NMR (300 MHz, Chloroform-*d*) δ 10.96 (s, 1H), 5.23 (s, 1H), 4.64 (s, 1H), 4.51 (s, 1H), 4.29 – 4.15 (m, 3H), 2.96 – 2.90 (m, 1H), 2.90 – 2.82 (m, 1H), 2.59 – 2.51 (m, 8H), 2.47 – 2.25 (m, 2H), 2.20 – 2.07 (m, 2H), 1.88 – 1.76 (m, 3H), 1.37 (s, 2H), 1.36 – 1.12 (m, 12H), 1.09 (d, *J* = 9.0 Hz, 1H), 0.97 (s, 3H), 0.88 (dd, *J* = 18.0, 10.8 Hz, 12H).

^{13}C NMR (75 MHz, $CDCl_3$) δ 218.56, 177.29, 175.66, 171.73, 150.10, 109.58, 62.50, 61.34, 56.40, 54.78, 49.71, 49.17, 47.17, 46.78, 42.30, 40.47, 39.43, 38.22, 36.74, 33.91, 33.45, 31.82, 30.38, 29.41, 28.66, 28.58, 26.45, 25.37, 21.27, 20.85, 19.47, 19.19, 15.75, 15.59, 14.46.

ESI-MS: m/z Calcd. for $C_{36}H_{54}O_7$[M+H]$^+$: 599.3942; Found: 599.3943.

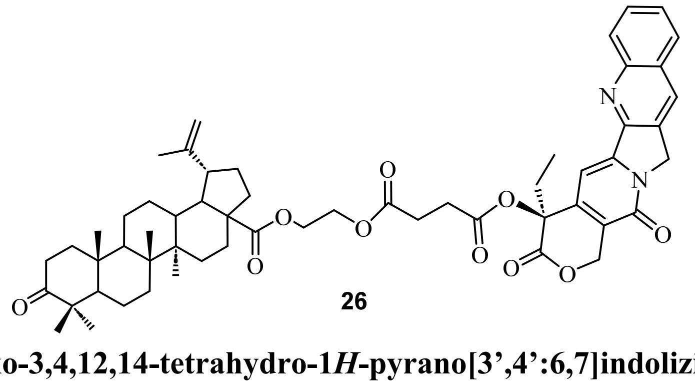

26

(*S*)-4-ethyl-3,14-dioxo-3,4,12,14-tetrahydro-1*H*-pyrano[3',4':6,7]indolizino[1,2-*b*]quinolin-4-yl (3*β*)-3-(oxo)lup-20(29)-en-28-carbonyloxyethyl)hemisuccinate.

Light yellow solid (240.83 mg, 80% yield). ^{1}H NMR (300 MHz, Chloroform-*d*) δ 8.43 (s, 1H), 8.26 (d, *J* = 8.5 Hz, 1H), 7.96 (d, *J* = 8.2 Hz, 1H), 7.86 (t, *J* = 7.7 Hz, 1H), 7.69 (t, *J* = 7.5 Hz, 1H), 7.34 (s, 1H), 5.69 (d, *J* = 17.3 Hz, 1H), 5.40 (d, *J* = 17.3 Hz, 1H), 5.30 (s, 2H), 4.72 (s, 1H),

4.60 (s, 1H), 4.41 – 4.25 (m, 2H), 4.25 (s, 2H), 3.03 – 2.91 (m, 1H), 2.91 – 2.83 (m, 2H), 2.74 – 2.63 (m, 2H), 2.54 – 2.10 (m, 6H), 1.94 – 1.81 (m, 3H), 1.60 (t, J = 11.3 Hz, 1H), 1.50 – 1.34 (m, 11H), 1.34 – 1.24 (m, 6H), 1.09 – 0.87 (m, 20H).

^{13}C NMR (75 MHz, $CDCl_3$) δ 218.10, 175.83, 171.53, 171.23, 167.32, 157.40, 152.29, 150.39, 148.61, 146.02, 131.51, 130.91, 129.40, 128.64, 128.32, 128.26, 128.20, 120.31, 109.79, 96.62, 77.52, 77.10, 76.68, 76.37, 67.07, 62.79, 61.53, 56.62, 55.07, 50.02, 49.96, 49.40, 47.39, 46.99, 42.52, 40.70, 39.68, 38.43, 36.97, 34.20, 33.69, 32.06, 31.85, 30.61, 29.64, 28.97, 28.82, 26.66, 25.60, 21.47, 21.09, 19.69, 19.41, 15.97, 15.81, 14.68, 7.67.

ESI-MS: m/z Calcd. for $C_{56}H_{68}N_2O_{10}$ $[M+Na]^+$: 951.4766; Found: 951.4766.

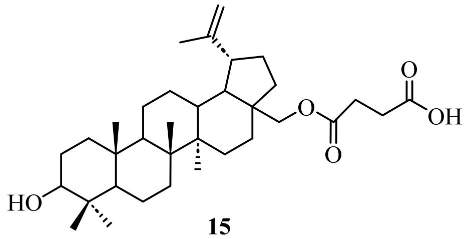

15

28-O-succinyl betulin.

White solid. ^{1}H NMR (300 MHz, DMSO-d_6) δ 12.21 (s, 1H), 4.71 (s, 1H), 4.57 (s, 1H), 4.27 (d, J = 10.8 Hz, 2H), 3.78 (d, J = 11.0 Hz, 1H), 3.04 – 2.92 (m, 1H), 2.10 – 1.01 (m, 26H), 0.97 (d, J = 12.7 Hz, 8H), 0.89 (s, 4H), 0.79 (d, J = 7.8 Hz, 5H), 0.67 (s, 4H).

^{13}C NMR (75 MHz, DMSO-d_6) δ 173.41, 172.28, 149.81, 109.95, 76.79, 61.64, 54.86, 49.78, 48.19, 47.06, 46.12, 42.22, 40.41, 38.50, 38.27, 37.04, 36.67, 34.02, 33.76, 29.18, 29.02, 28.89, 28.75, 28.09, 27.16, 26.63, 24.74, 20.30, 18.77, 17.97, 15.89, 15.80, 15.62, 14.51.

ESI-MS: m/z Calcd. for $C_{34}H_{54}O_5$$[M-H]^-$: 541.3898; Found: 541.3902.

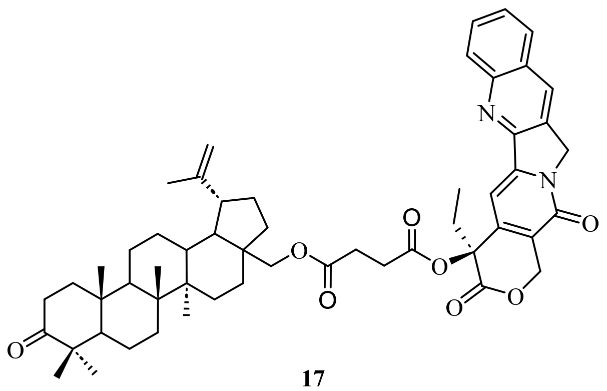

17

(*S*)-4-ethyl-3,14-dioxo-3,4,12,14-tetrahydro-1*H*-pyrano[3’,4’:6,7]indolizino[1,2-*b*]quinolin-4-yl(3*β*)-3-(oxo)lup-20(29)-en-28-O-hemisuccinate.

Light yellow solid (134.6 mg, 70% yield). ^{1}H NMR (300 MHz, Chloroform-d) δ 8.43 (s, 1H), 8.30 (d, J = 8.6 Hz, 1H), 7.96 (d, J = 8.1 Hz, 1H), 7.86 (t, J = 7.7 Hz, 1H), 7.69 (t, J = 7.5 Hz, 1H), 7.37 (s, 1H), 5.69 (d, J = 17.2 Hz, 1H), 5.41 (d, J = 17.2 Hz, 1H), 5.30 (s, 2H), 4.66 (d, J = 2.3 Hz, 1H), 4.58 (s, 1H), 4.22 – 4.07 (m, 2H), 3.99 – 3.89 (m, 1H), 3.50 (s, 1H), 2.95 – 2.83

(m, 2H), 2.75 – 2.64 (m, 2H), 2.56 – 2.26 (m, 3H), 2.26 – 2.09 (m, 1H), 2.06 (s, 1H), 2.02 – 1.87 (m, 1H), 1.87 – 1.68 (m, 2H), 1.65 (s, 3H), 1.62 – 1.48 (m, 4H), 1.44 – 1.14 (m, 12H), 1.13 - 0.93 (m, 10H), 0.89 (d, J = 10.3 Hz, 6H), 0.81 (s, 3H).

^{13}C NMR (75 MHz, $CDCl_3$) δ 218.02, 172.18, 171.38, 167.38, 157.35, 152.29, 150.05, 148.62, 145.95, 131.41, 130.80, 129.47, 128.58, 128.25, 128.20, 128.16, 120.28, 109.88, 96.69, 76.24, 67.03, 63.12, 54.91, 49.96, 49.63, 48.70, 47.67, 47.34, 46.36, 42.66, 40.70, 39.58, 37.56, 36.81, 34.49, 34.14, 33.36, 31.80, 29.63, 29.54, 29.01, 28.98, 26.93, 26.61, 25.11, 21.20, 21.07, 19.57, 19.09, 15.90, 15.53, 14.61, 7.64.MS (ESI): m/z: Calcd for $C_{54}H_{66}N_2O_8[M+H]^+$: 871.4891; Found: 871.4894.

4. ^{1}H NMR, ^{13}C NMR and HRMS/MS spectra of all products.

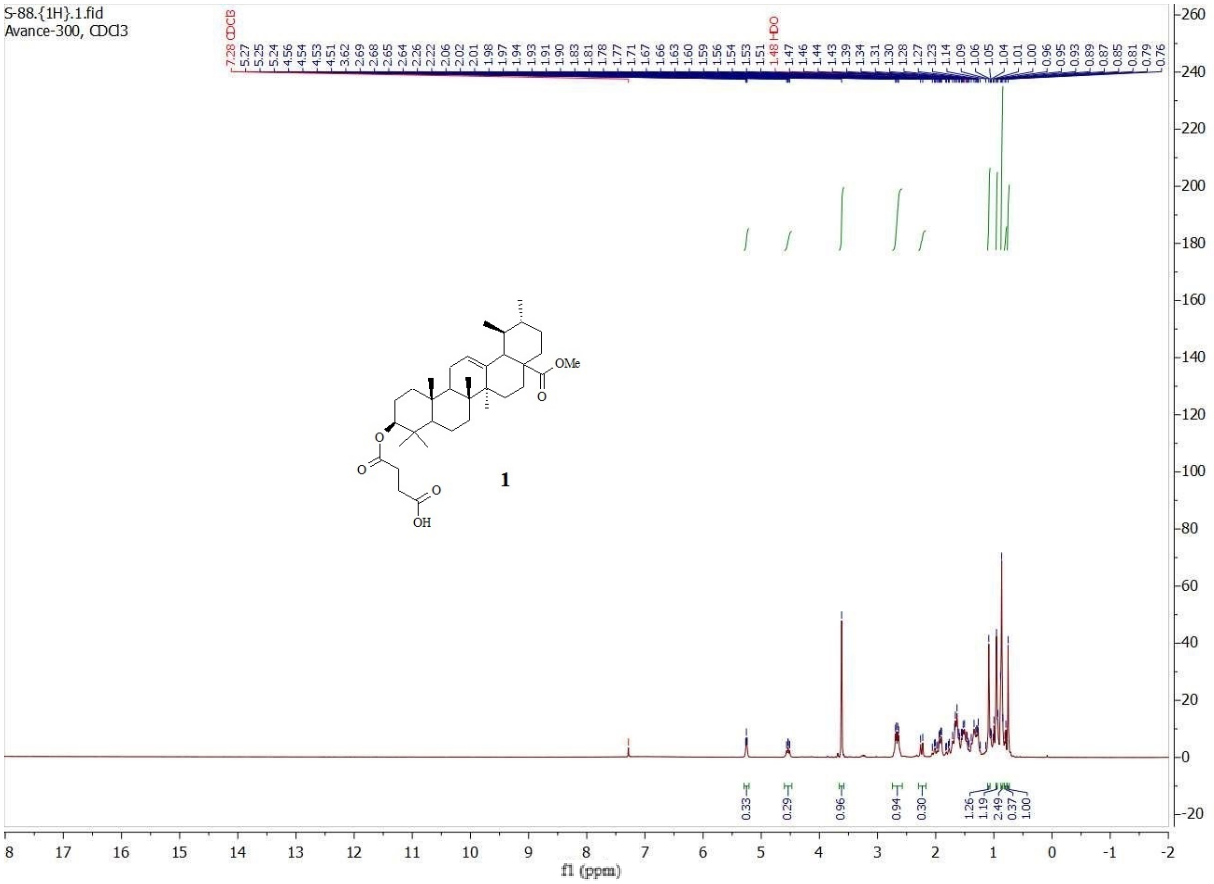

Figure S1 ^{1}H NMR spectra of compound **1.**

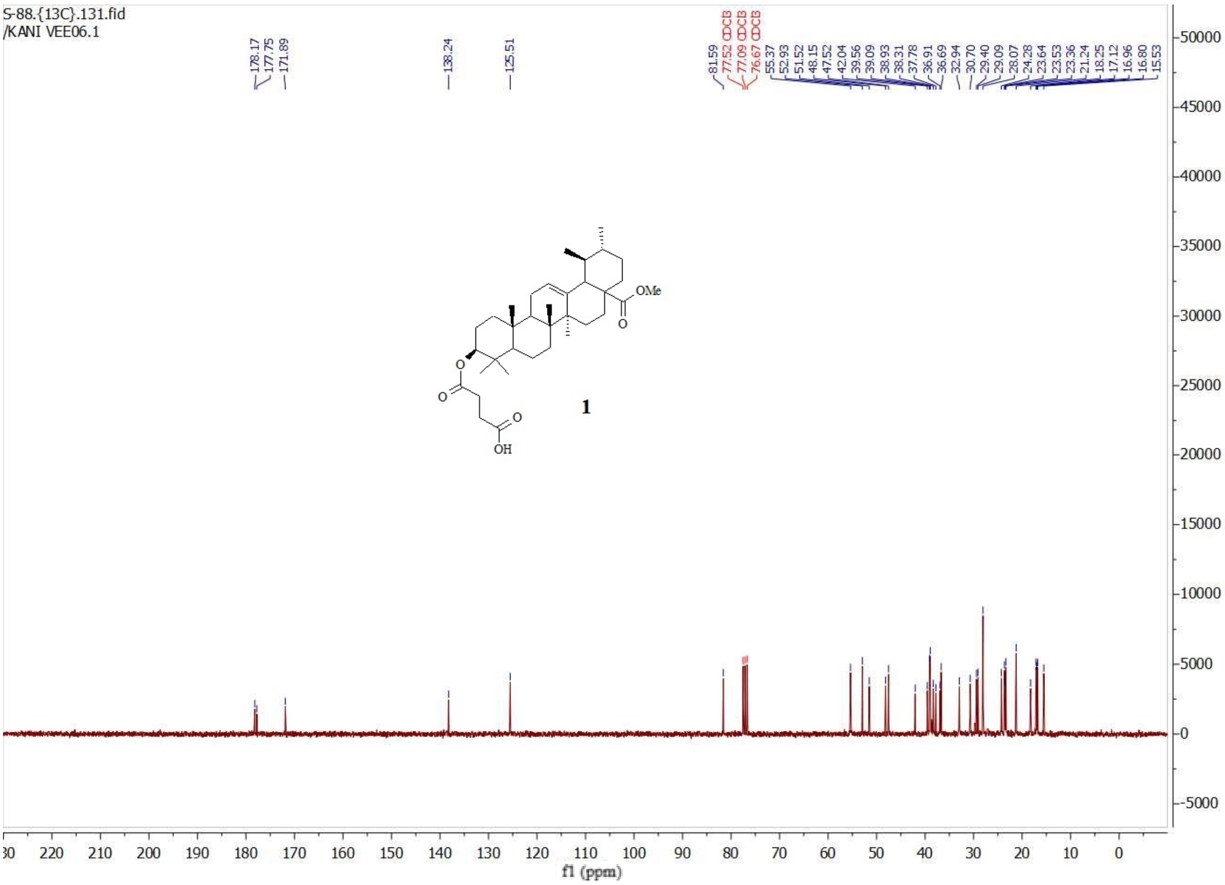

Figure S2 ^{13}C NMR spectra of compound **1.**

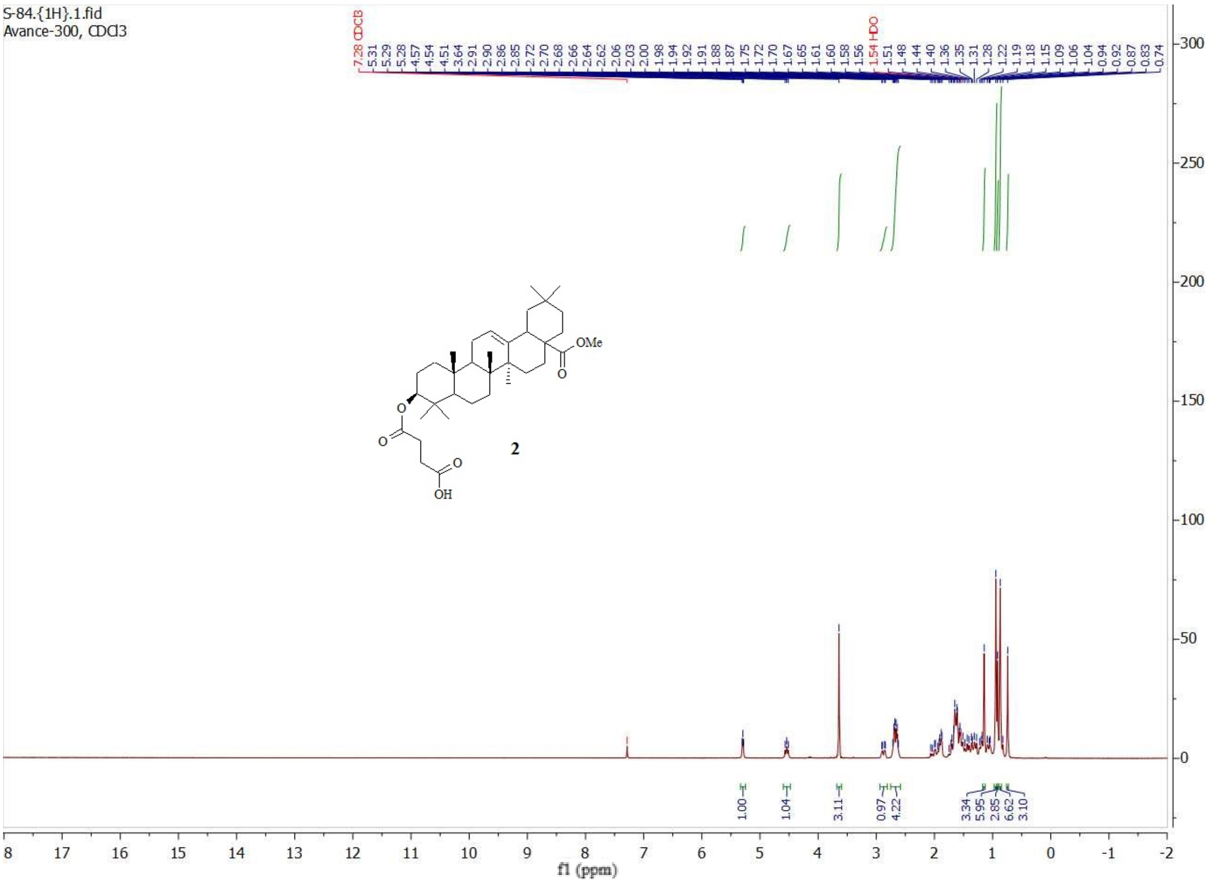

Figure S3 ^{1}H NMR spectra of compound **2.**

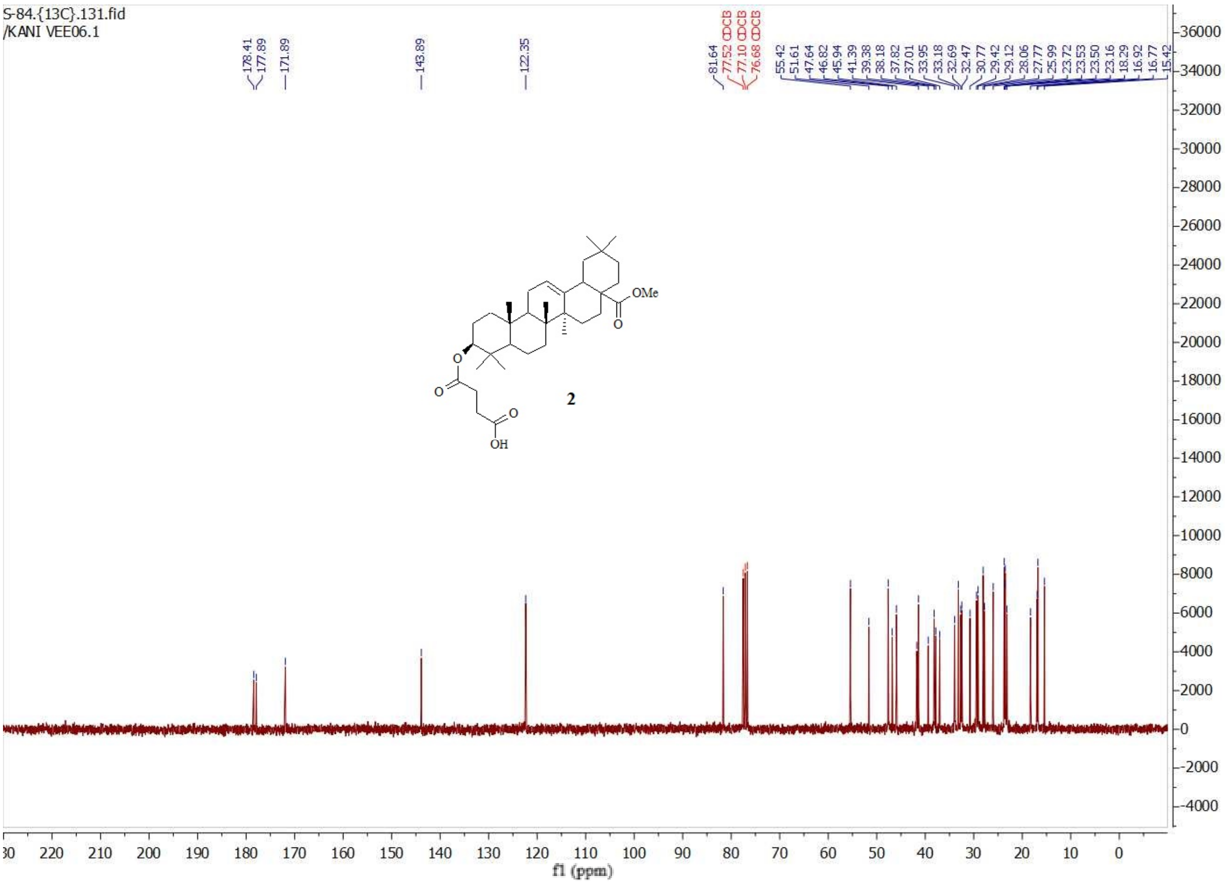

Figure S4 ^{13}C NMR spectra of compound **2.**

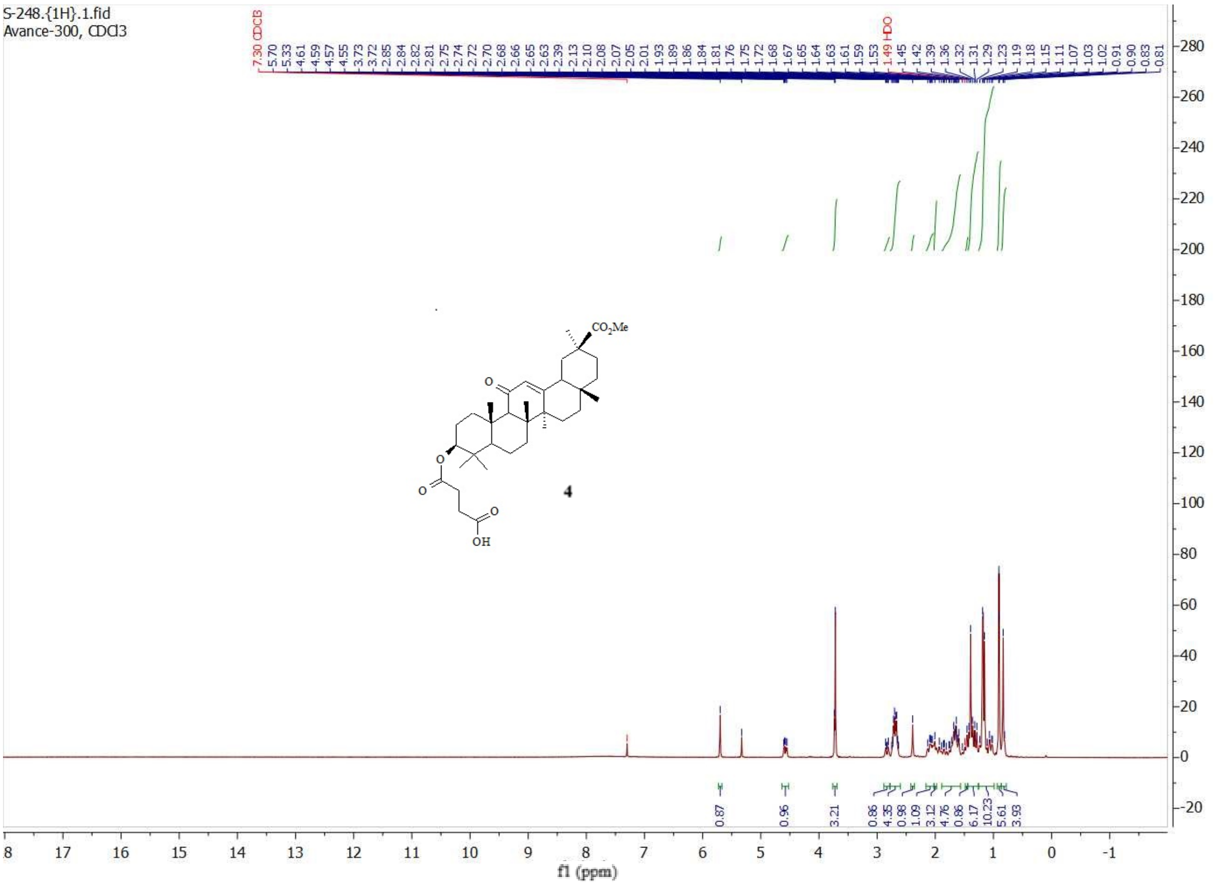

Figure S5 ^{1}H NMR spectra of compound **4.**

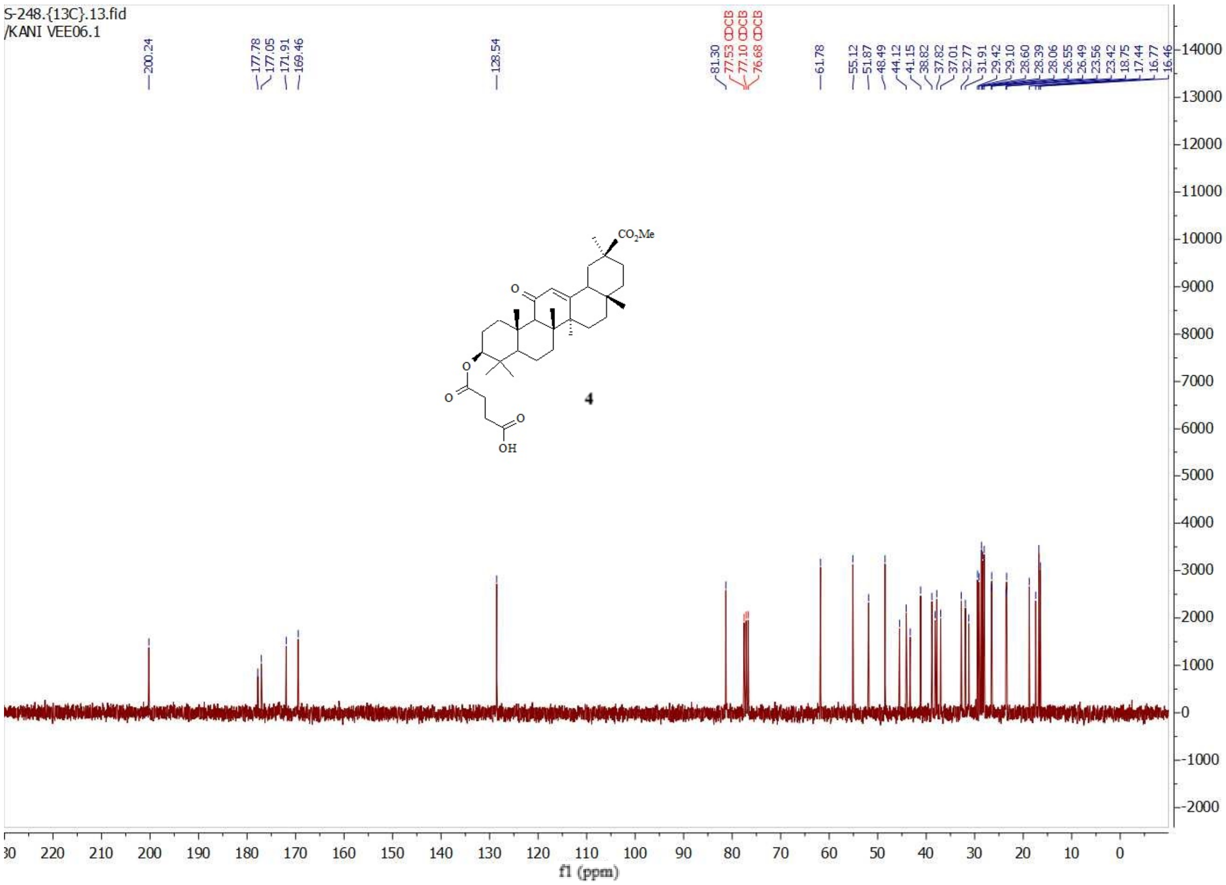

Figure S6 ^{13}C NMR spectra of compound **4.**

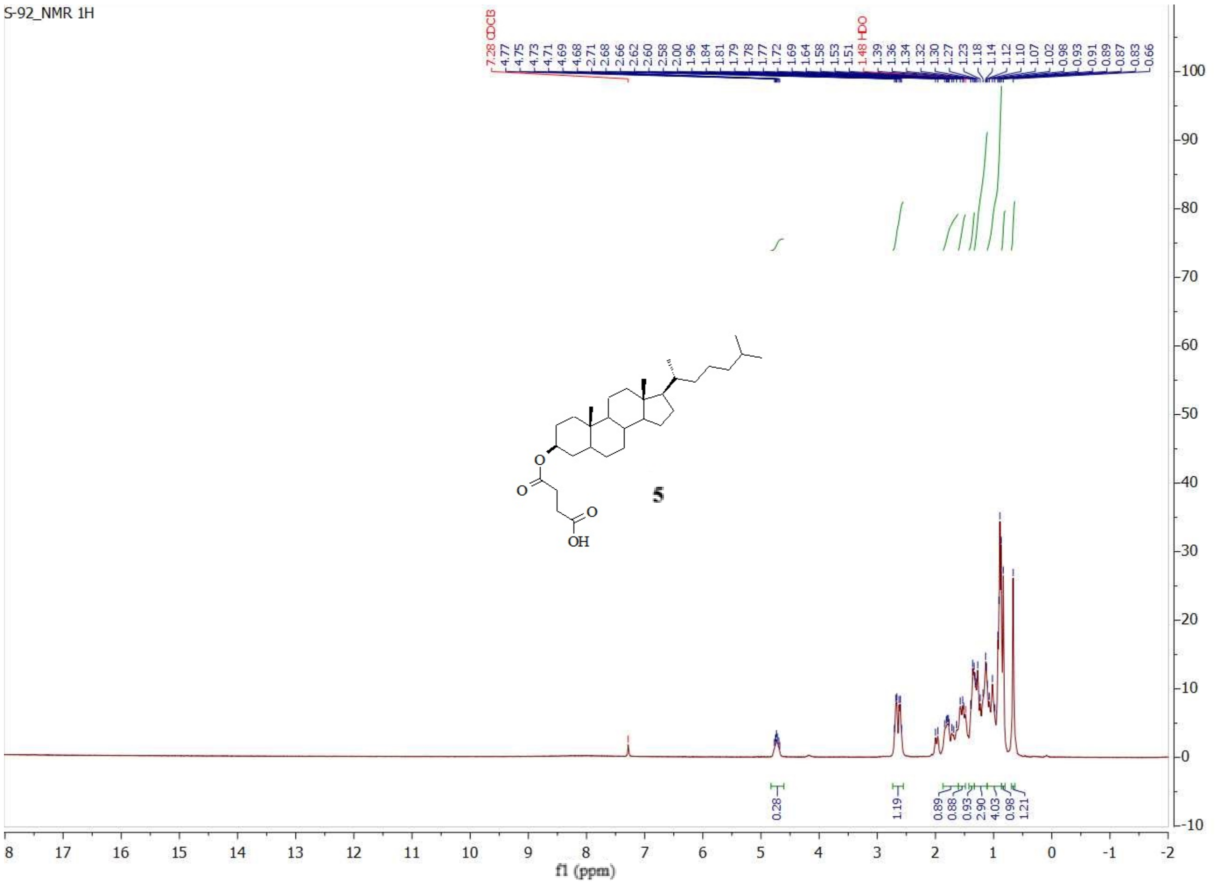

Figure S7 ^{1}H NMR spectra of compound **5.**

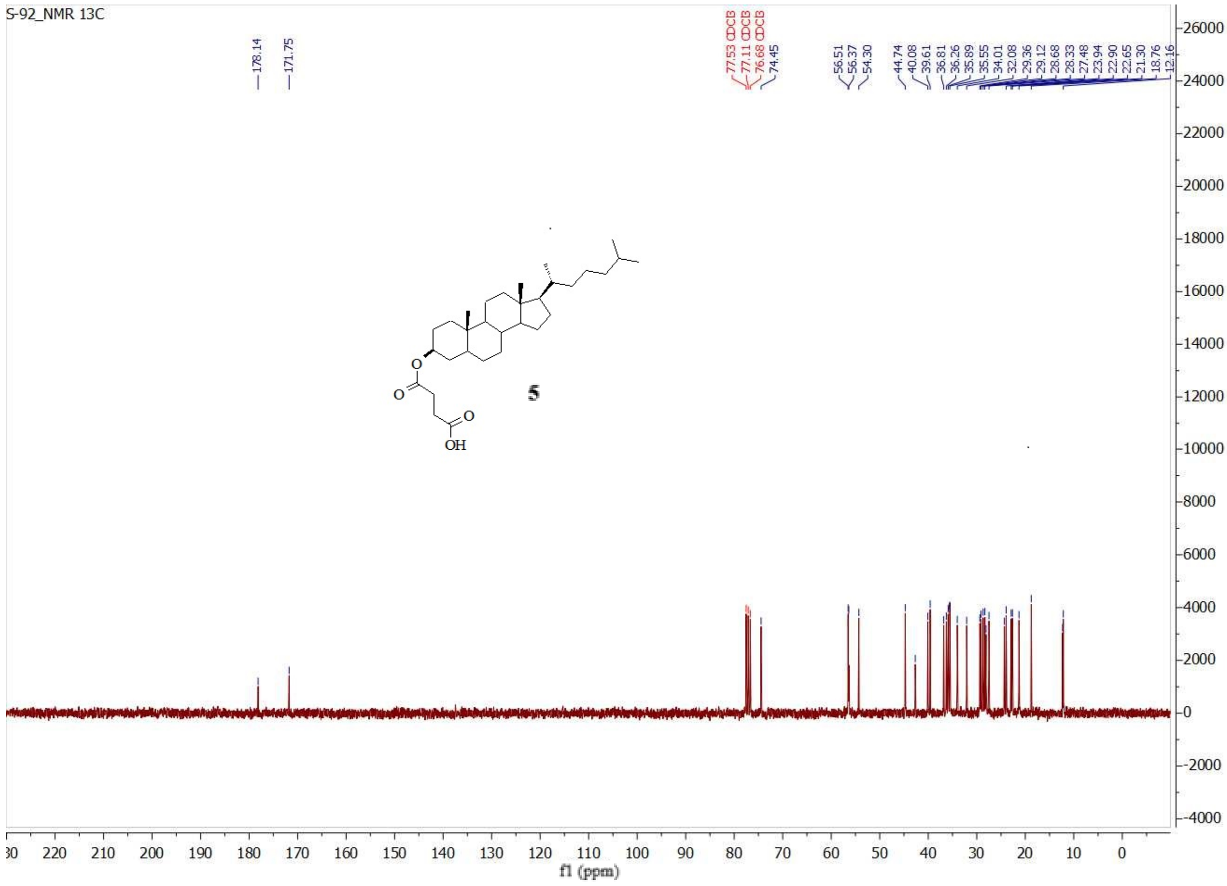

Figure S8 ^{13}C NMR spectra of compound **5.**

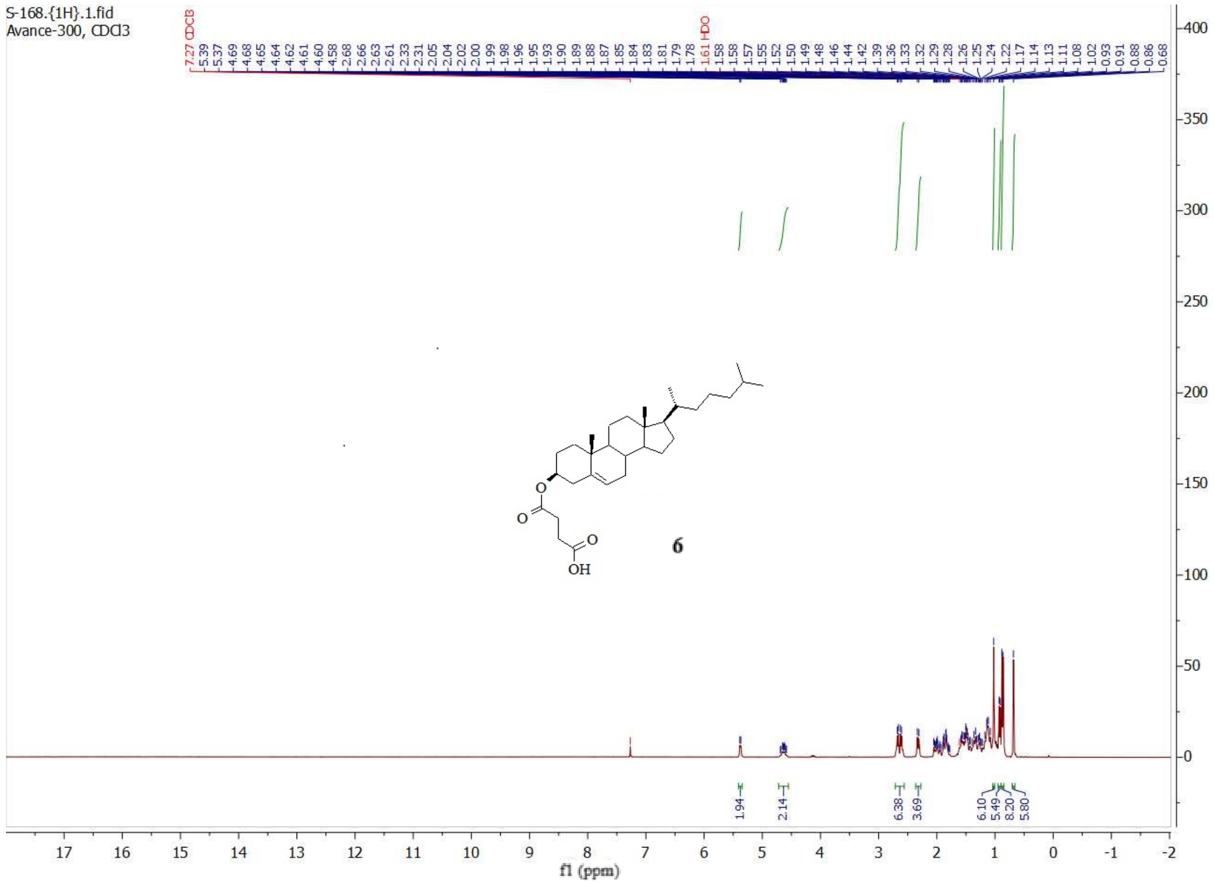

Figure S9 ^{1}H NMR spectra of compound **6.**

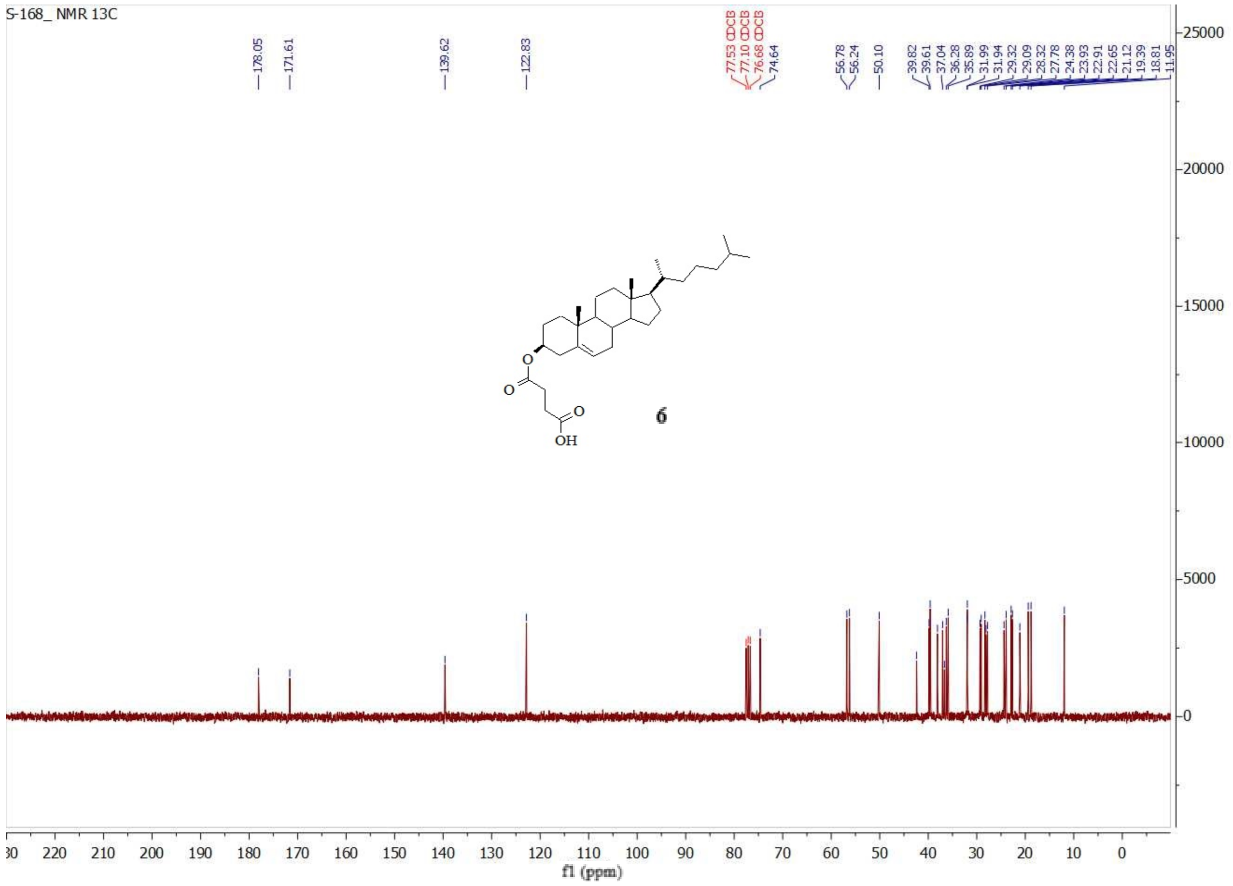

Figure S10 ^{13}C NMR spectra of compound **6.**

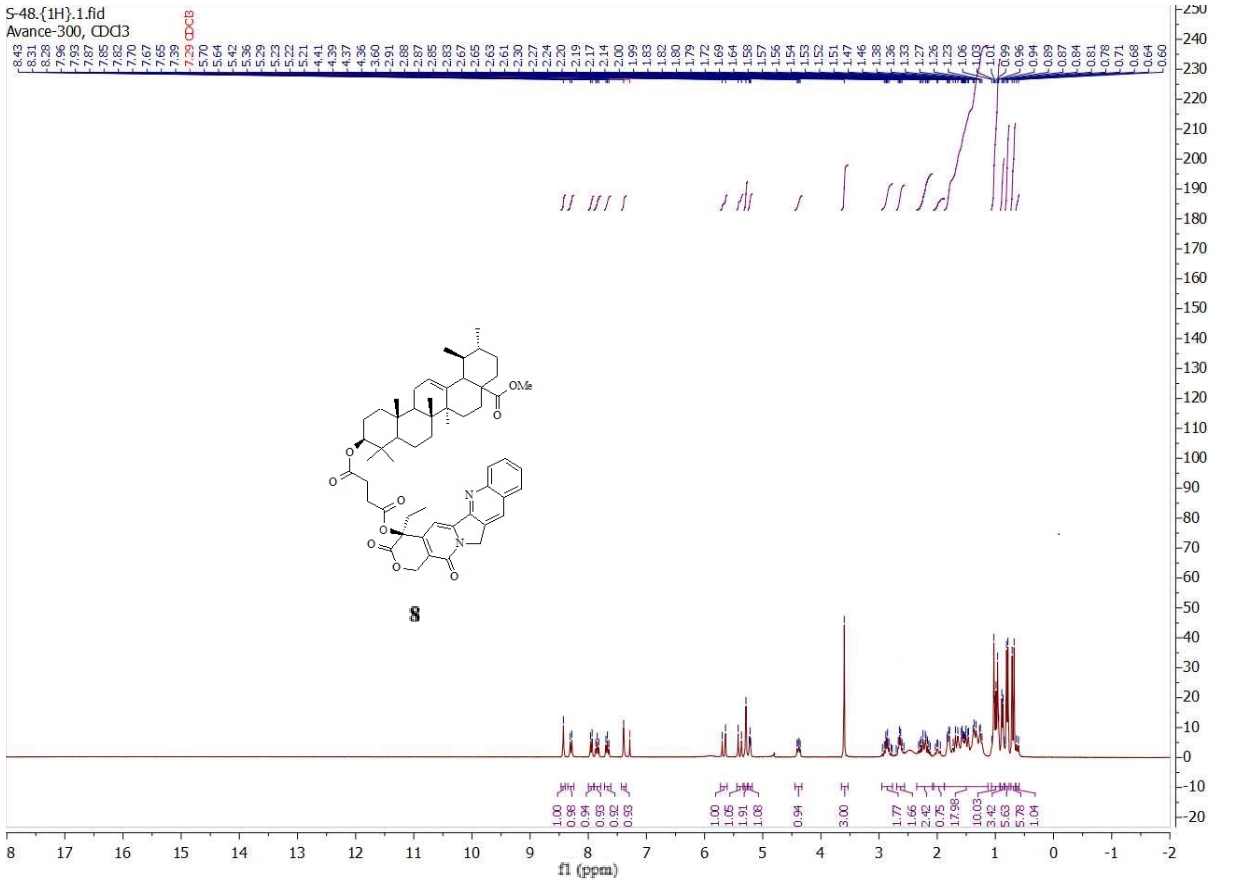

Figure S11 ^{1}H NMR spectra of compound **8.**

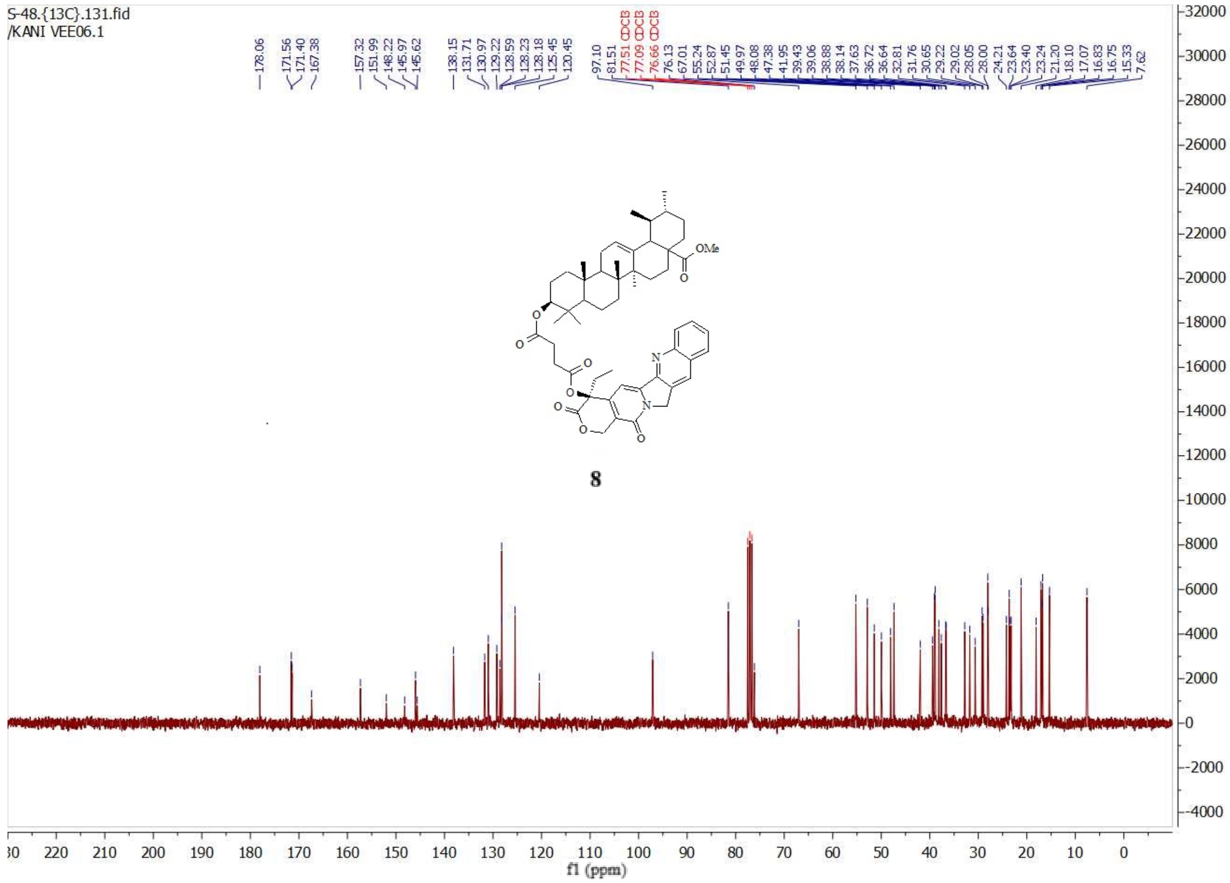

Figure S12 ^{13}C NMR spectra of compound **8.**

Display Report

Analysis Info

Analysis Name	D:\Data\Kolotyrkina\2025\Chobanov\0603017.d	Acquisition Date	03.06.2025 12:31:36
Method	tune_low.m	Operator	BDAL@DE
Sample Name	/ABCD S-48	Instrument / Ser#	micrOTOF 10248
Comment	C55H68N2O9 dlb added CH3OH		

Acquisition Parameter

Source Type	ESI	Ion Polarity	Positive	Set Nebulizer	0.4 Bar
Focus	Not active			Set Dry Heater	180 °C
Scan Begin	50 m/z	Set Capillary	4500 V	Set Dry Gas	4.0 l/min
Scan End	3000 m/z	Set End Plate Offset	-500 V	Set Divert Valve	Waste

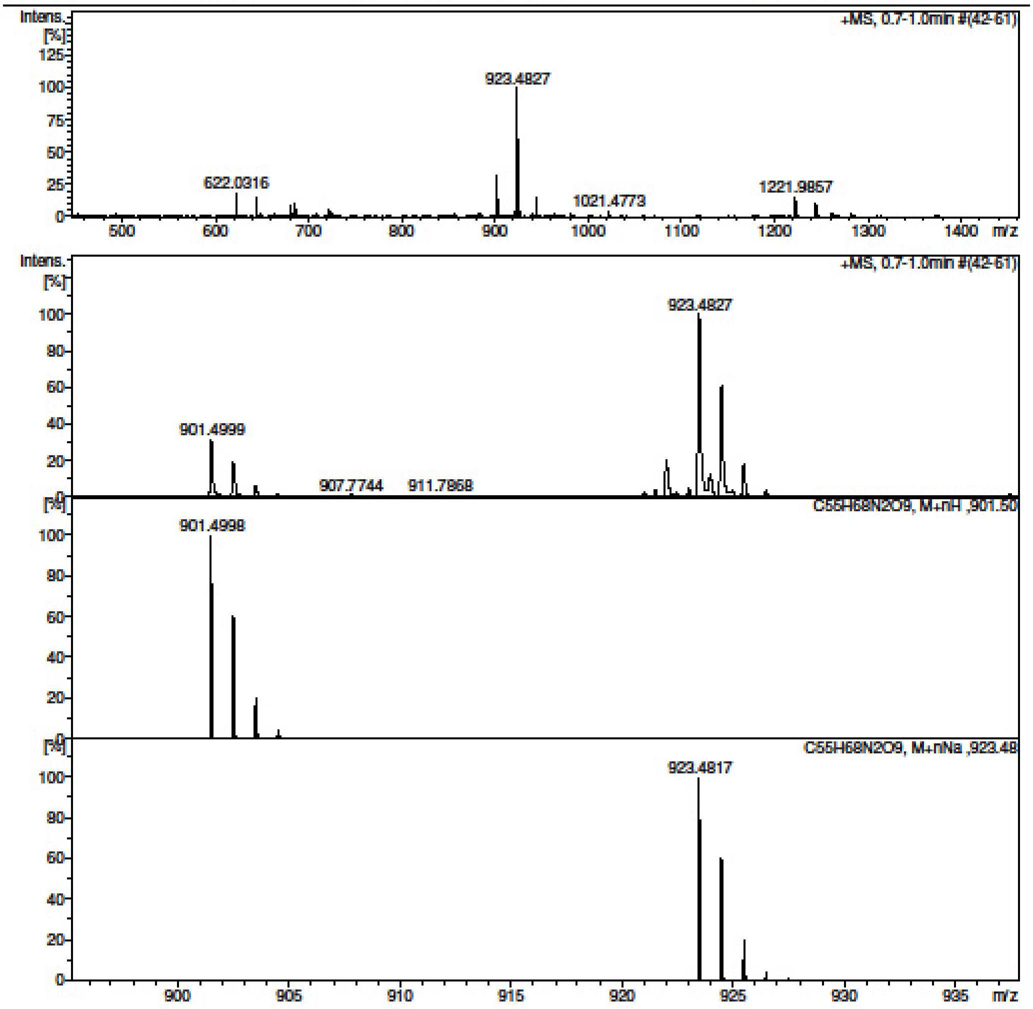

Figure S13 HRMS/MS spectra of compound **8.**

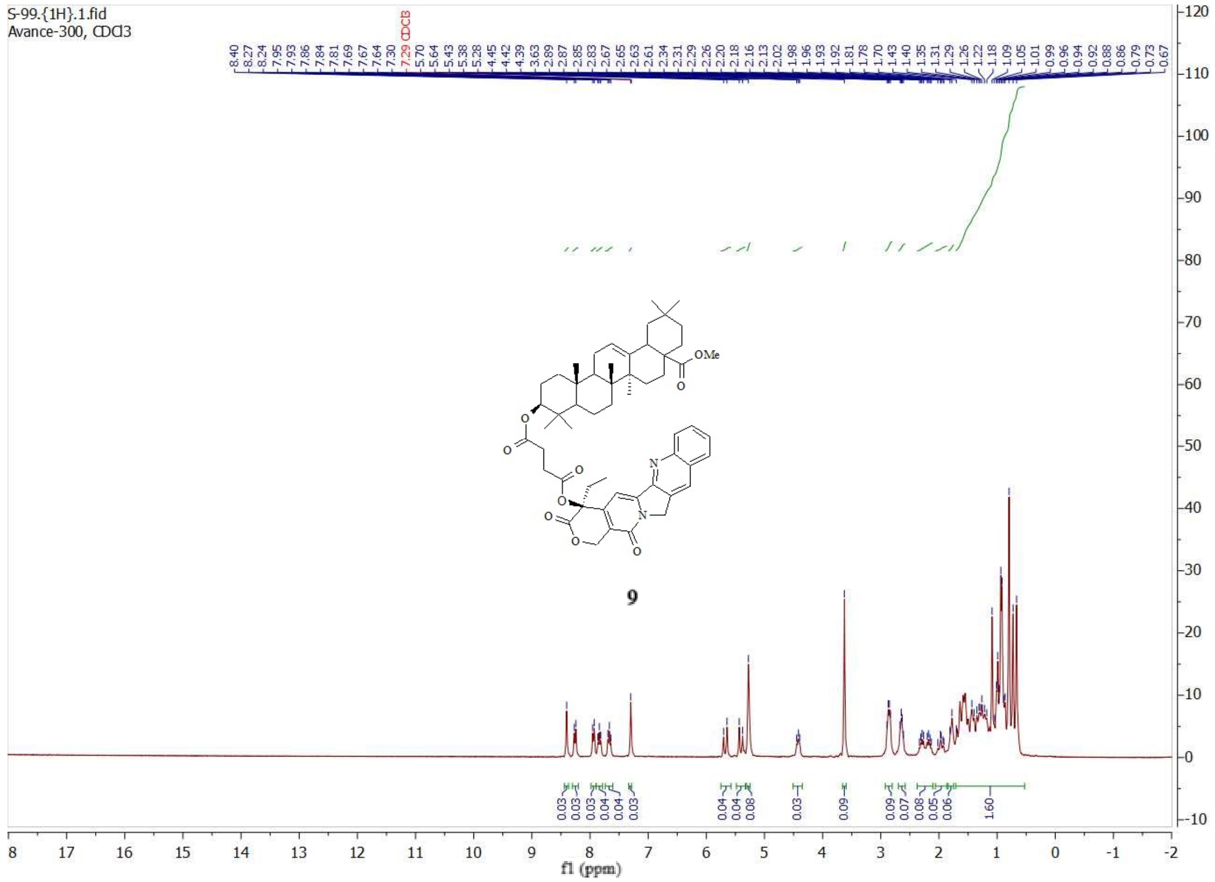

Figure S14 ^{1}H NMR spectra of compound **9.**

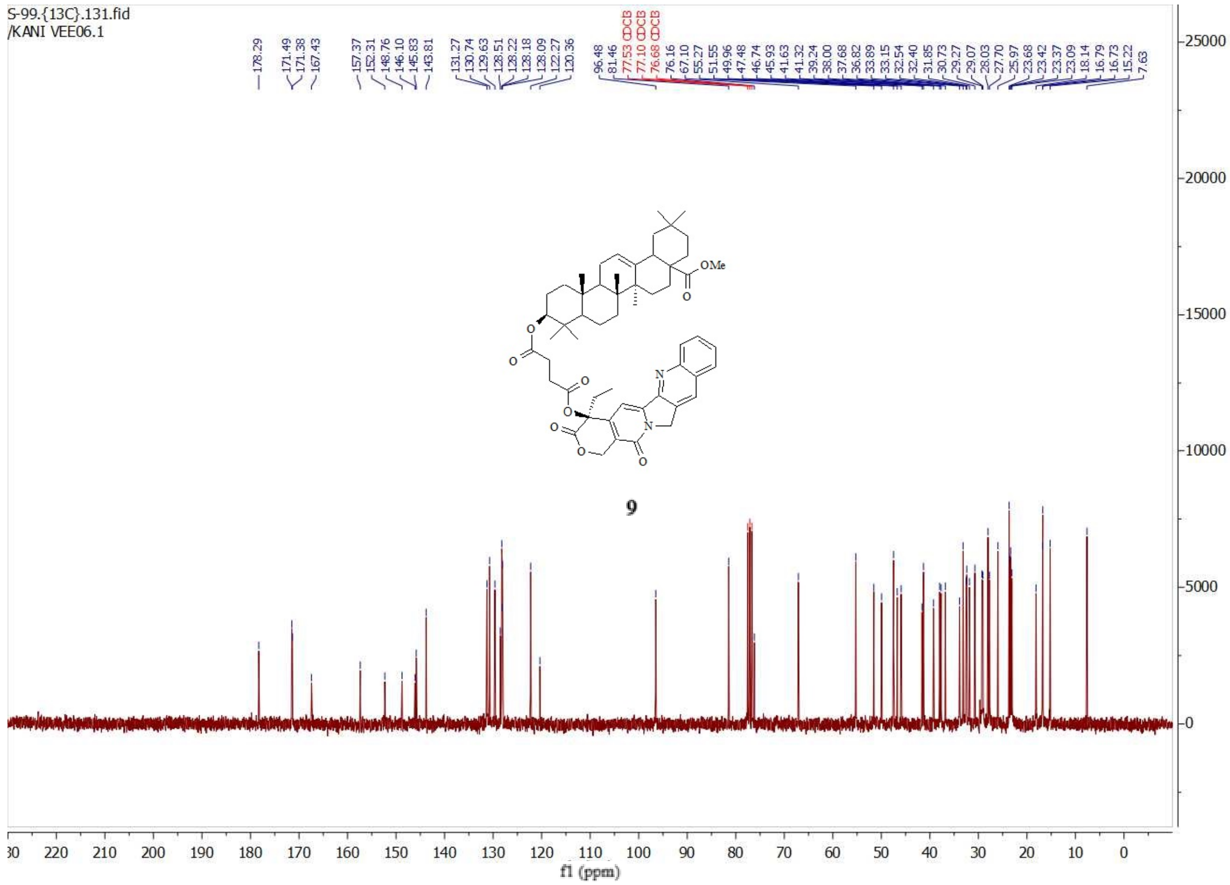

Figure S15 ^{13}C NMR spectra of compound **9.**

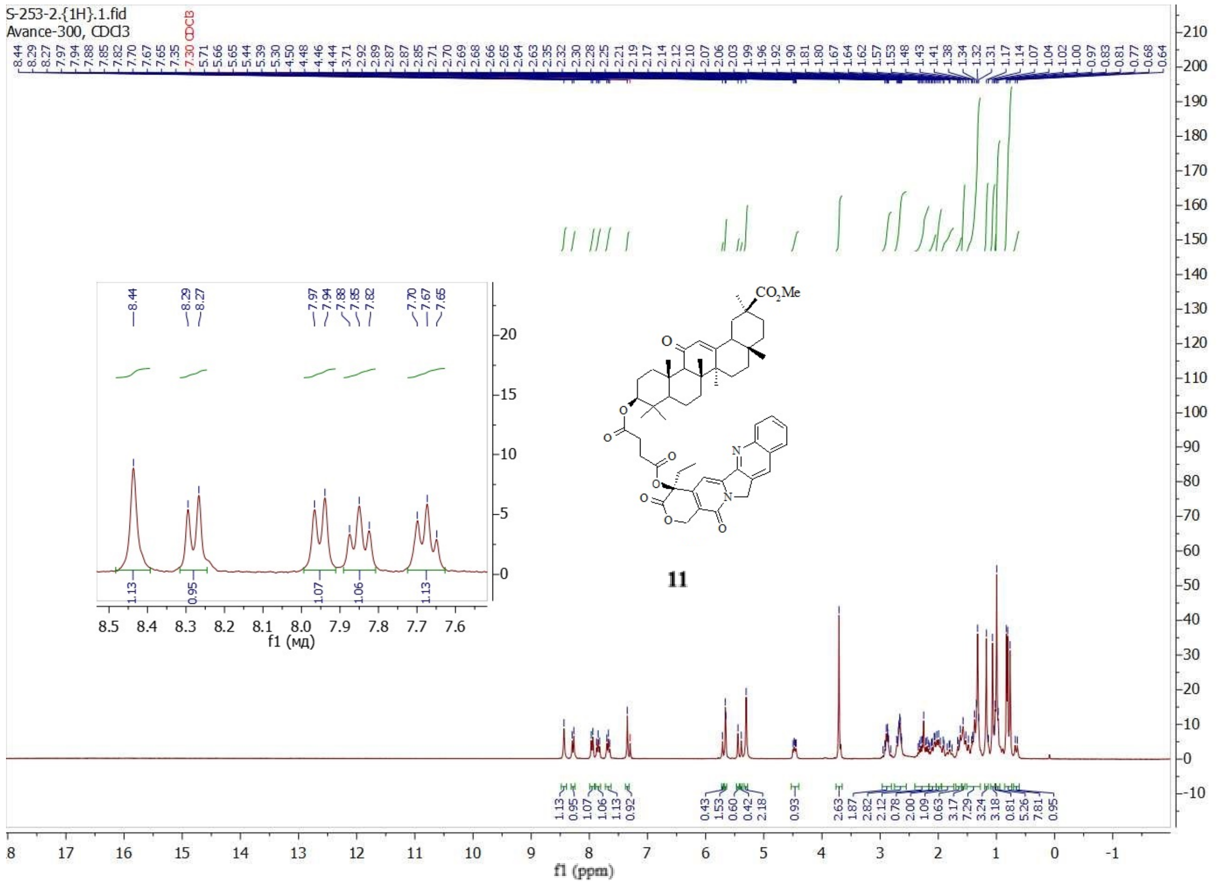

Figure S16 ^{1}H NMR spectra of compound **11.**

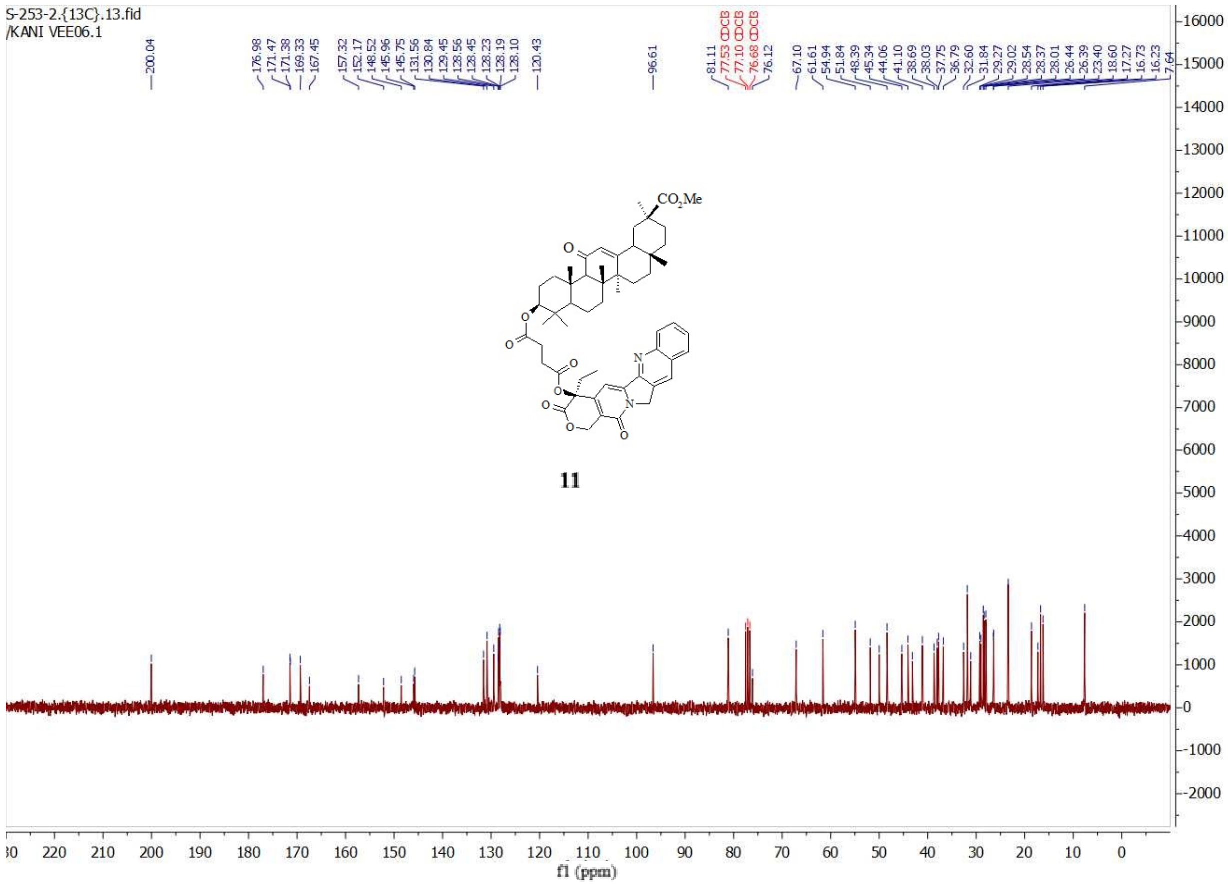

Figure S17 ^{13}C NMR spectra of compound **11.**

Display Report

Analysis Info

Analysis Name	D:\Data\Kolotyrkina\2025\Chobanov\0604014.d	Acquisition Date	04.06.2025 12:39:24
Method	tune_low.m	Operator	BDAL@DE
Sample Name	/ABCD S-253-2	Instrument / Ser#	micrOTOF 10248
Comment	C55H66N1O10 clb added CH3OH		

Acquisition Parameter

Source Type	ESI	Ion Polarity	Positive	Set Nebulizer	0.4 Bar
Focus	Not active			Set Dry Heater	180 °C
Scan Begin	50 m/z	Set Capillary	4500 V	Set Dry Gas	4.0 l/min
Scan End	3000 m/z	Set End Plate Offset	-500 V	Set Divert Valve	Waste

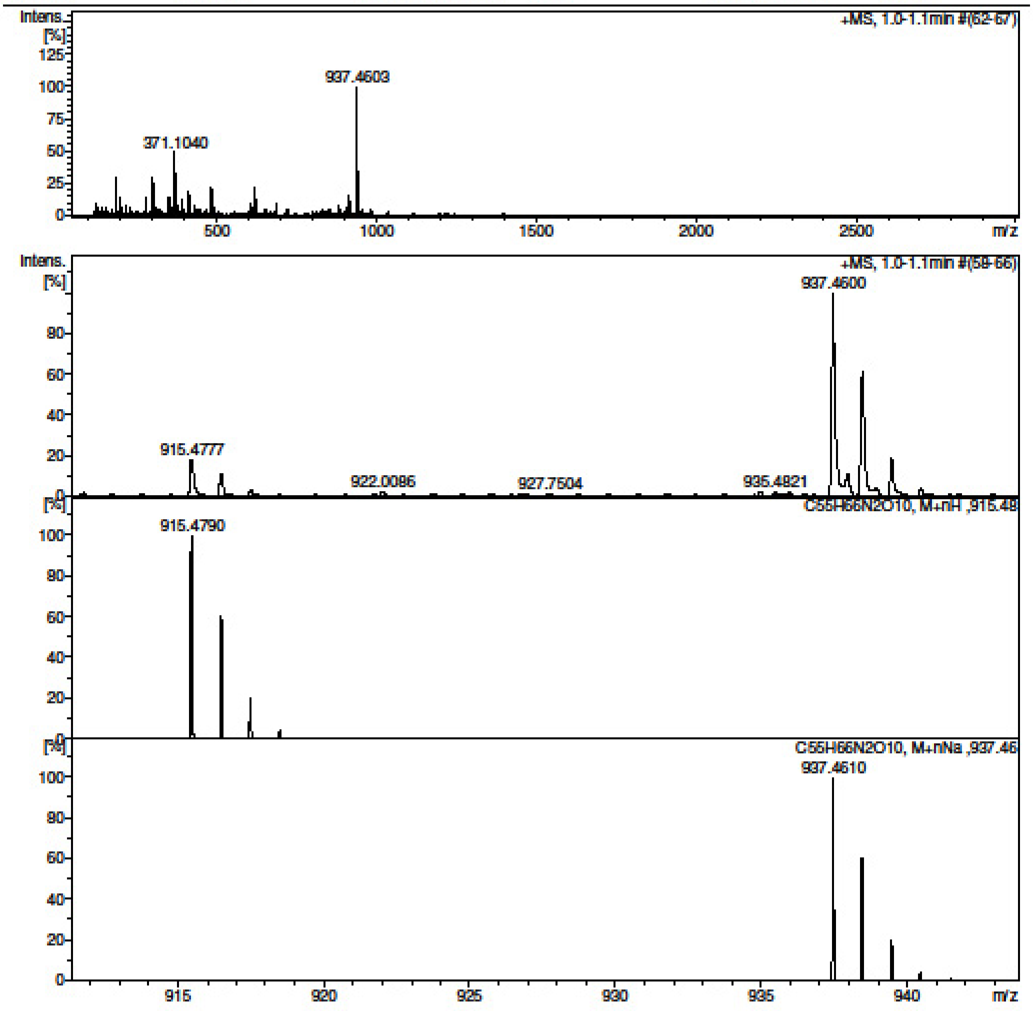

Bruker Compass DataAnalysis 4.0 printed: 04.06.2025 12:44:39 Page 1 of 1

Figure S18 HRMS/MS spectra of compound **11.**

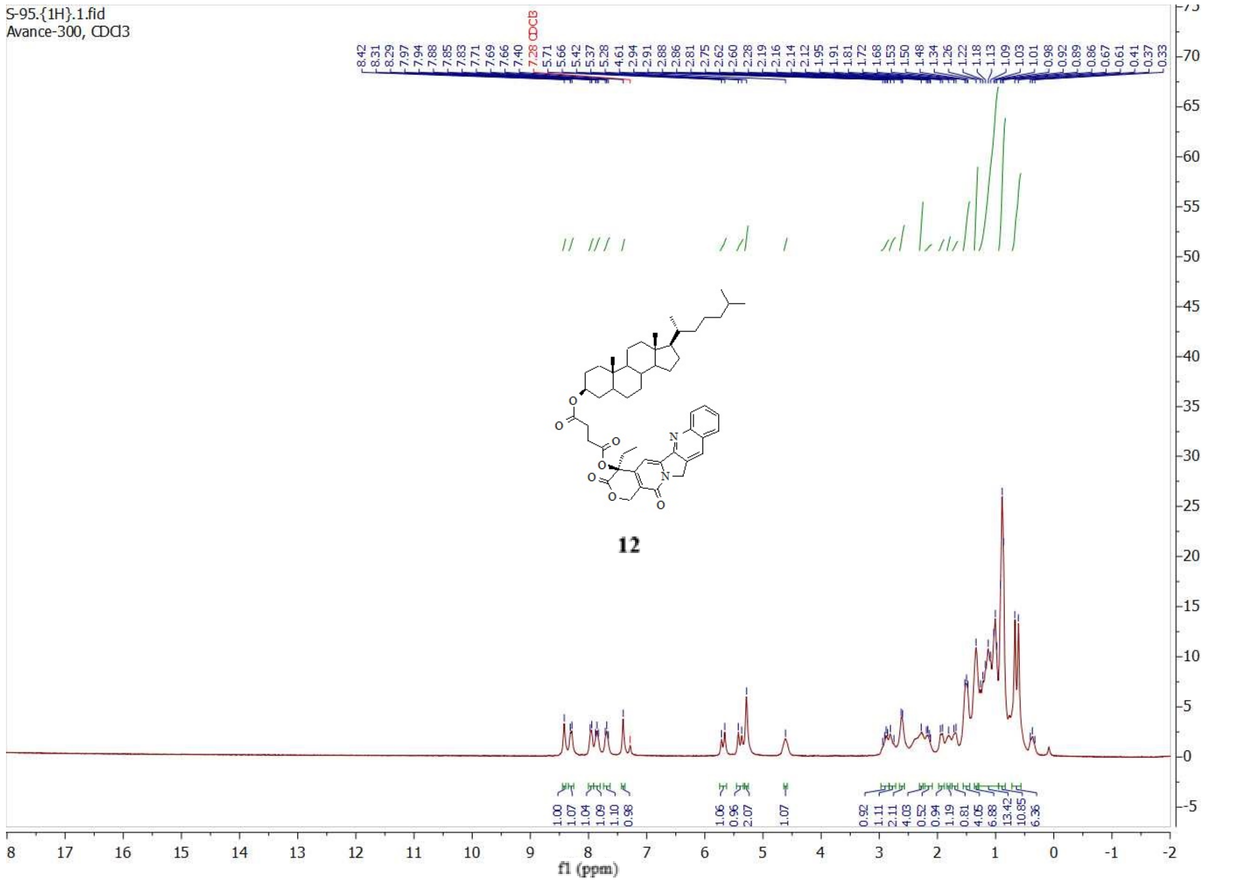

Figure S19 ^{1}H NMR spectra of compound **12.**

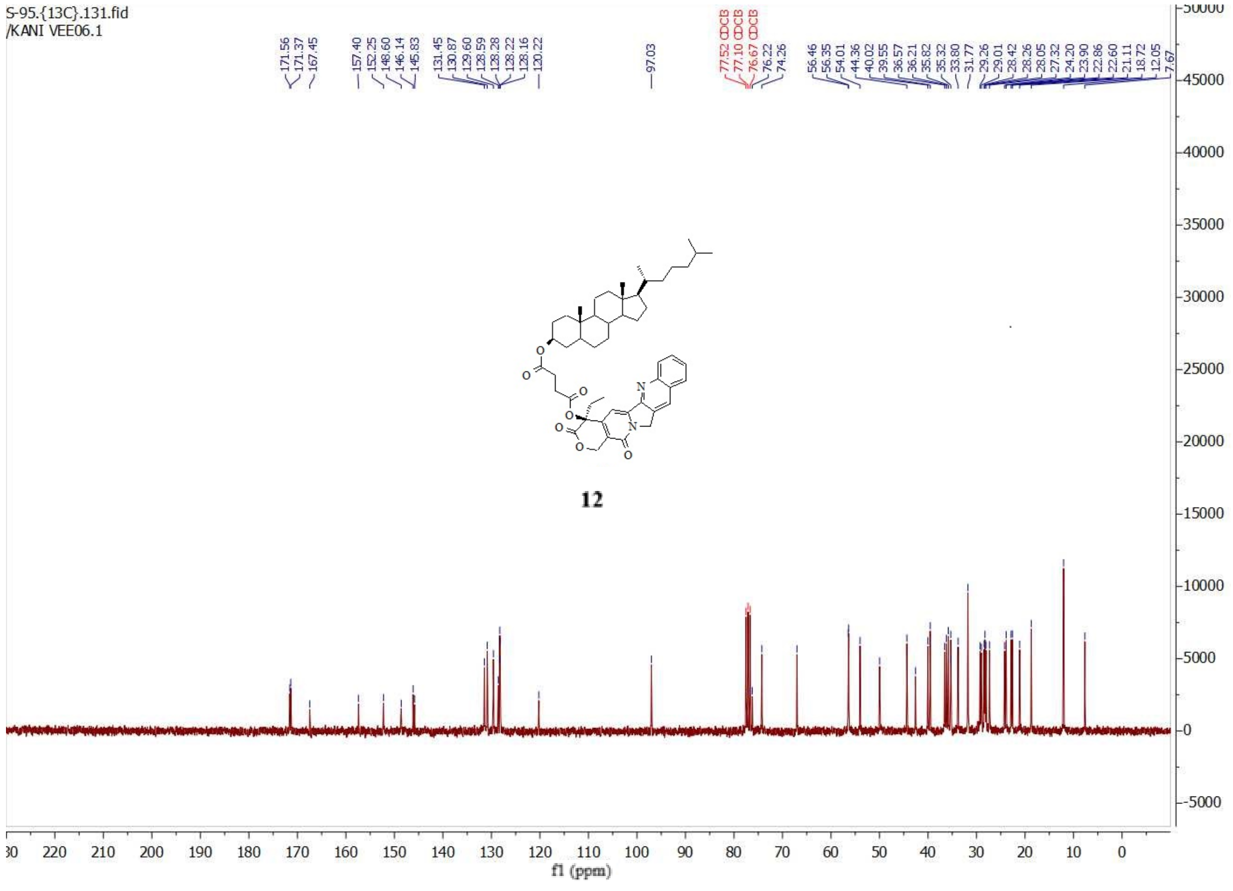

Figure S20 ^{13}C NMR spectra of compound **12.**

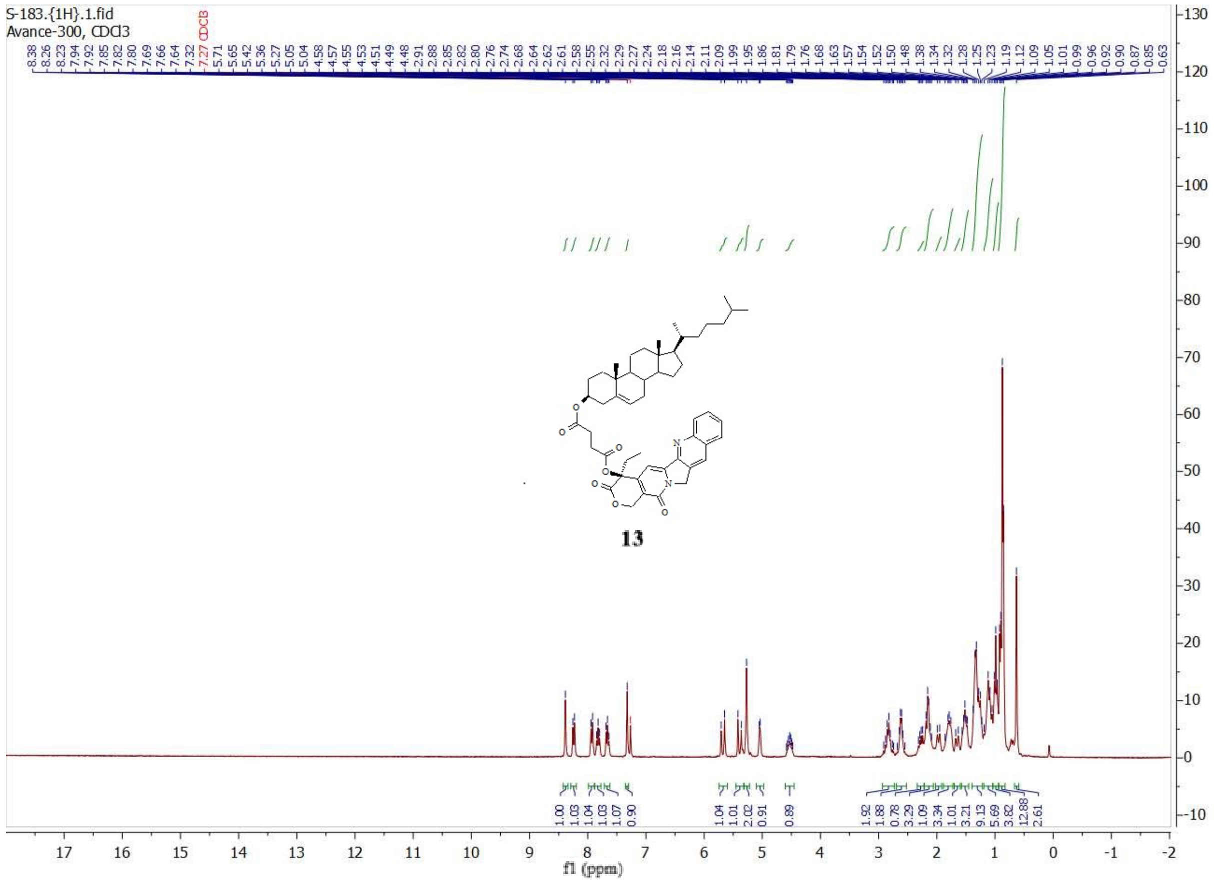

Figure S21 ^{1}H NMR spectra of compound **13.**

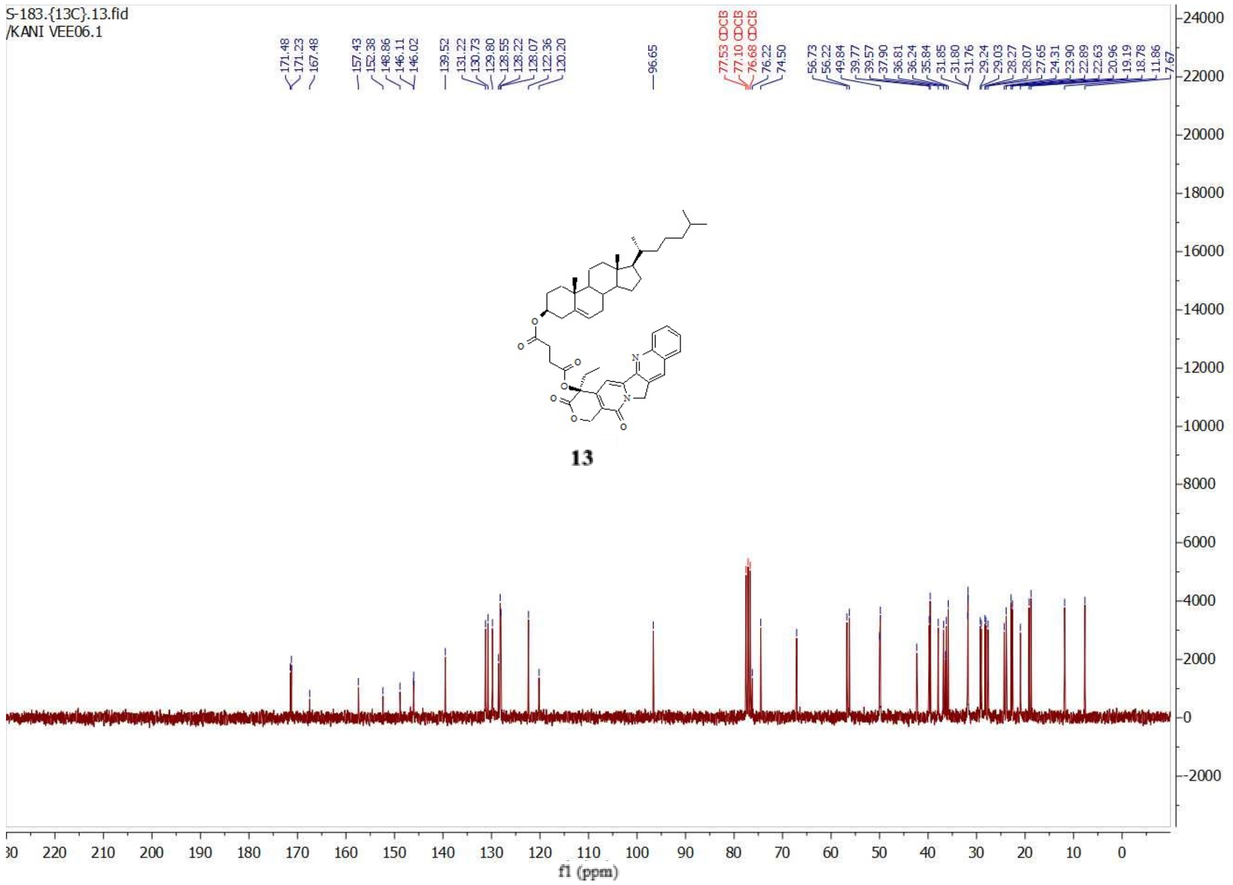

Figure S22 ^{13}C NMR spectra of compound **13.**

Display Report

Analysis Info

Analysis Name	D:\Data\Kolotyrkina\2025\Chobanov\0603019.d	Acquisition Date	03.06.2025 13:03:30
Method	tune_low.m	Operator	BDAL@DE
Sample Name	/ABCD S-183	Instrument / Ser#	micrOTOF 10248
Comment	C51H64N2O7 dlb added CH3OH		

Acquisition Parameter

Source Type	ESI	Ion Polarity	Positive	Set Nebulizer	0.4 Bar
Focus	Not active			Set Dry Heater	180 °C
Scan Begin	50 m/z	Set Capillary	4500 V	Set Dry Gas	4.0 l/min
Scan End	3000 m/z	Set End Plate Offset	-500 V	Set Divert Valve	Waste

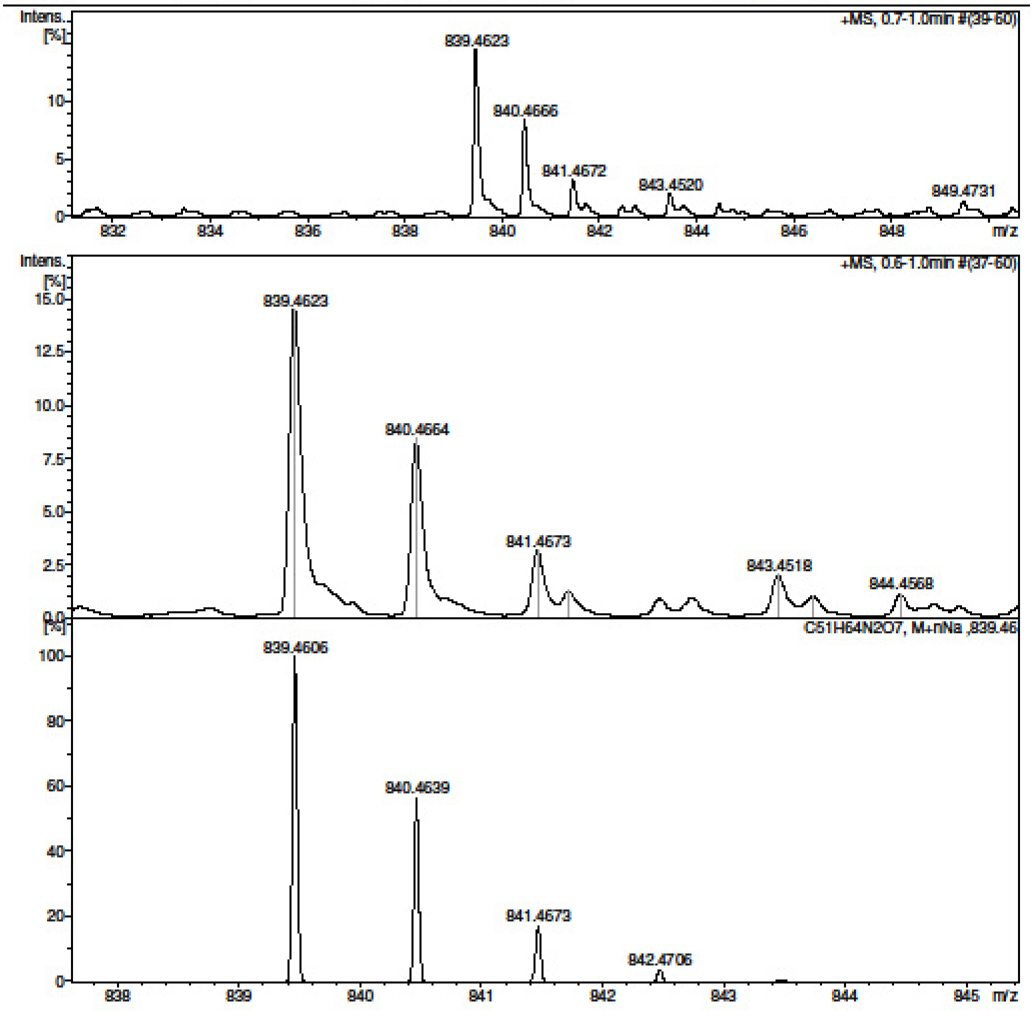

Bruker Compass DataAnalysis 4.0 printed: 03.06.2025 13:15:10 Page 1 of 1

Figure S23 HRMS/MS spectra of compound **13.**

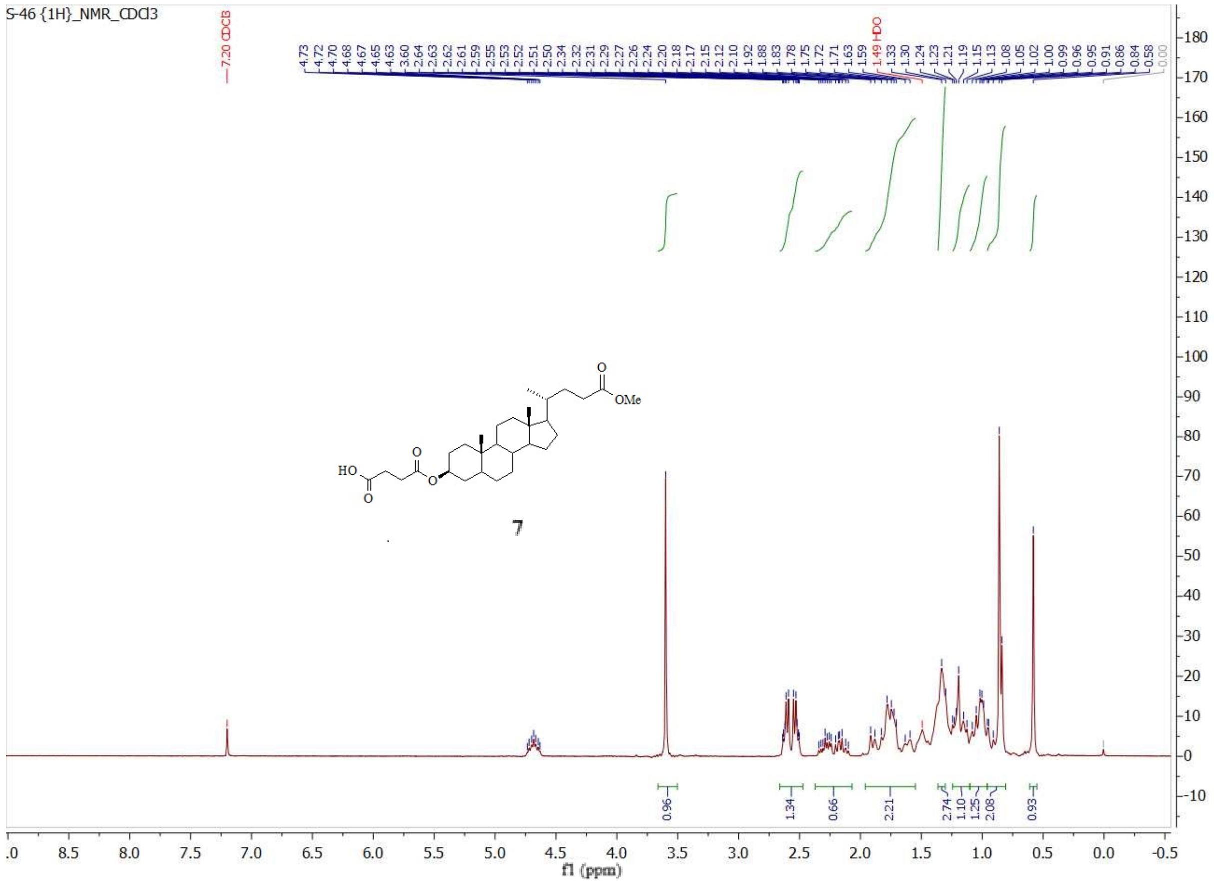

Figure S24 ^{1}H NMR spectra of compound **7.**

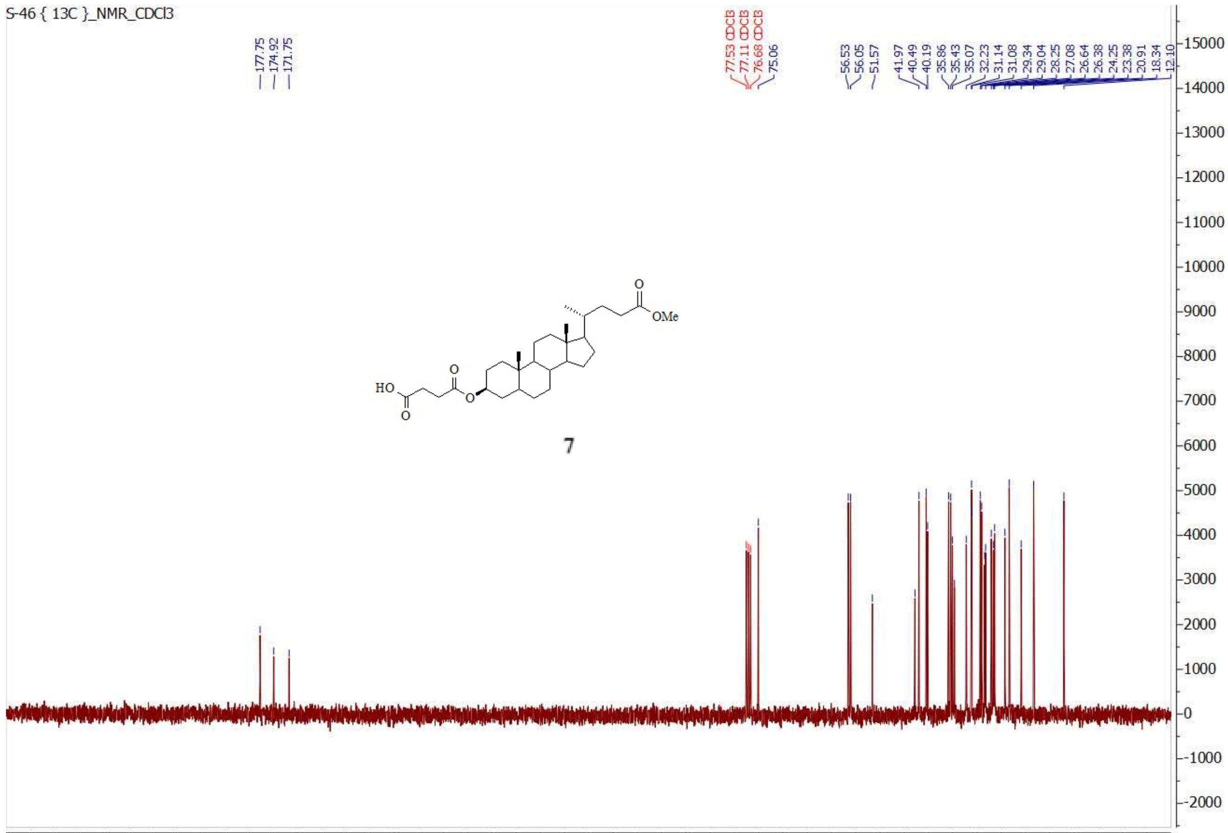

Figure S25 ^{13}C NMR spectra of compound **7.**

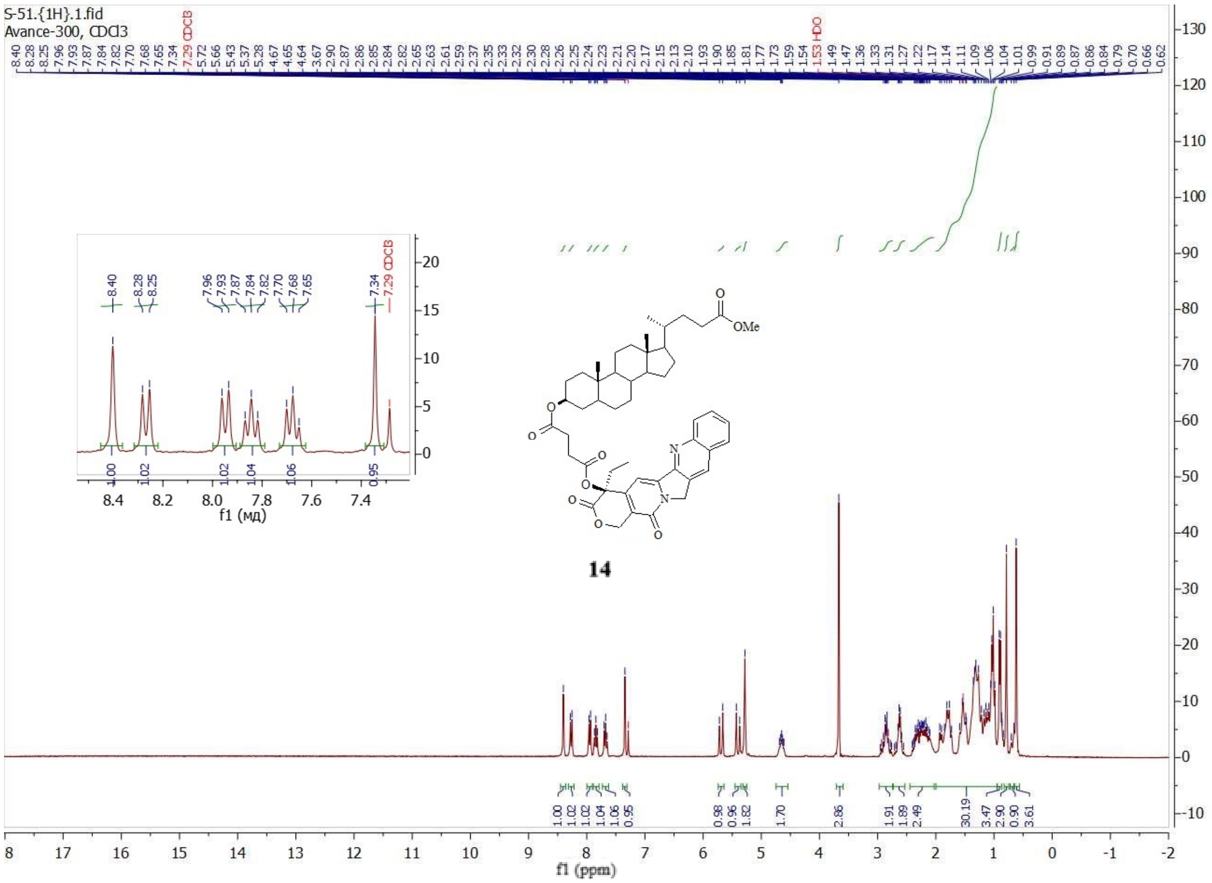

Figure S26 ^{1}H NMR spectra of compound **14.**

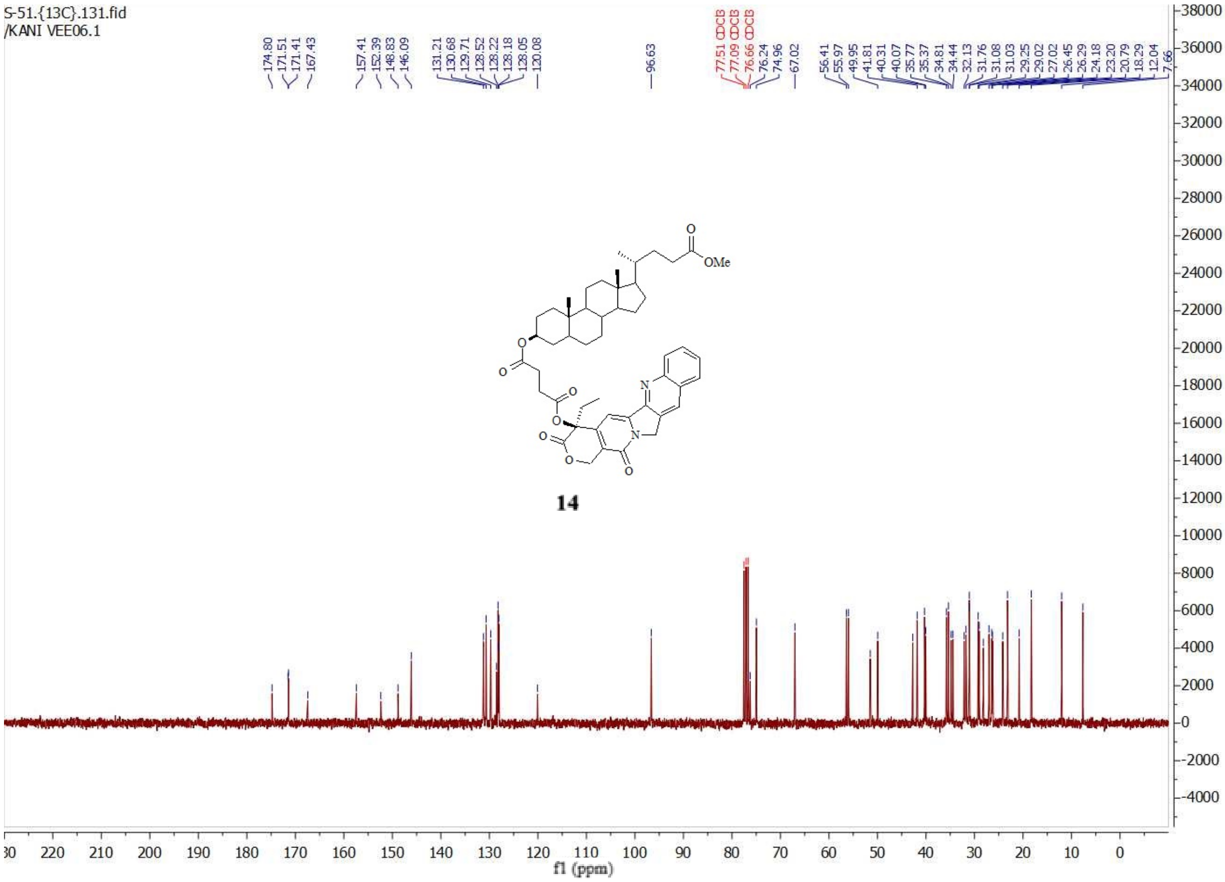

Figure S27 ^{13}C NMR spectra of compound **14.**

Display Report

Analysis Info

Analysis Name	D:\Data\Kolotyrkina\2025\Chobanov\0603018.d	Acquisition Date	03.06.2025 12:35:41
Method	tune_low.m	Operator	BDAL@DE
Sample Name	/ABCD S-51	Instrument / Ser#	micrOTOF 10248
Comment	C49H60N2O9 dlb added CH3OH		

Acquisition Parameter

Source Type	ESI	Ion Polarity	Positive	Set Nebulizer	0.4 Bar
Focus	Not active			Set Dry Heater	180 °C
Scan Begin	50 m/z	Set Capillary	4500 V	Set Dry Gas	4.0 l/min
Scan End	3000 m/z	Set End Plate Offset	-500 V	Set Divert Valve	Waste

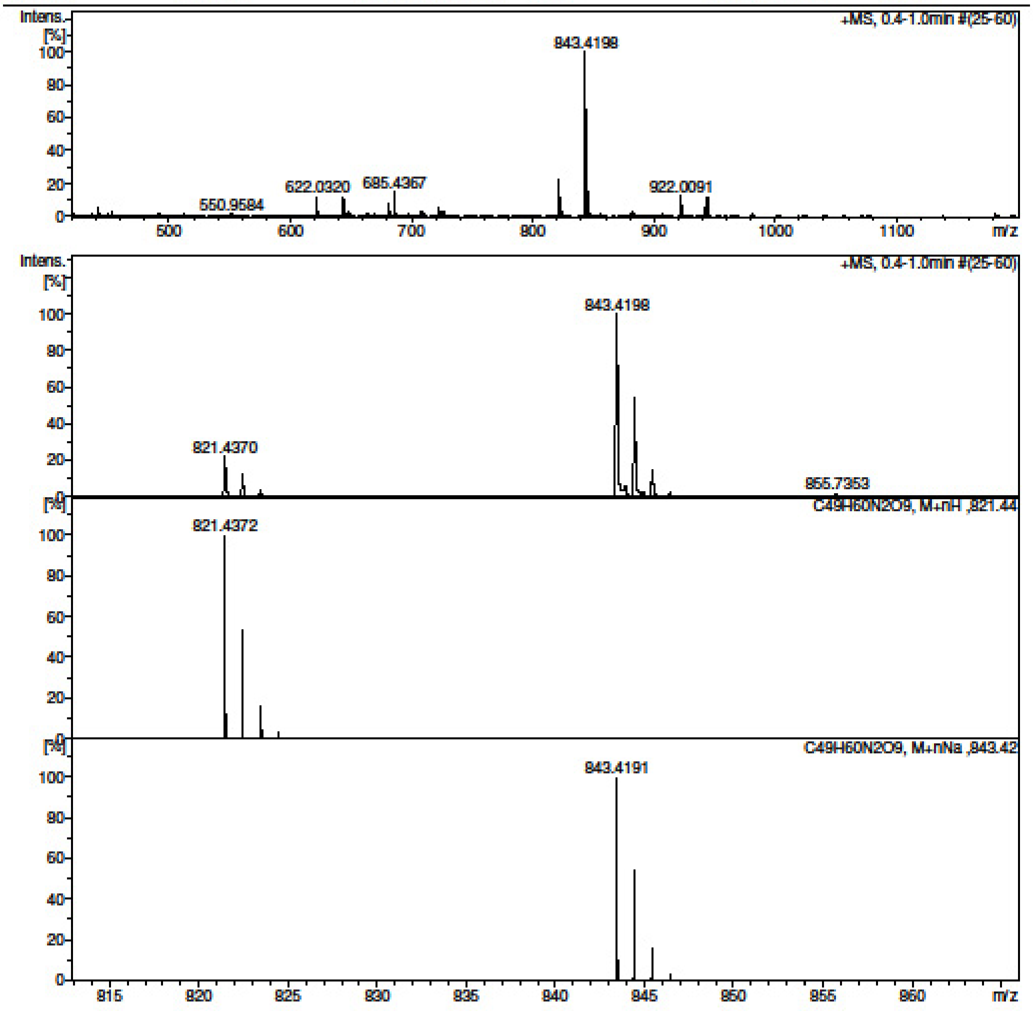

Figure S28 HRMS/MS spectra of compound **14.**

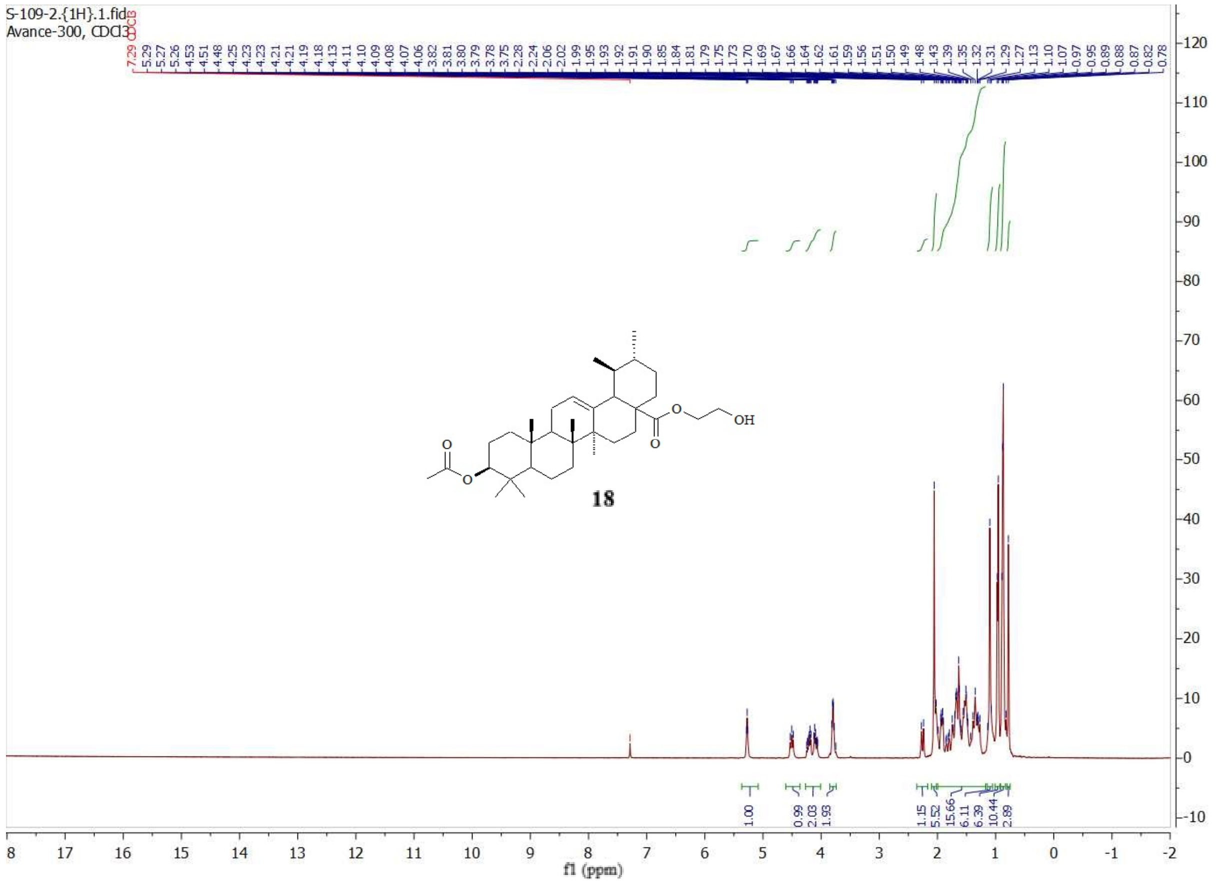

Figure S29 ^{1}H NMR spectra of compound **18.**

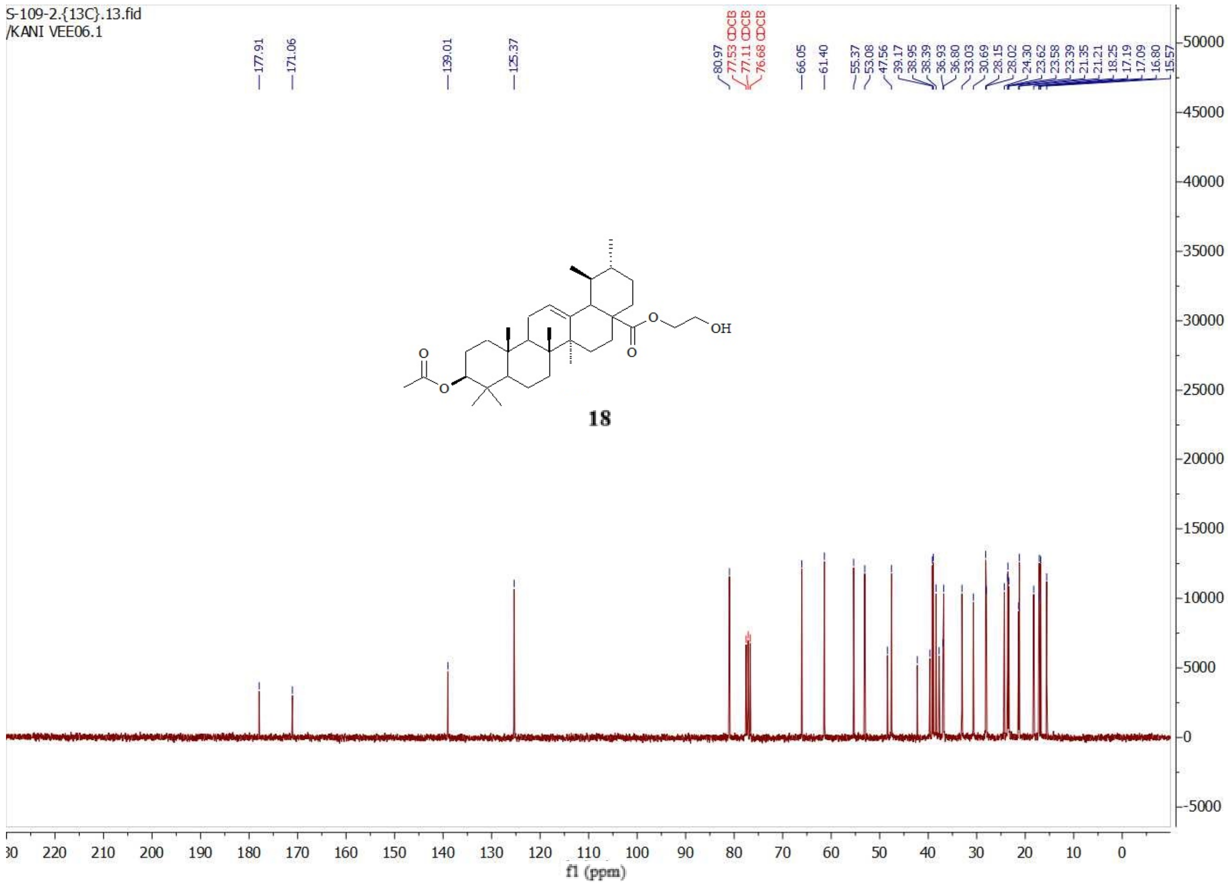

Figure S30 ^{13}C NMR spectra of compound **18.**

Display Report

Analysis Info

Analysis Name	D:\Data\Kolotyrkina\2025\Chobanov\0604015.d	Acquisition Date	04.06.2025 12:46:15
Method	tune_low.m	Operator	BDAL@DE
Sample Name	/ABCD S-109-2	Instrument / Ser#	micrOTOF 10248
Comment	C34H54O5 clb added CH3OH		

Acquisition Parameter

Source Type	ESI	Ion Polarity	Positive			Set Nebulizer	0.4 Bar
Focus	Not active					Set Dry Heater	180 °C
Scan Begin	50 m/z	Set Capillary	4500 V			Set Dry Gas	4.0 l/min
Scan End	3000 m/z	Set End Plate Offset	-500 V			Set Divert Valve	Waste

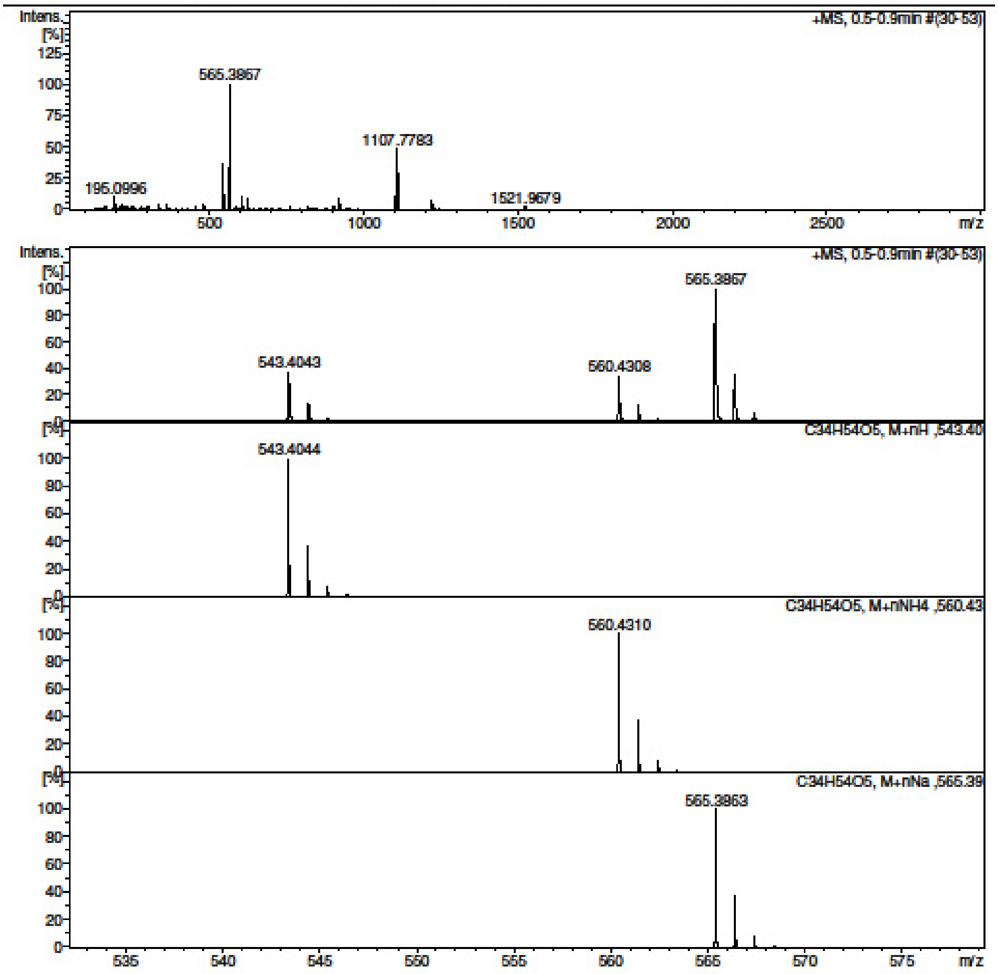

Figure S31 HRMS/MS spectra of compound **18.**

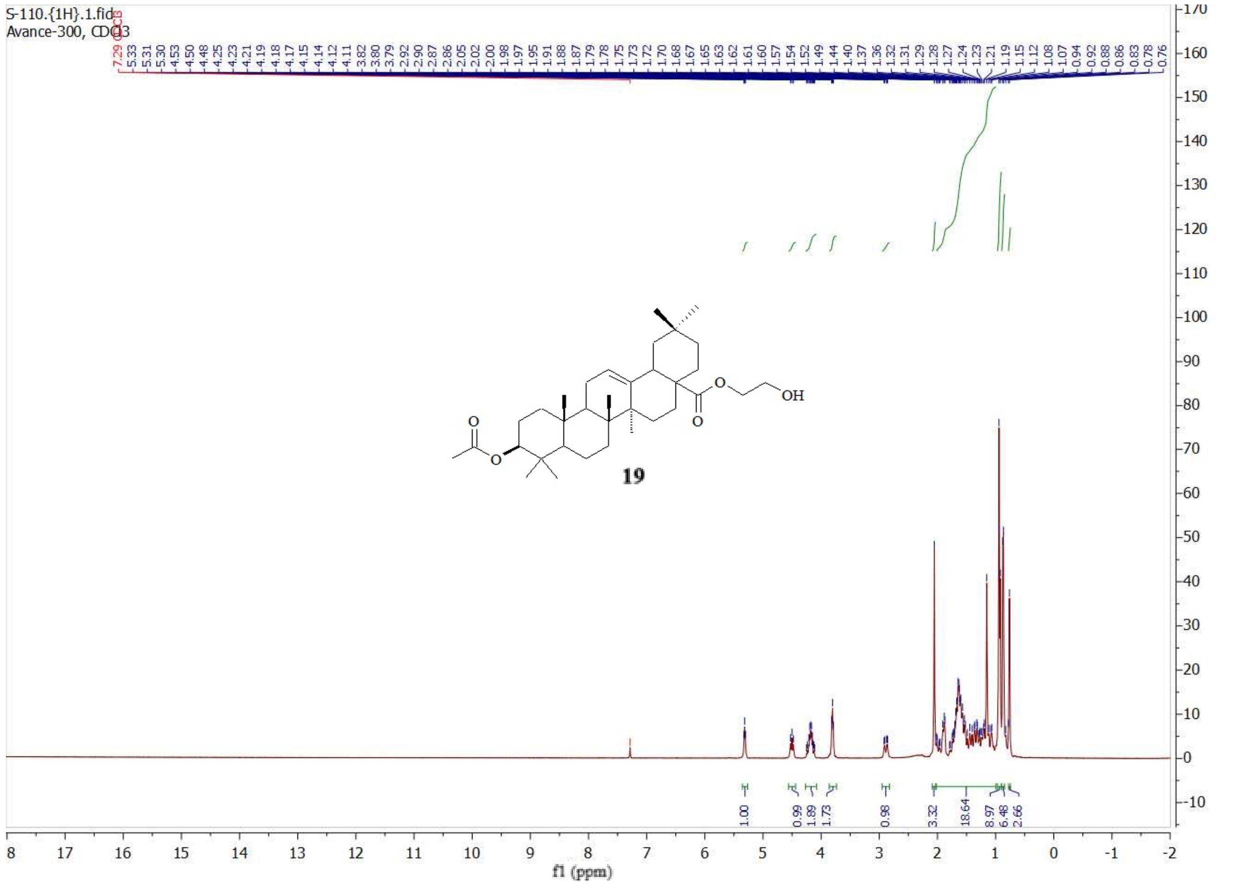

Figure S32 ^{1}H NMR spectra of compound **19.**

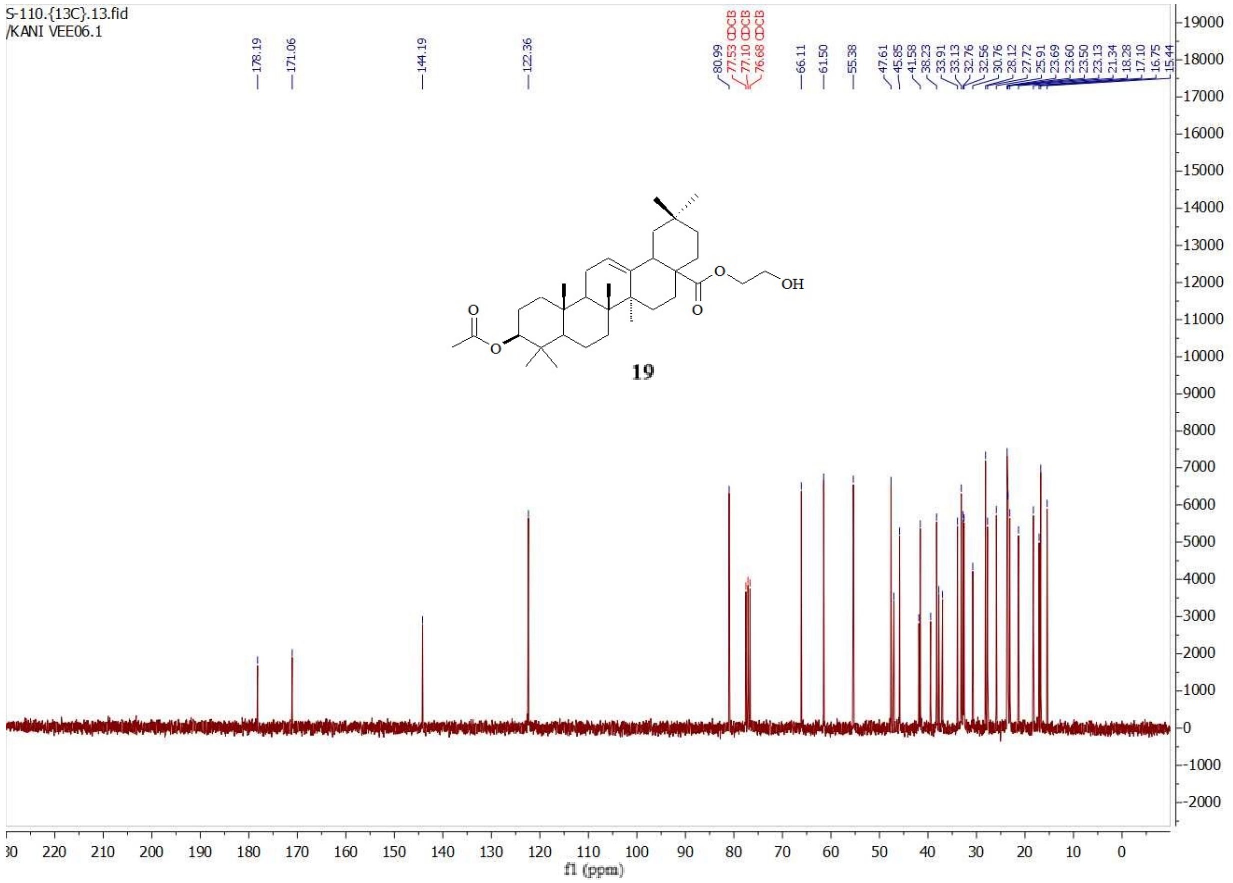

Figure S33 ^{13}C NMR spectra of compound **19.**

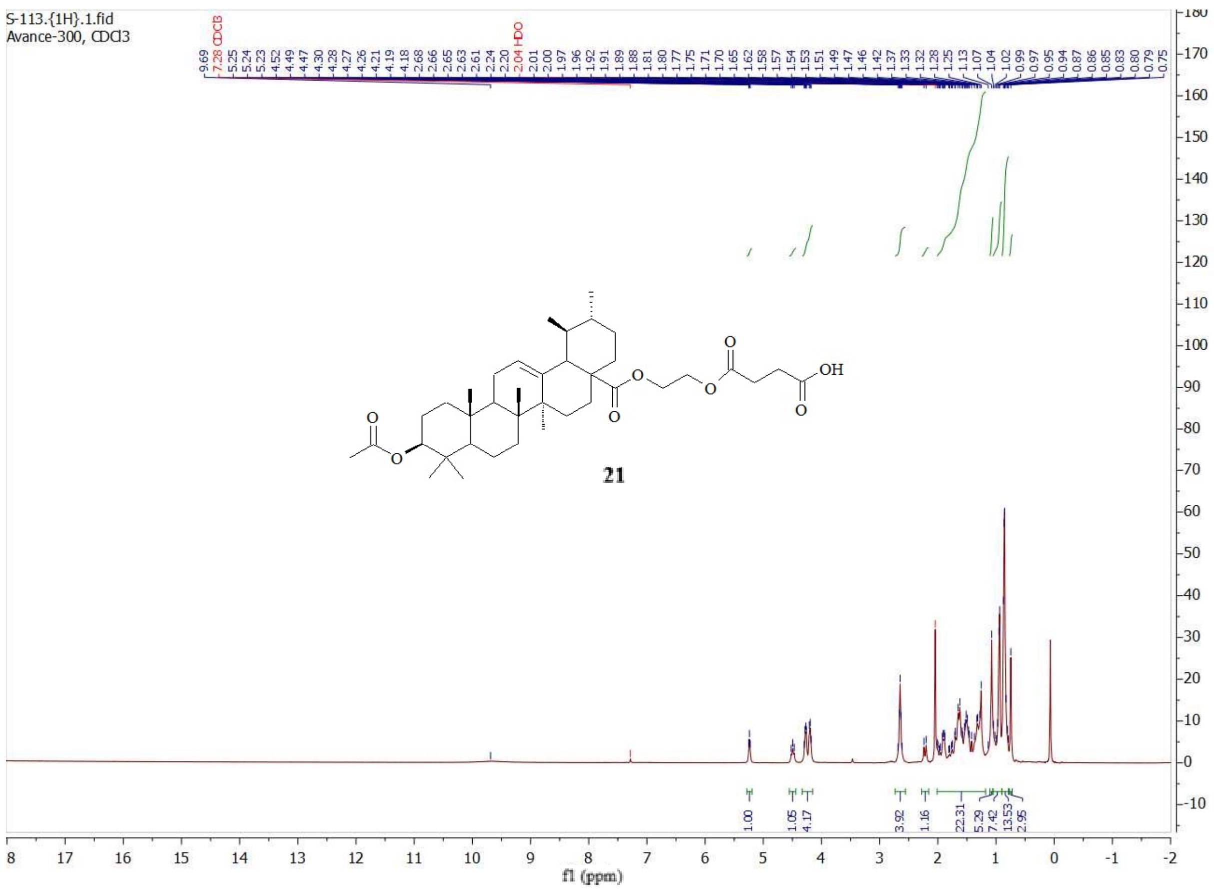

Figure S34 ^{1}H NMR spectra of compound **21.**

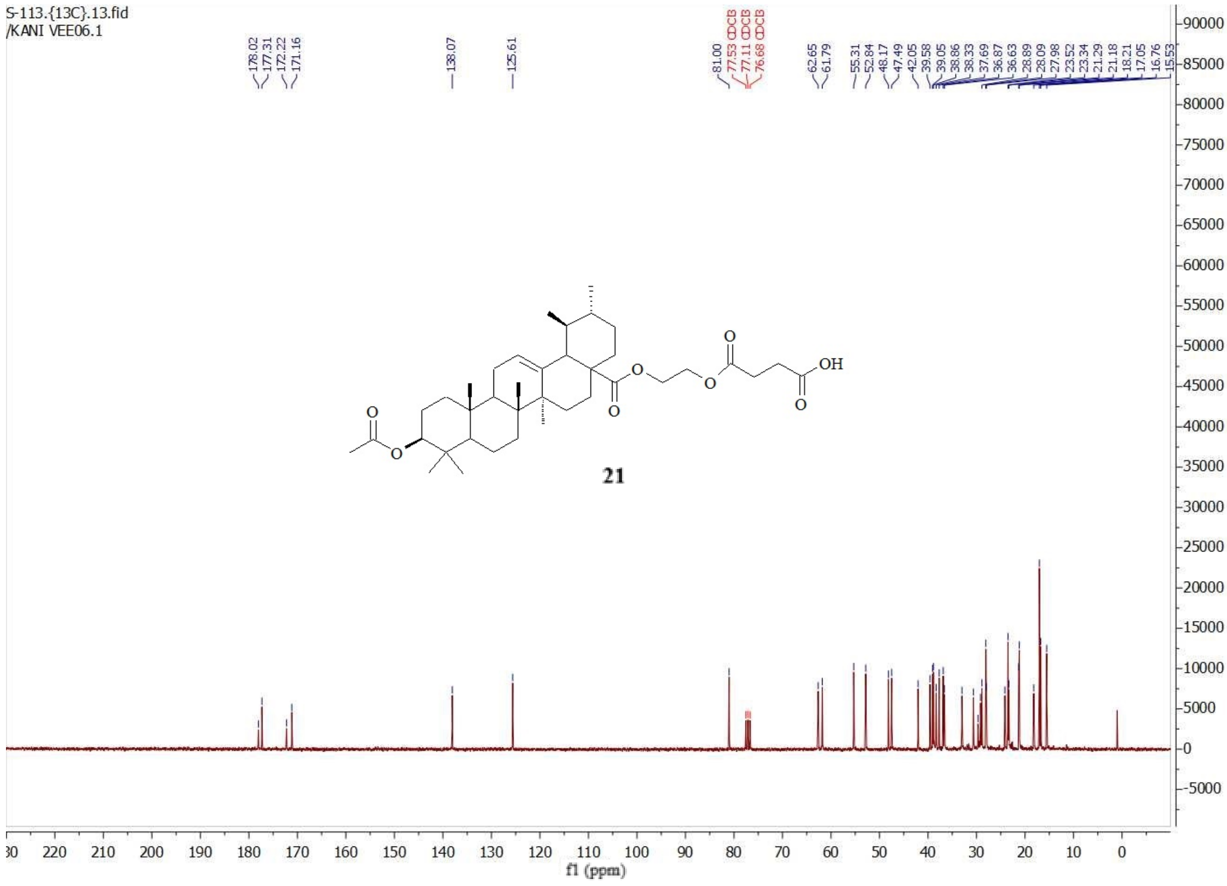

Figure S35 ^{13}C NMR spectra of compound **21.**

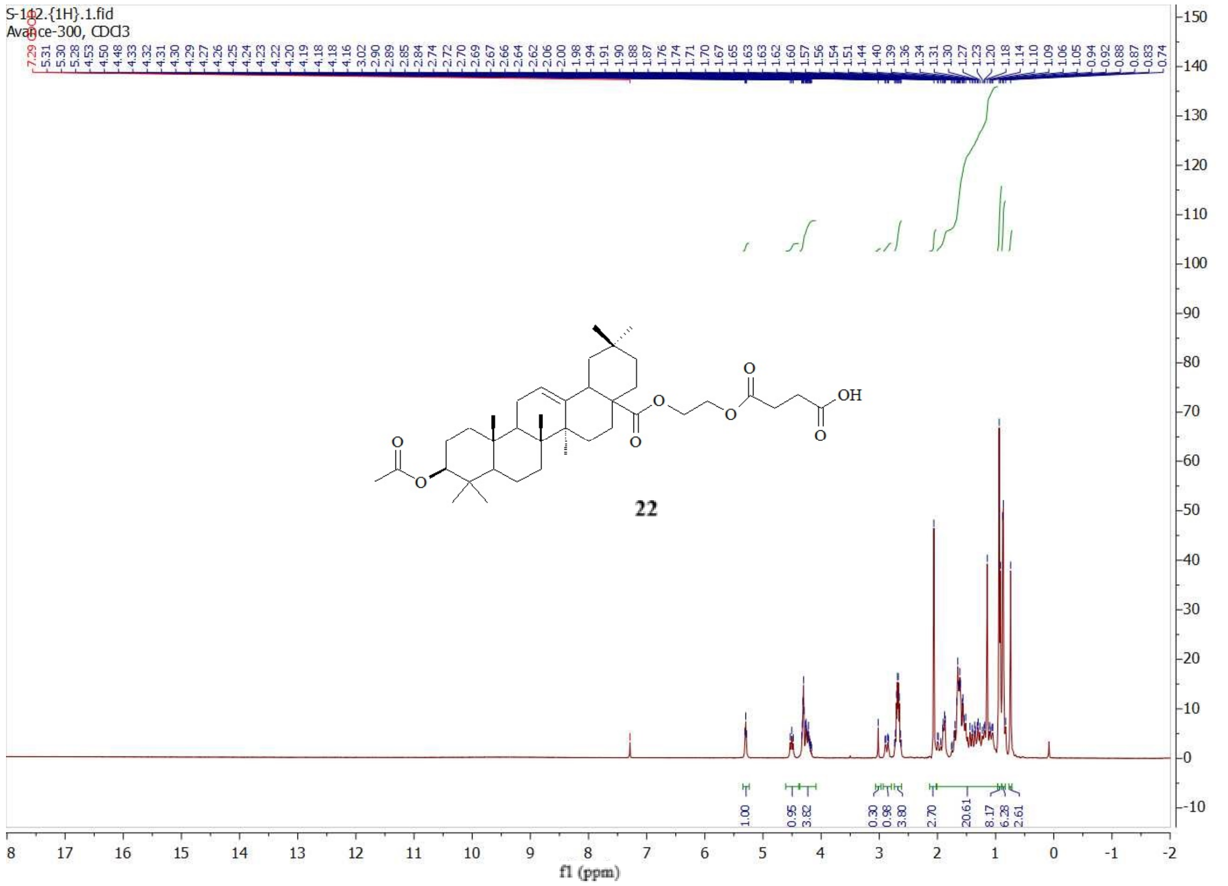

Figure S36 ^{1}H NMR spectra of compound **22.**

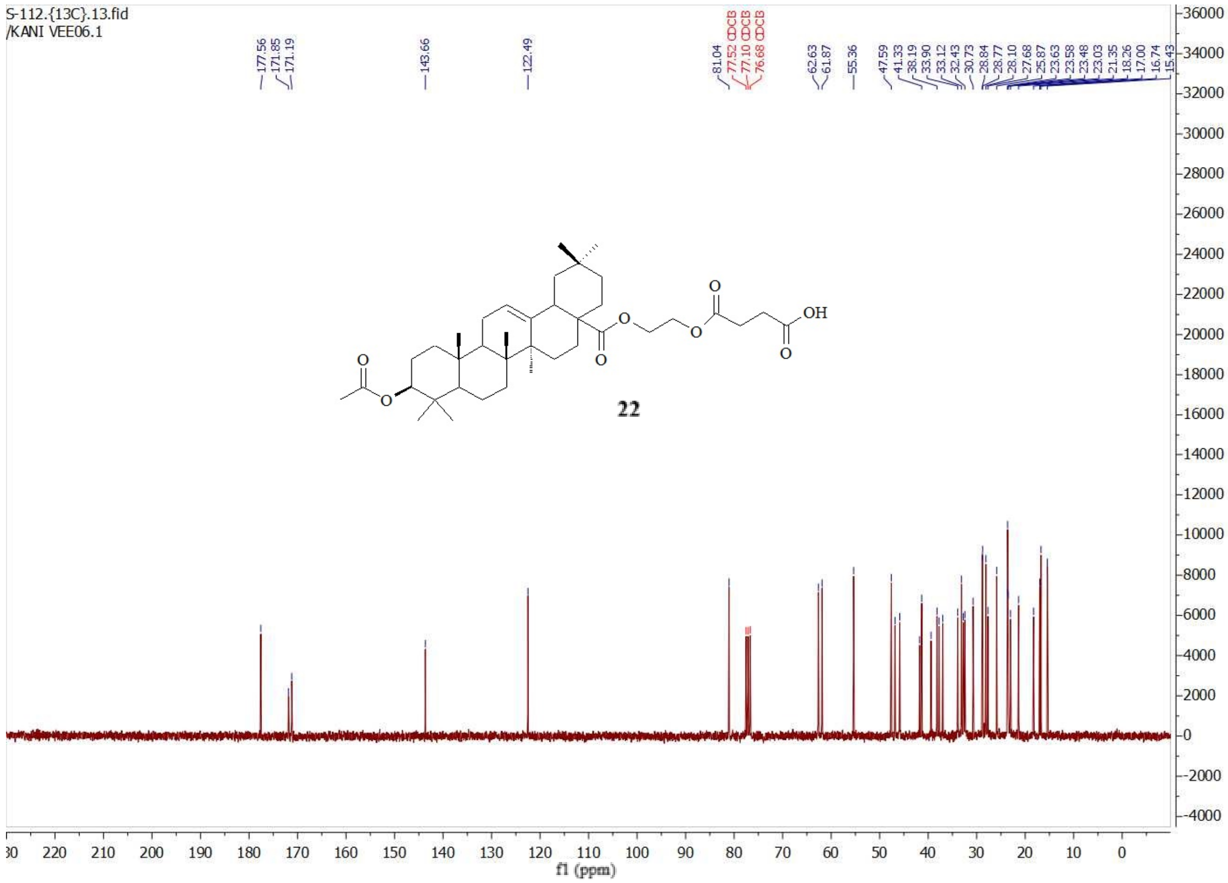

Figure S37 ^{13}C NMR spectra of compound **22.**

Display Report

Analysis Info

Analysis Name	D:\Data\Kolotyrkina\2025\Chobanov\0604016.d	Acquisition Date	04.06.2025 12:56:15
Method	tune_low.m	Operator	BDAL@DE
Sample Name	/ABCD S-112	Instrument / Ser#	micrOTOF 10248
Comment	C38H58O8 clb added CH3OH		

Acquisition Parameter

Source Type	ESI	Ion Polarity	Positive	Set Nebulizer	0.4 Bar
Focus	Not active			Set Dry Heater	180 °C
Scan Begin	50 m/z	Set Capillary	4500 V	Set Dry Gas	4.0 l/min
Scan End	3000 m/z	Set End Plate Offset	-500 V	Set Divert Valve	Waste

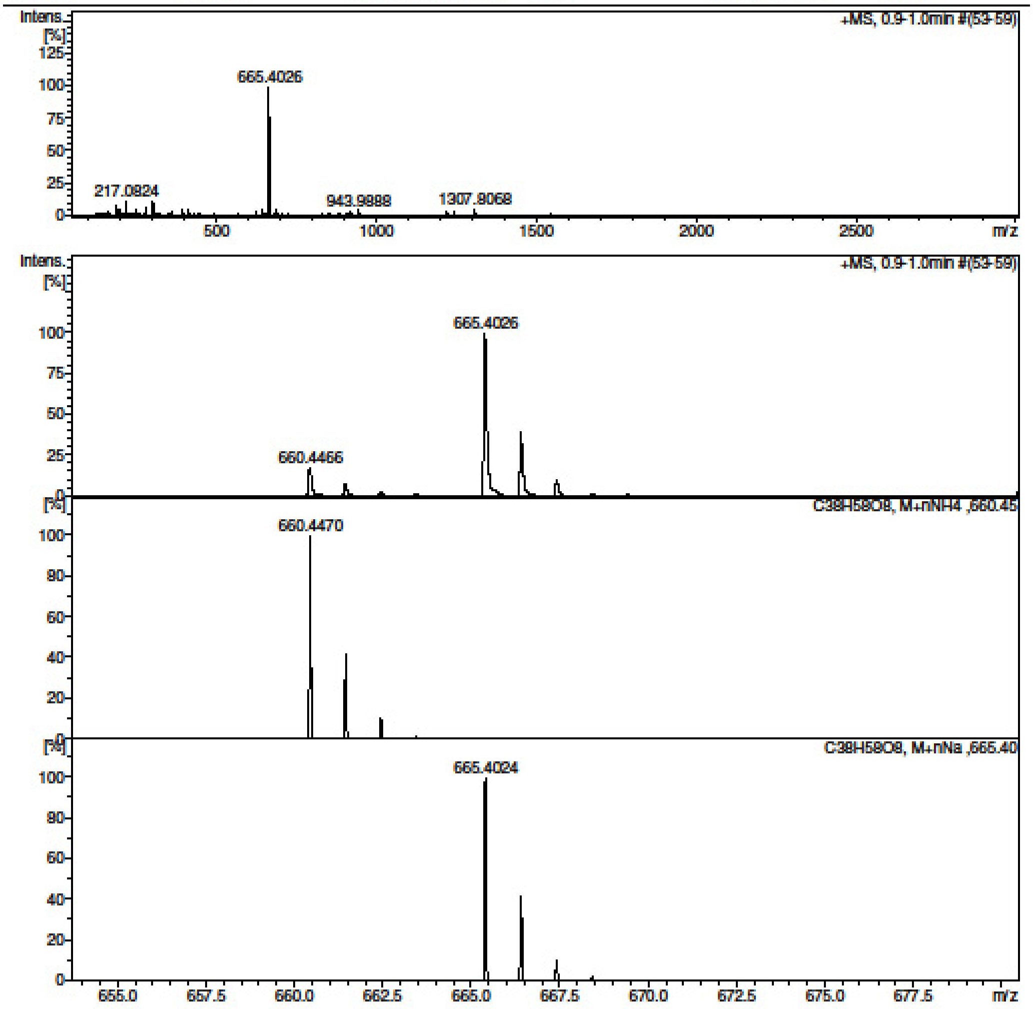

Bruker Compass DataAnalysis 4.0 printed: 04.06.2025 13:05:00 Page 1 of 1

Figure S38 HRMS/MS spectra of compound **22.**

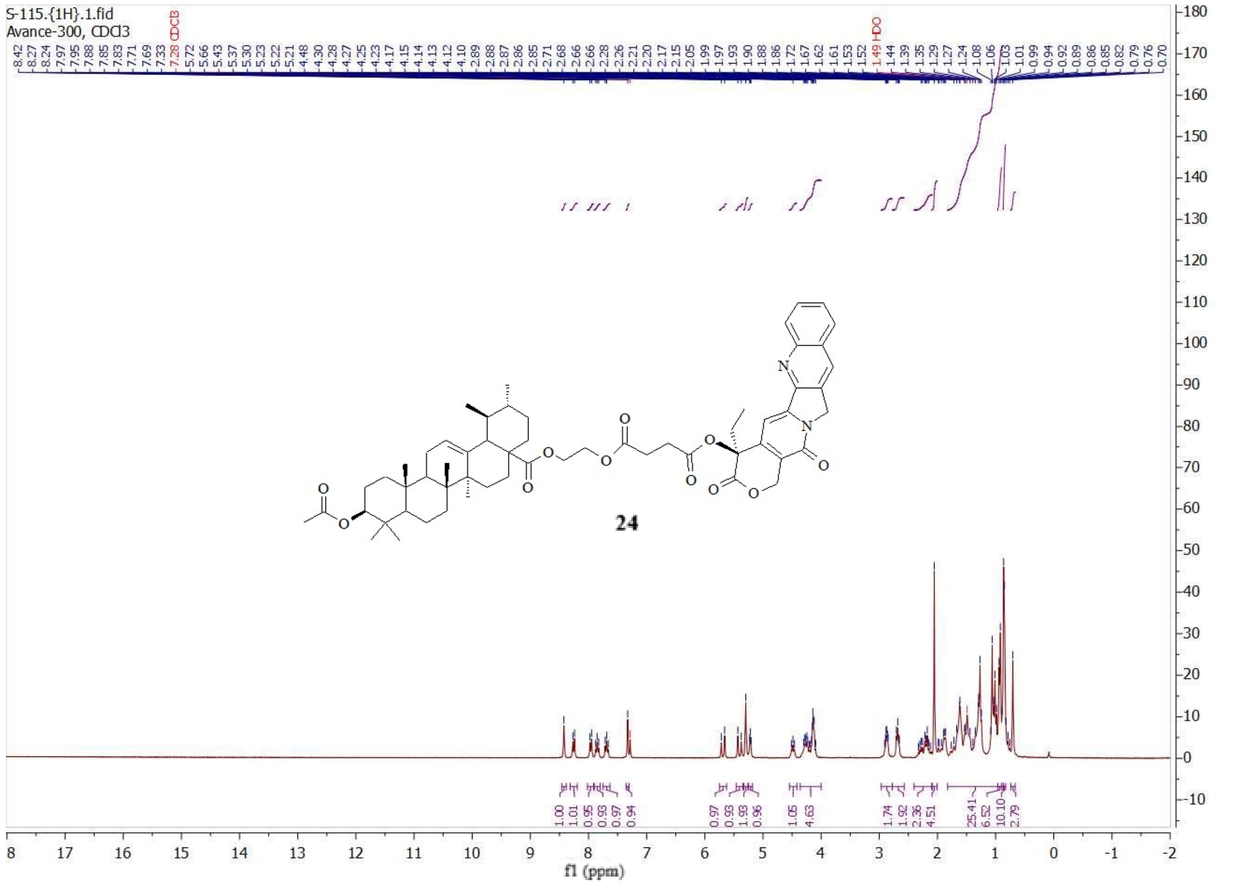

Figure S39 ^{1}H NMR spectra of compound **24.**

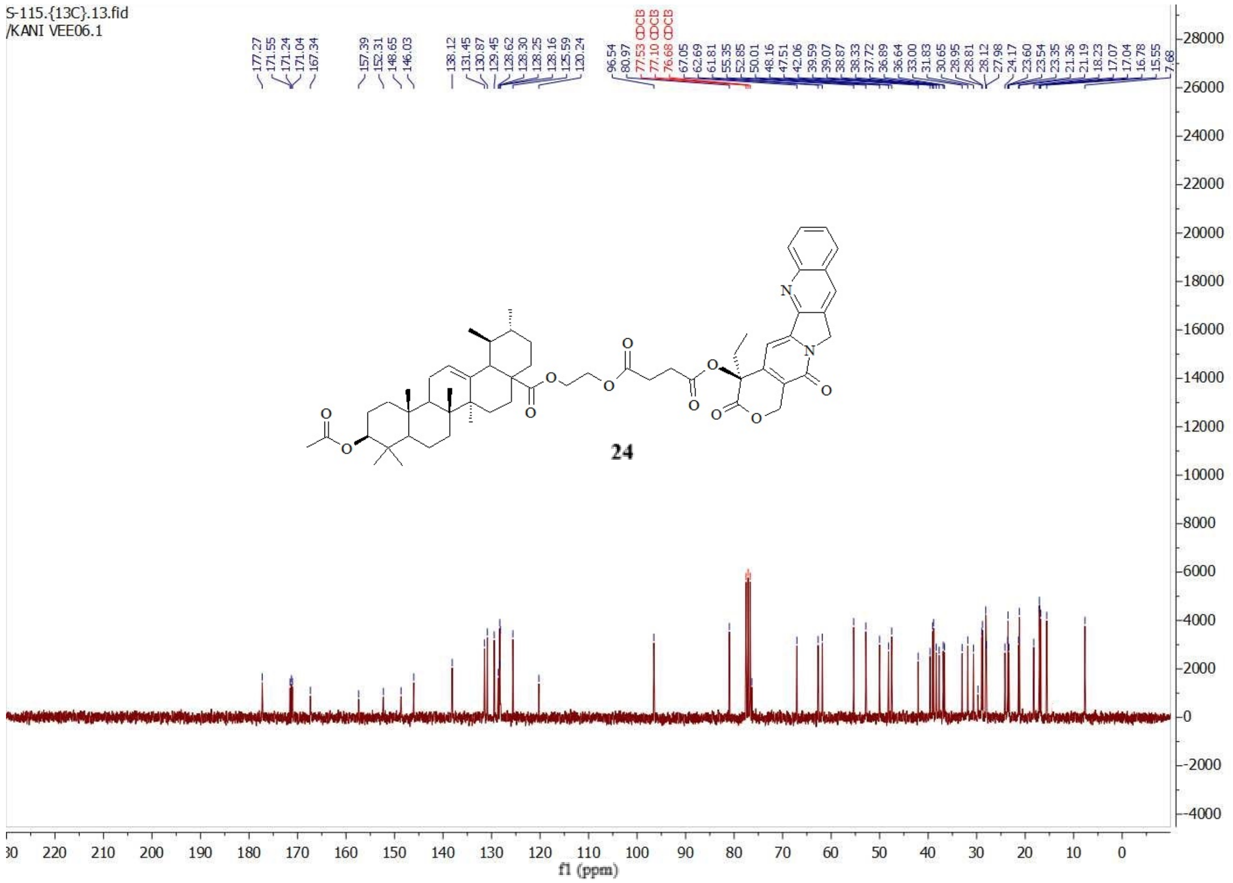

Figure S40 ^{13}C NMR spectra of compound **24.**

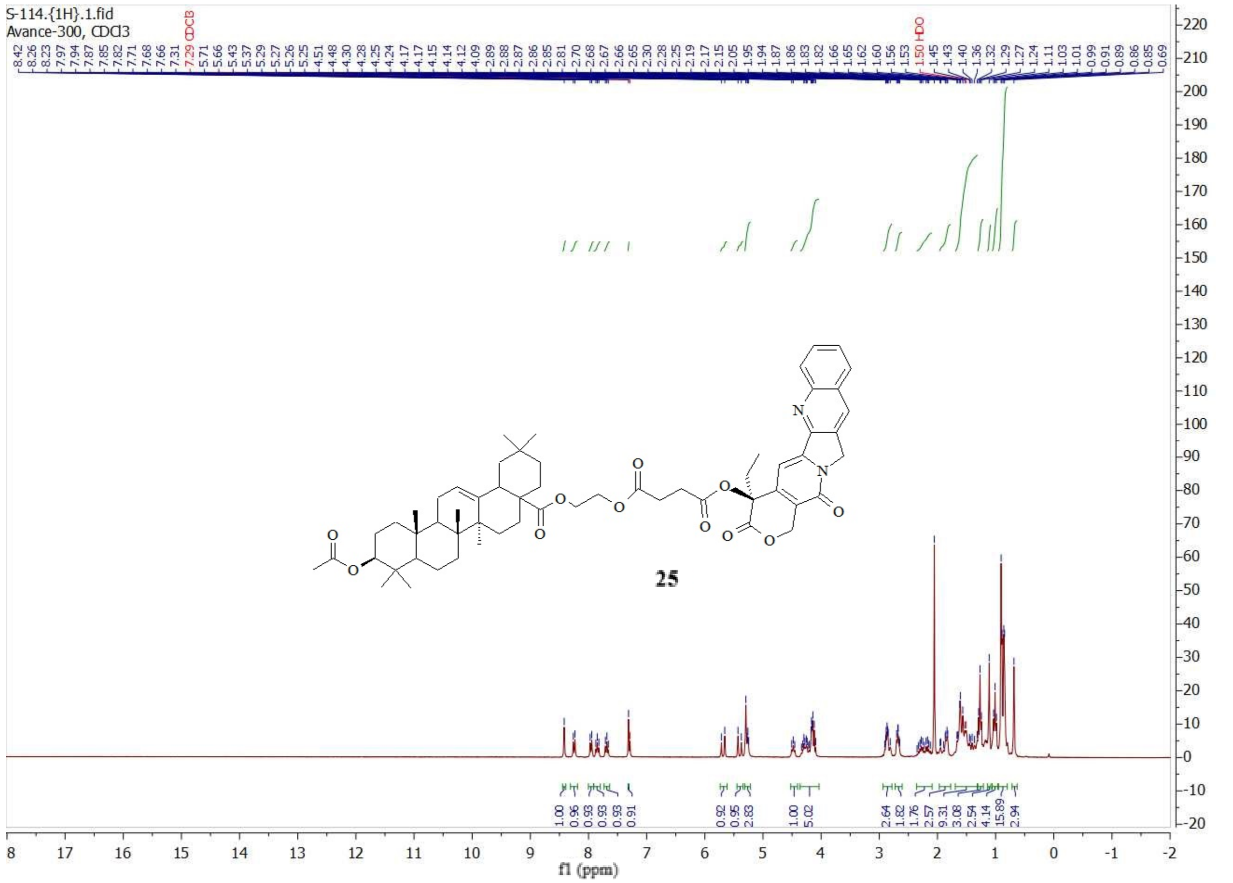

Figure S41 ^{1}H NMR spectra of compound **25.**

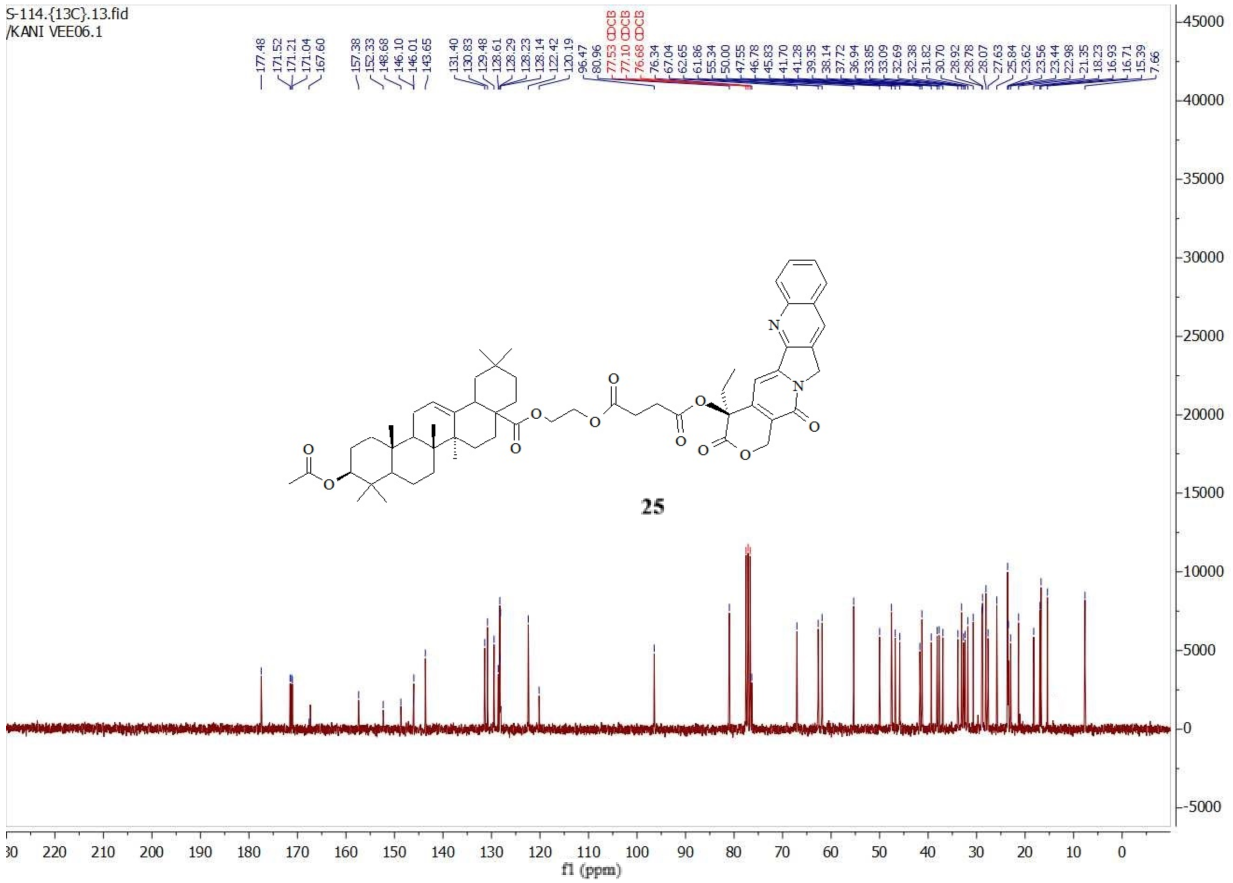

Figure S42 ^{13}C NMR spectra of compound **25.**

Display Report

Analysis Info

Analysis Name	D:\Data\Kolotyrkina\2025\Chobanov\0603030.d	Acquisition Date	03.06.2025 15:53:40
Method	tune_low.m	Operator	BDAL@DE
Sample Name	/ABCD S-114	Instrument / Ser#	micrOTOF 10248
Comment	C58H72N2O11 clb added CH3OH		

Acquisition Parameter

Source Type	ESI	Ion Polarity	Positive	Set Nebulizer	0.4 Bar
Focus	Not active			Set Dry Heater	180 °C
Scan Begin	50 m/z	Set Capillary	4500 V	Set Dry Gas	4.0 l/min
Scan End	3000 m/z	Set End Plate Offset	-500 V	Set Divert Valve	Waste

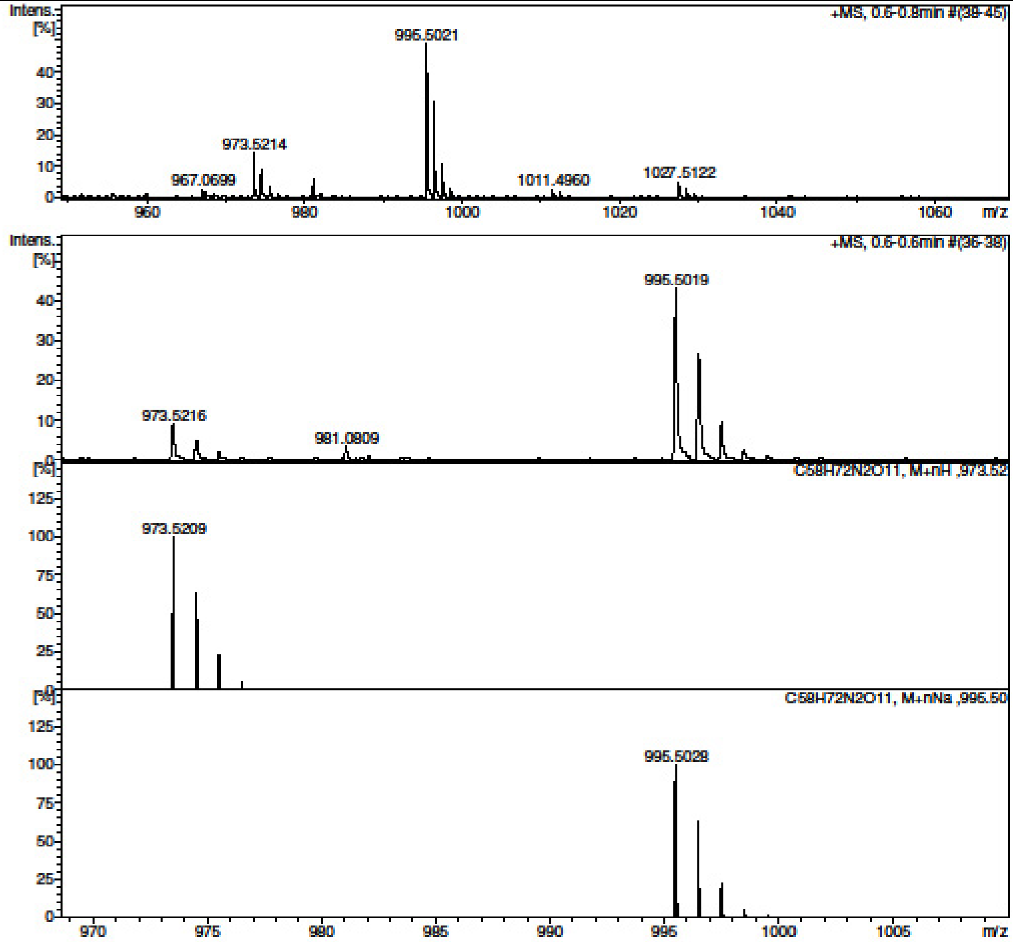

Figure S43 HRMS/MS spectra of compound **25.**

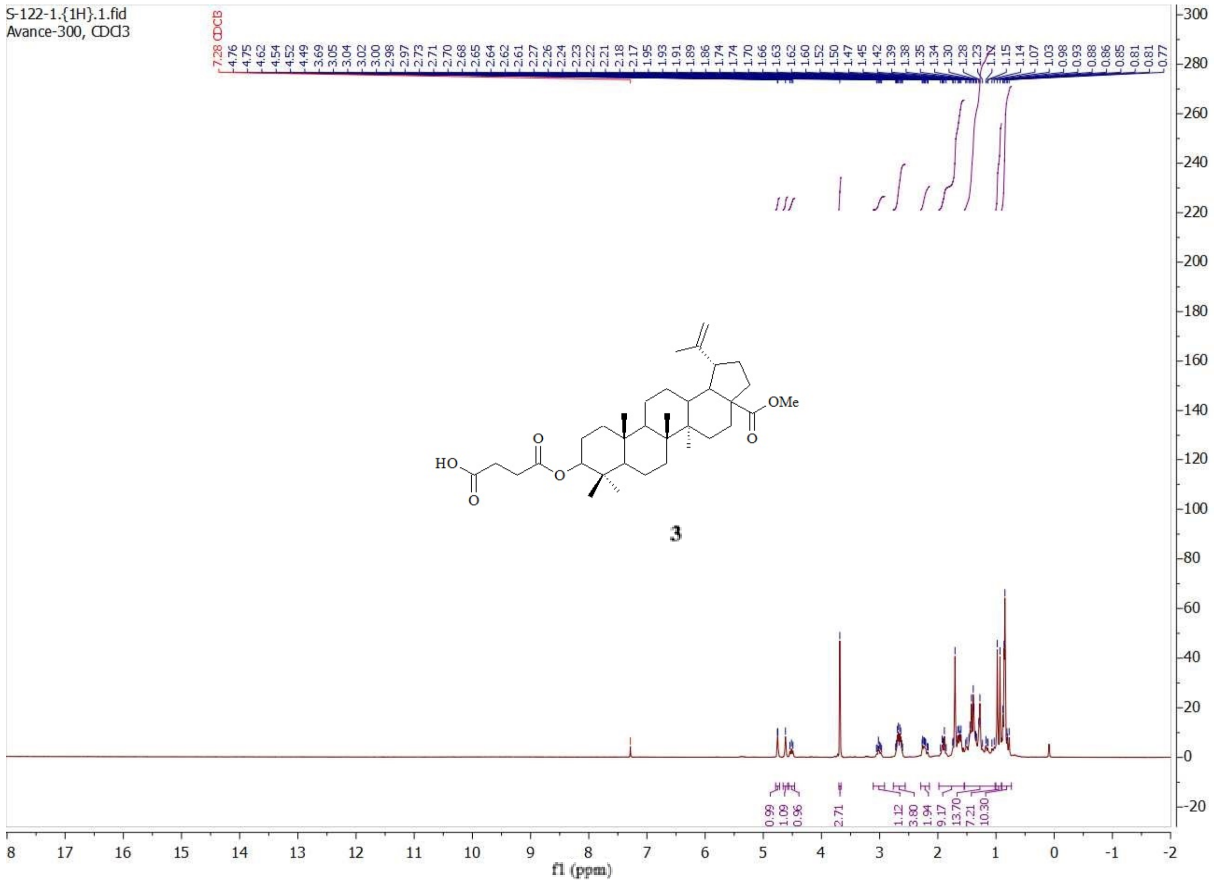

Figure S44 ^{1}H NMR spectra of compound **3.**

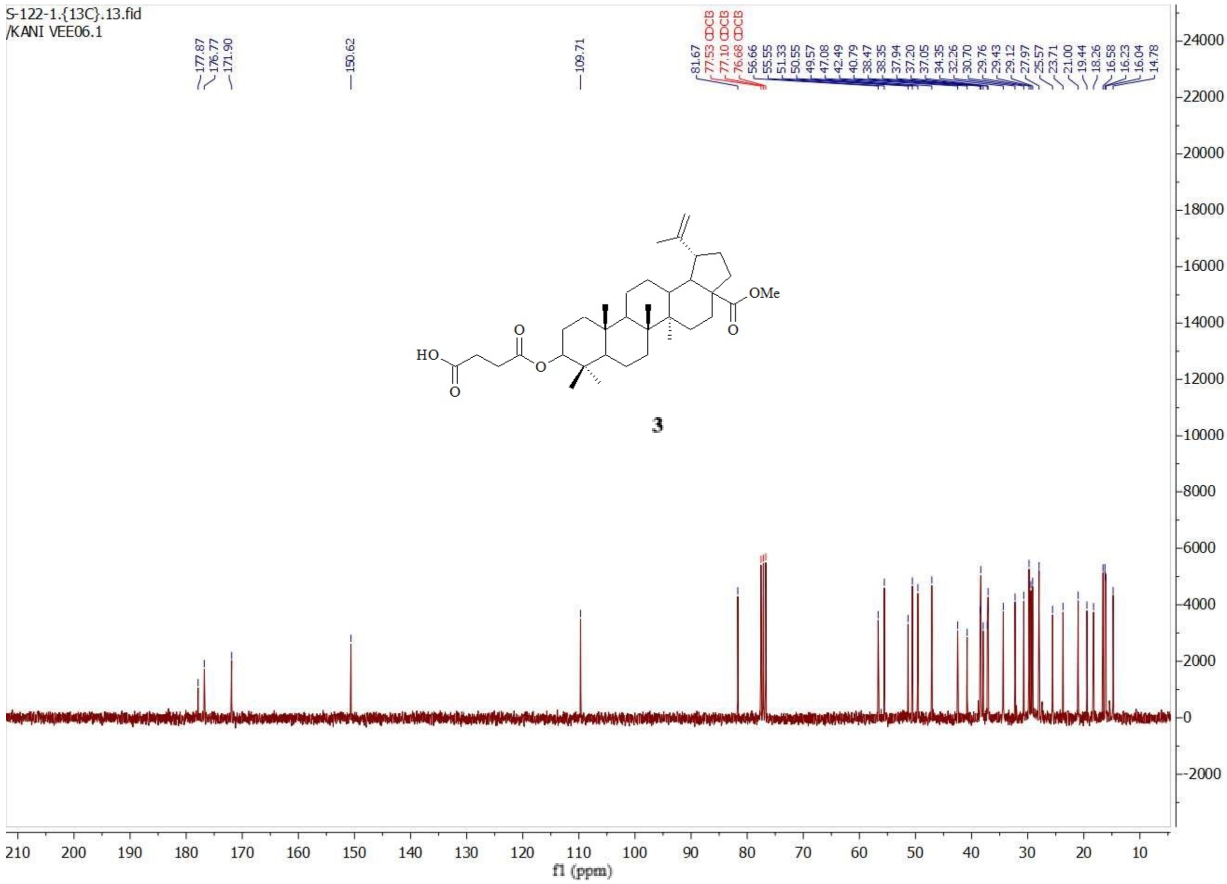

Figure S45 ^{13}C NMR spectra of compound **3.**

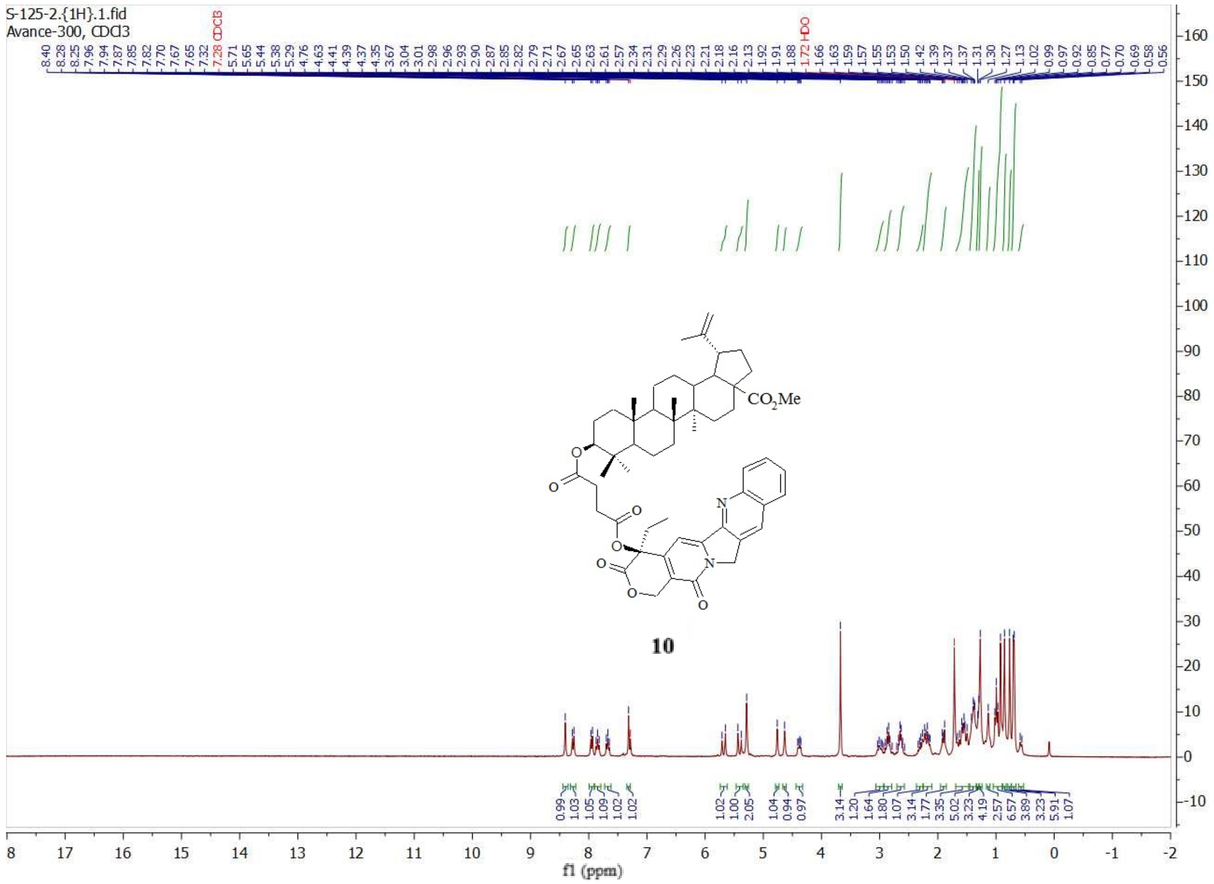

Figure S46 ^{1}H NMR spectra of compound **10.**

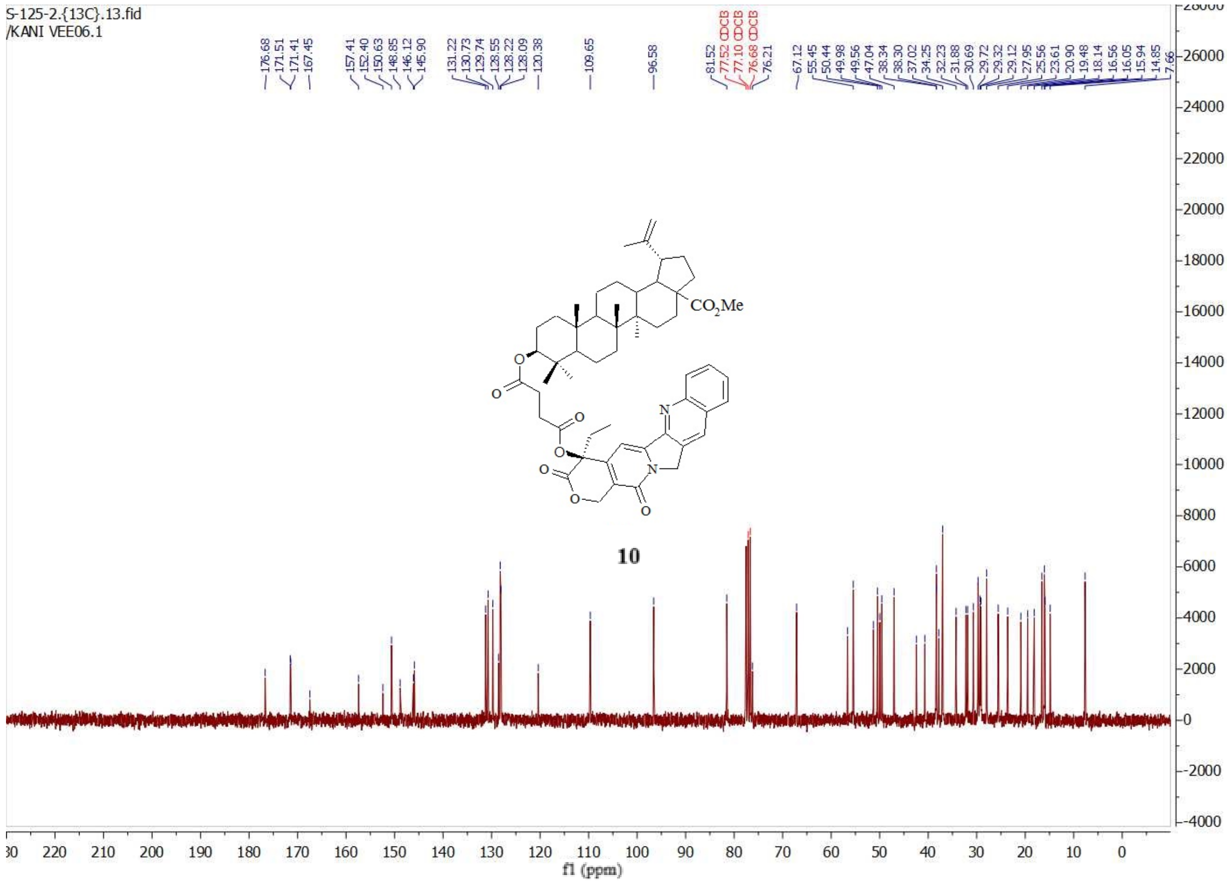

Figure S47 ^{13}C NMR spectra of compound **10.**

Display Report

Analysis Info

Analysis Name	D:\Data\Kolotyrkina\2025\Chobanov\0603028.d	Acquisition Date	03.06.2025 15:43:44
Method	tune_low.m	Operator	BDAL@DE
Sample Name	/ABCD S-125-2	Instrument / Ser#	micrOTOF 10248
Comment	C55H68N2O9 dlb added CH3OH		

Acquisition Parameter

Source Type	ESI	Ion Polarity	Positive	Set Nebulizer	0.4 Bar
Focus	Not active			Set Dry Heater	180 °C
Scan Begin	50 m/z	Set Capillary	4500 V	Set Dry Gas	4.0 l/min
Scan End	3000 m/z	Set End Plate Offset	-500 V	Set Divert Valve	Waste

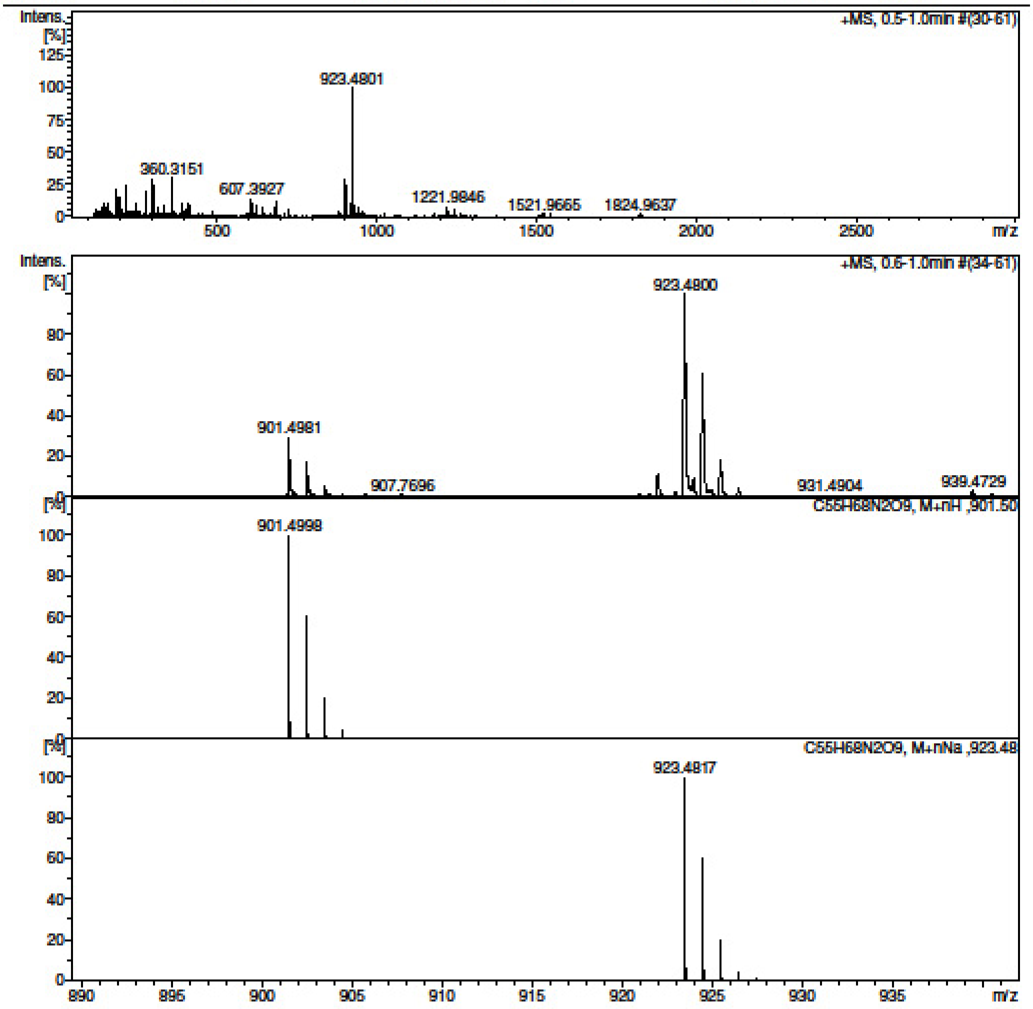

Figure S48 HRMS/MS spectra of compound **10.**

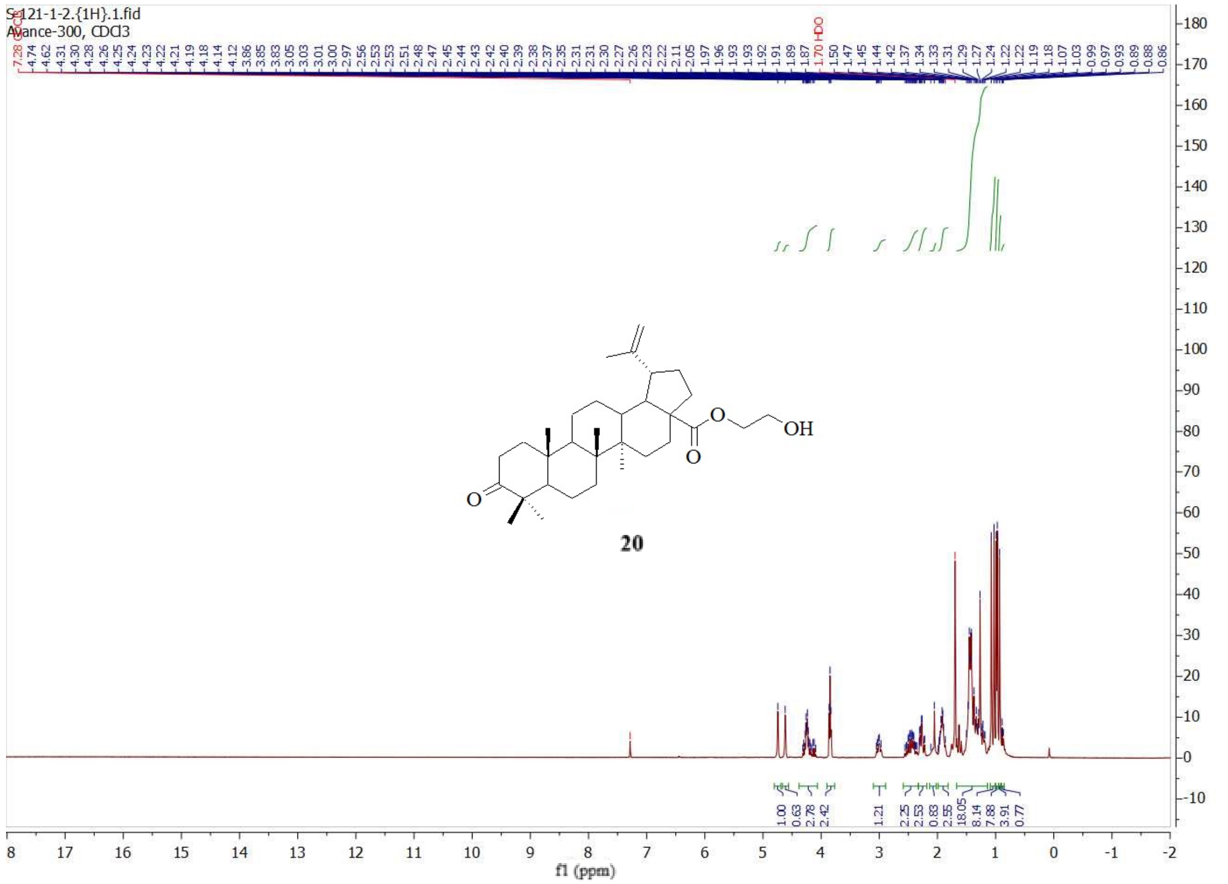

Figure S49 ^{1}H NMR spectra of compound **20.**

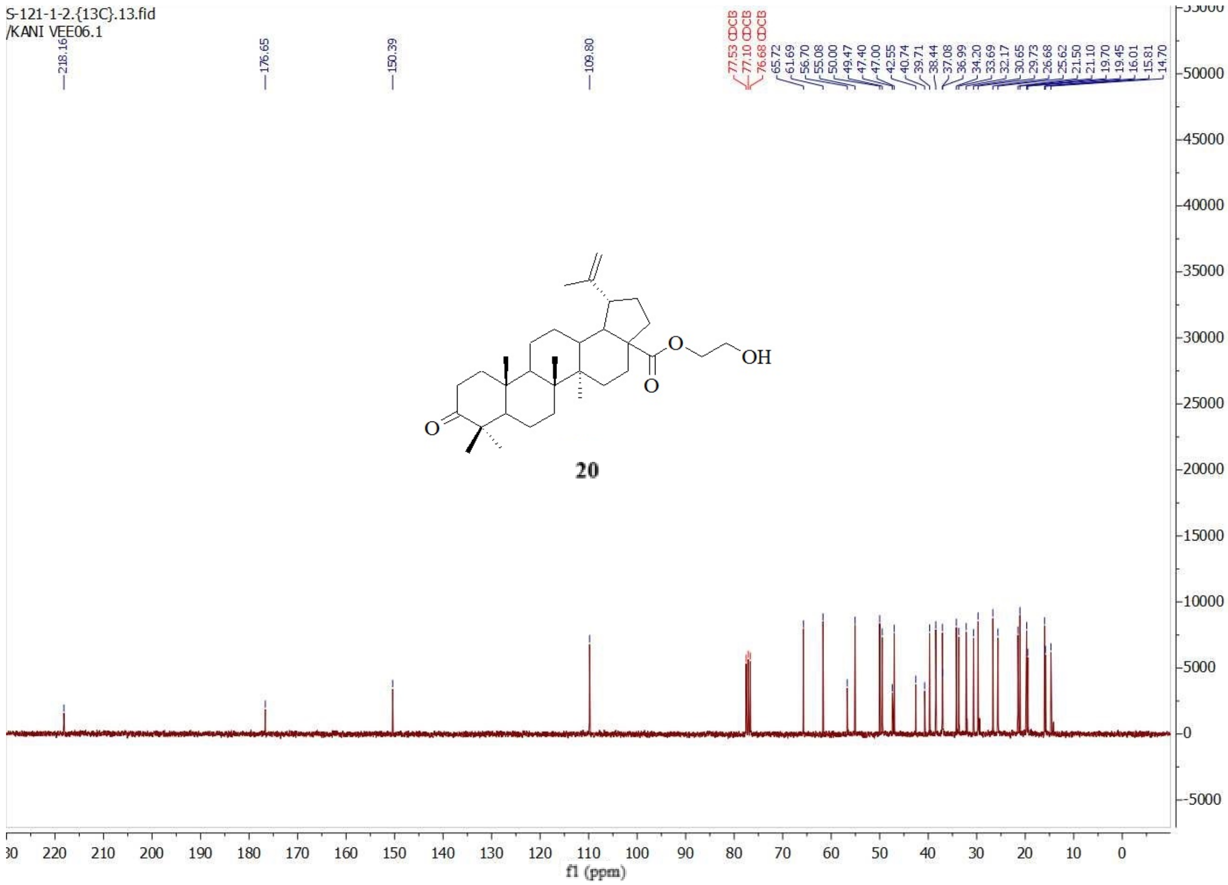

Figure S50 ^{13}C NMR spectra of compound **20.**

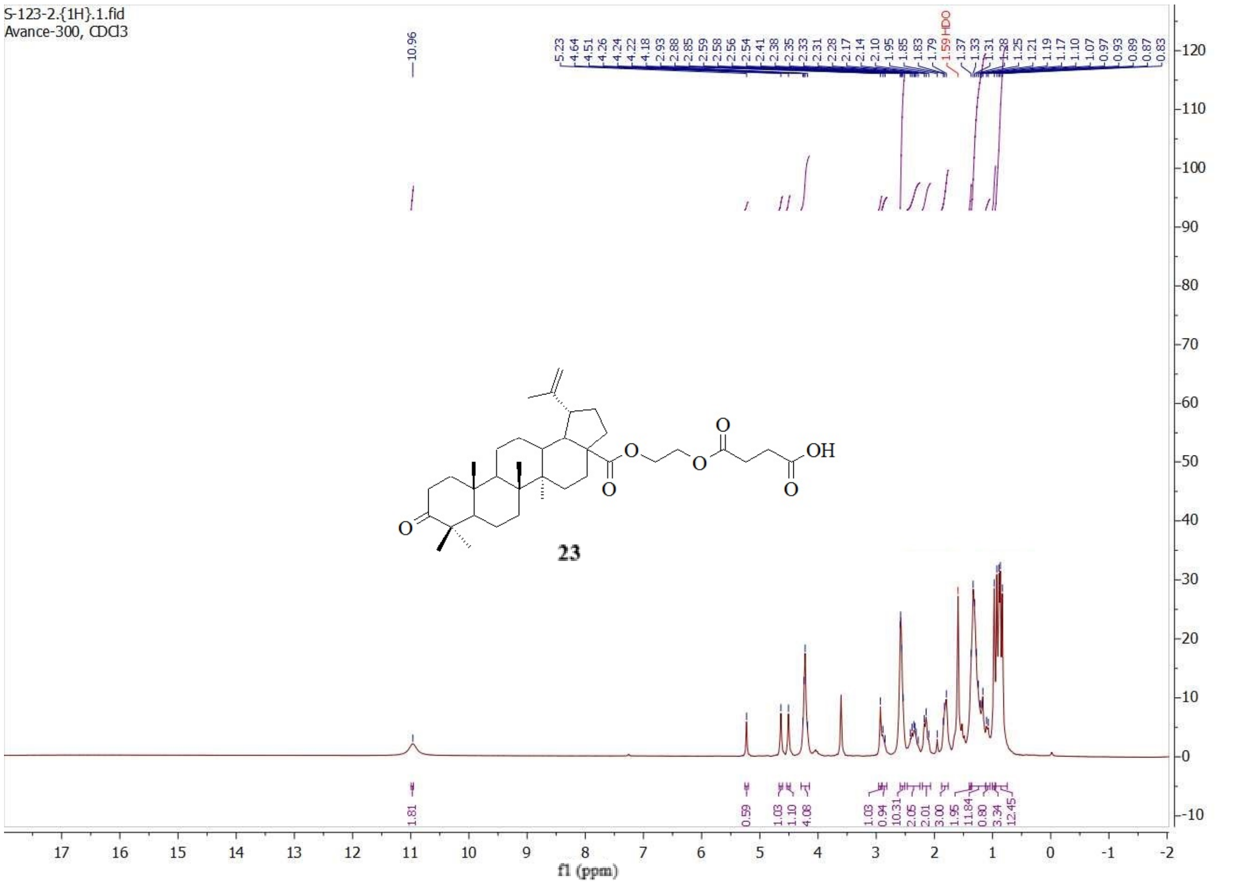

Figure S51 ^{1}H NMR spectra of compound **23.**

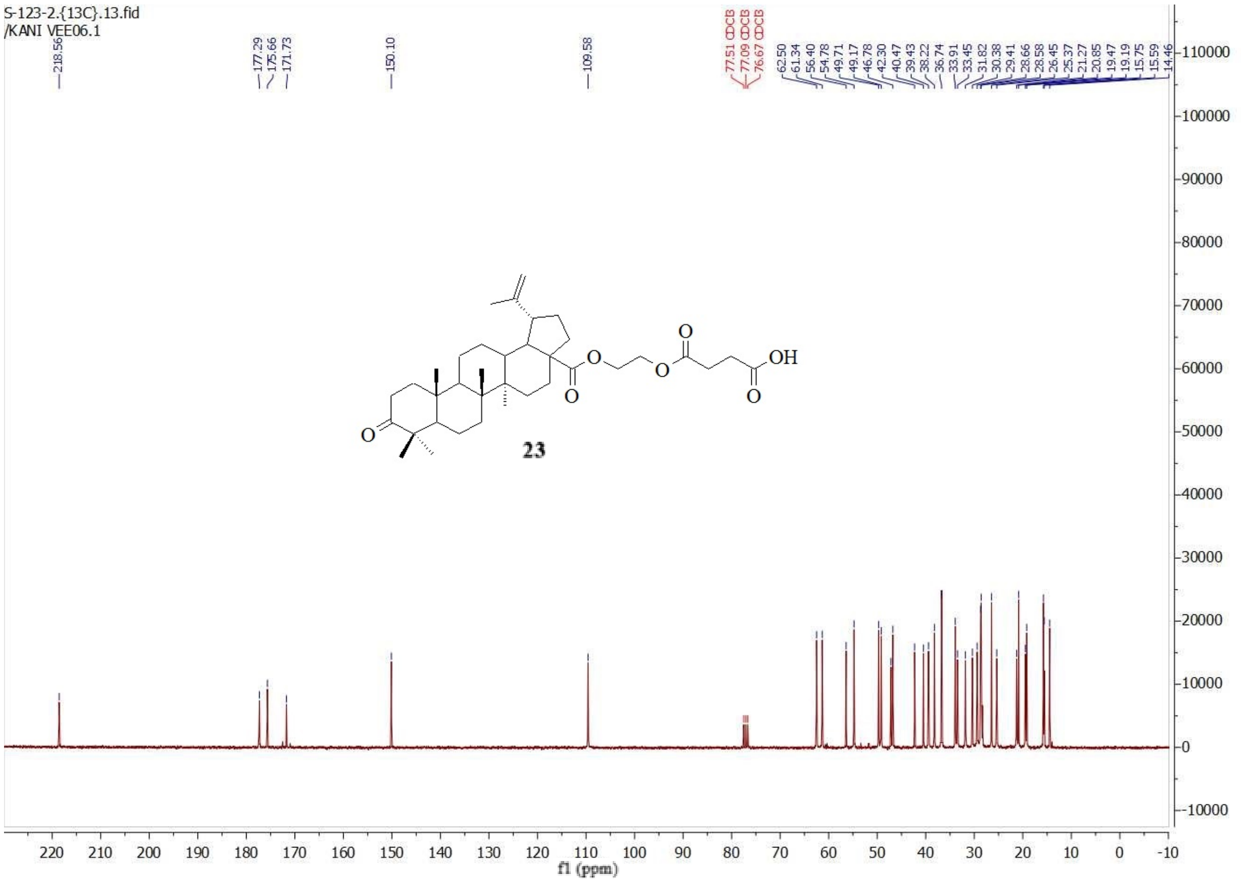

Figure S52 ^{13}C NMR spectra of compound **23.**

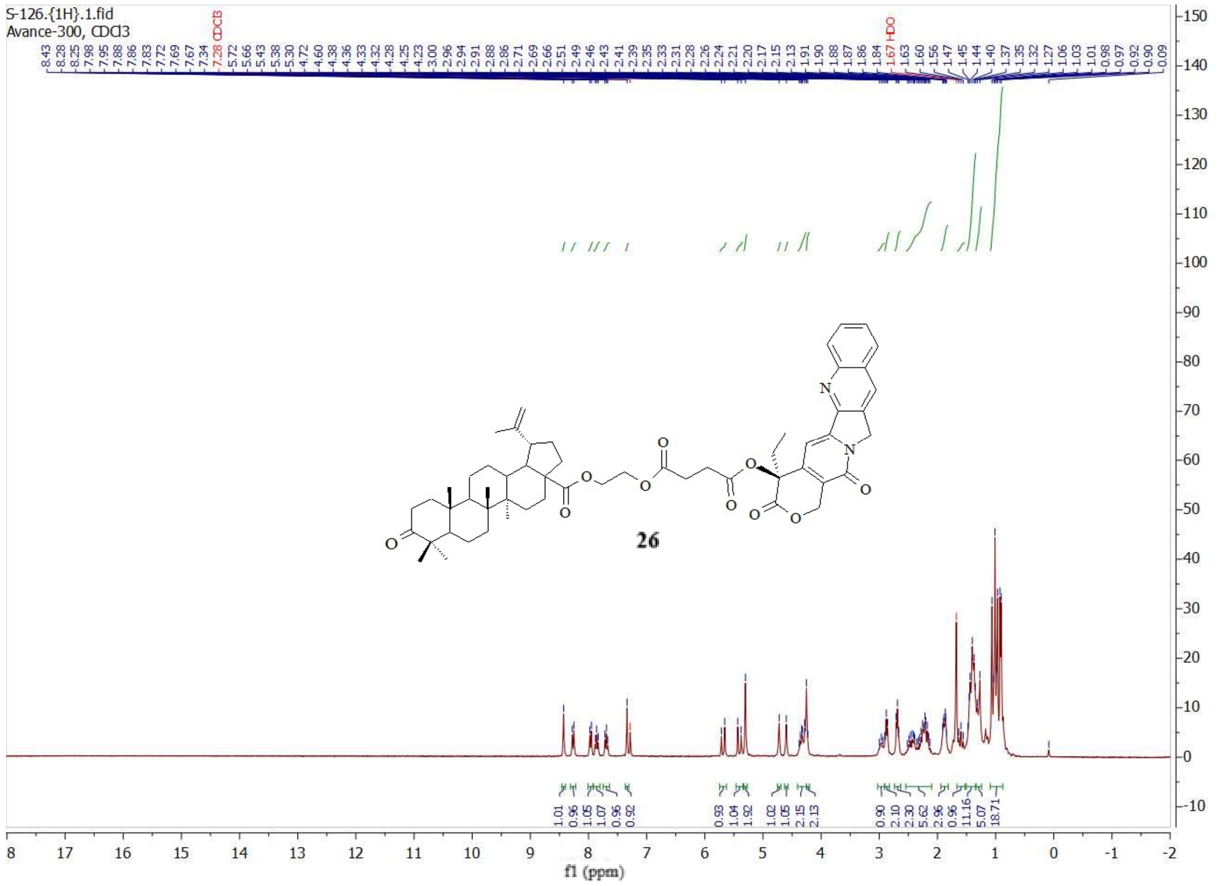

Figure S53 ^{1}H NMR spectra of compound **26.**

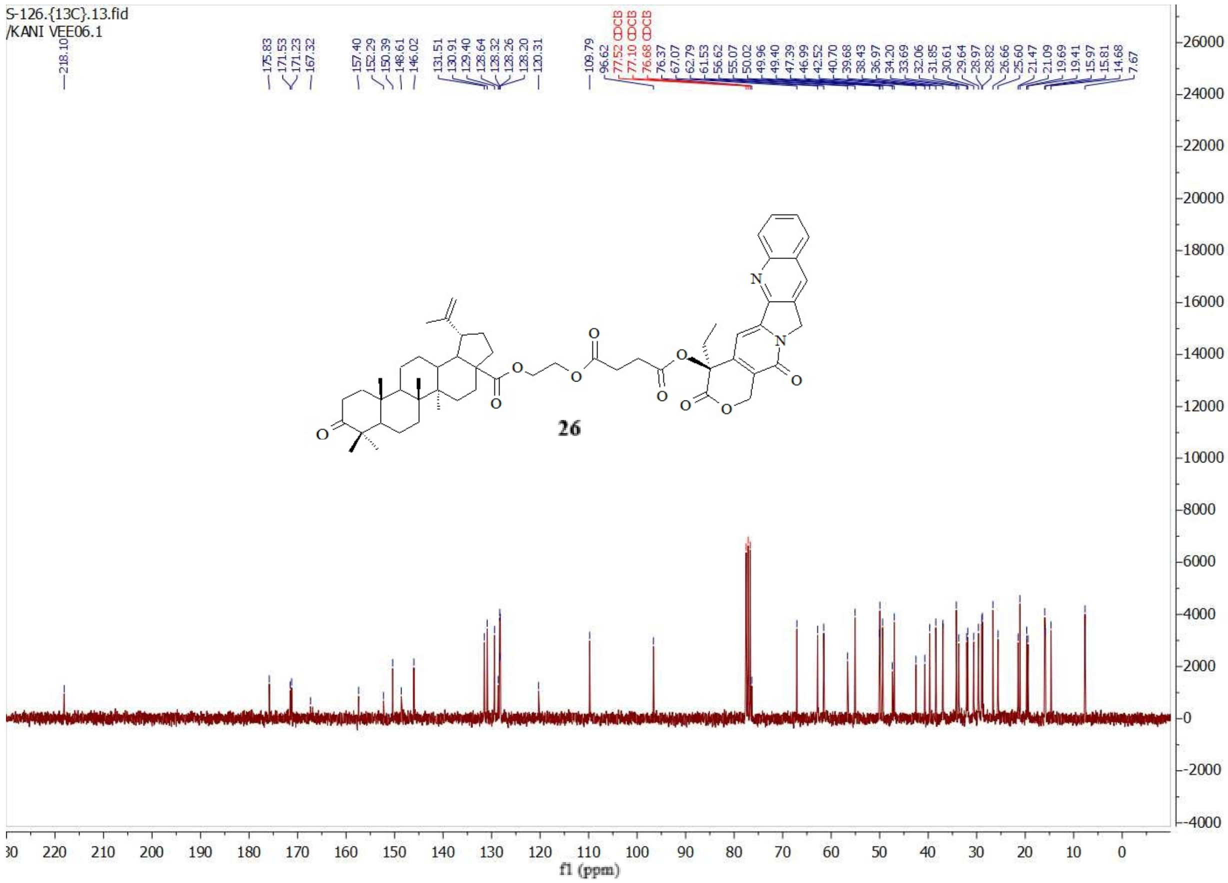

Figure S54 ^{13}C NMR spectra of compound **26.**

Display Report

Analysis Info

Analysis Name	D:\Data\Kolotyrkina\2025\Chobanov\0603029.d	Acquisition Date	03.06.2025 15:49:41
Method	tune_low.m	Operator	BDAL@DE
Sample Name	/ABCD S-126	Instrument / Ser#	micrOTOF 10248
Comment	C56H68N2O10 clb added CH3OH		

Acquisition Parameter

Source Type	ESI	Ion Polarity	Positive	Set Nebulizer	0.4 Bar
Focus	Not active			Set Dry Heater	180 °C
Scan Begin	50 m/z	Set Capillary	4500 V	Set Dry Gas	4.0 l/min
Scan End	3000 m/z	Set End Plate Offset	-500 V	Set Divert Valve	Waste

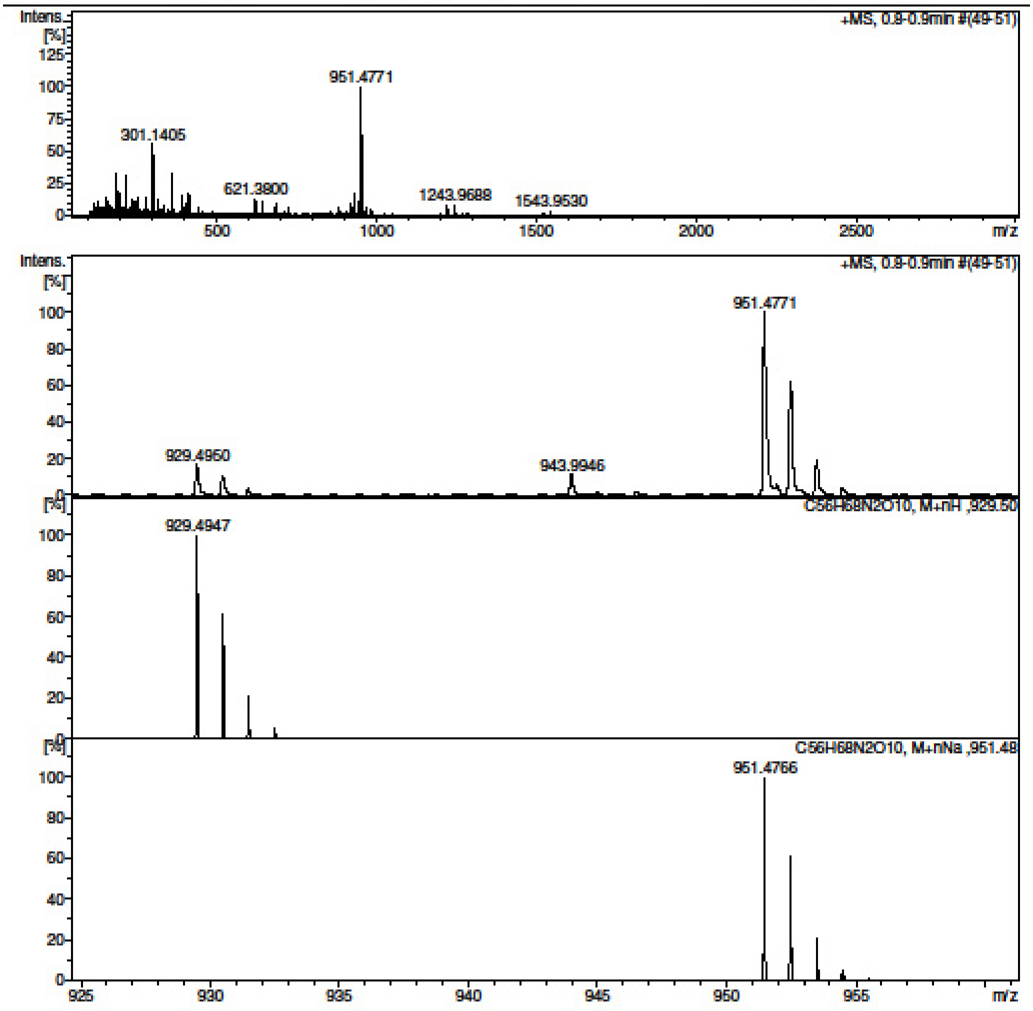

Bruker Compass DataAnalysis 4.0 printed: 03.06.2025 15:52:10 Page 1 of 1

Figure S55 HRMS/MS spectra of compound **26.**

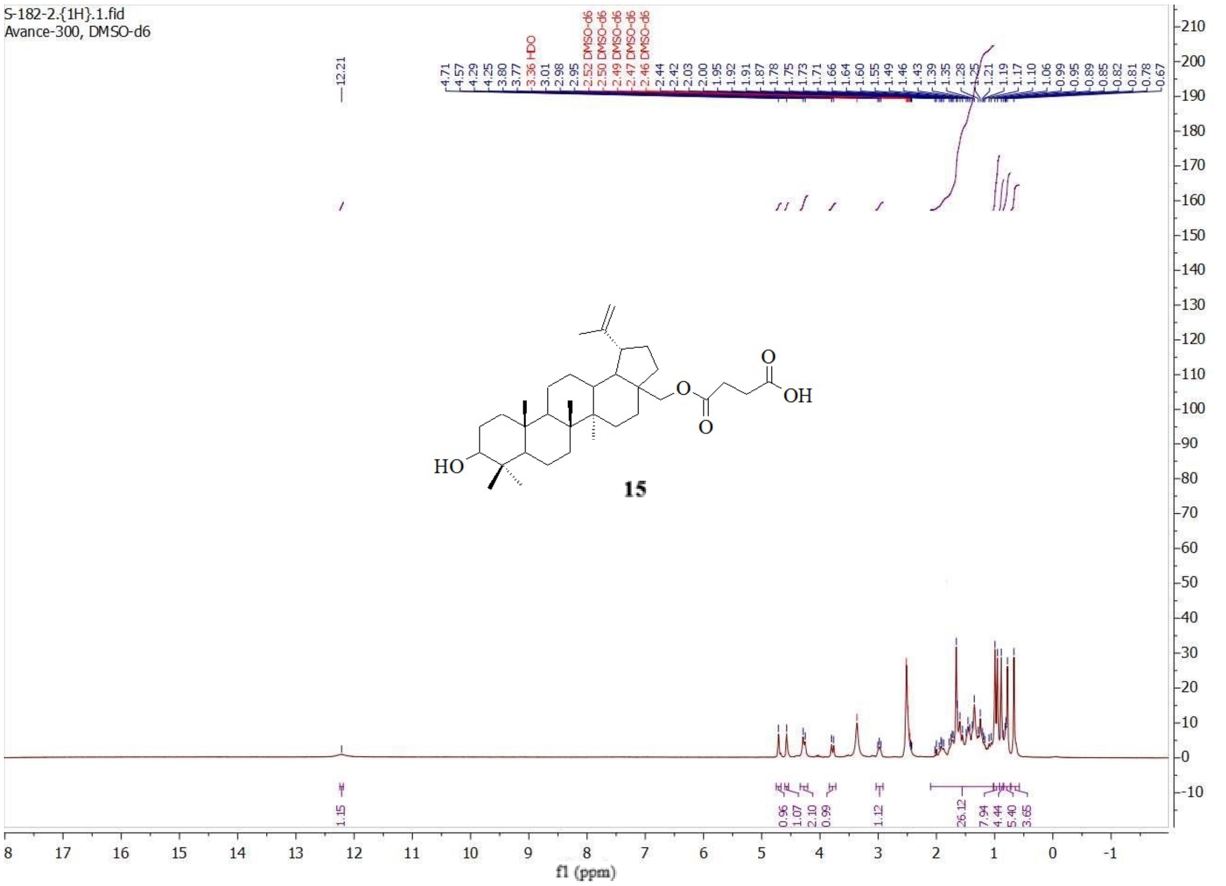

Figure S56 ^{1}H NMR spectra of compound **15.**

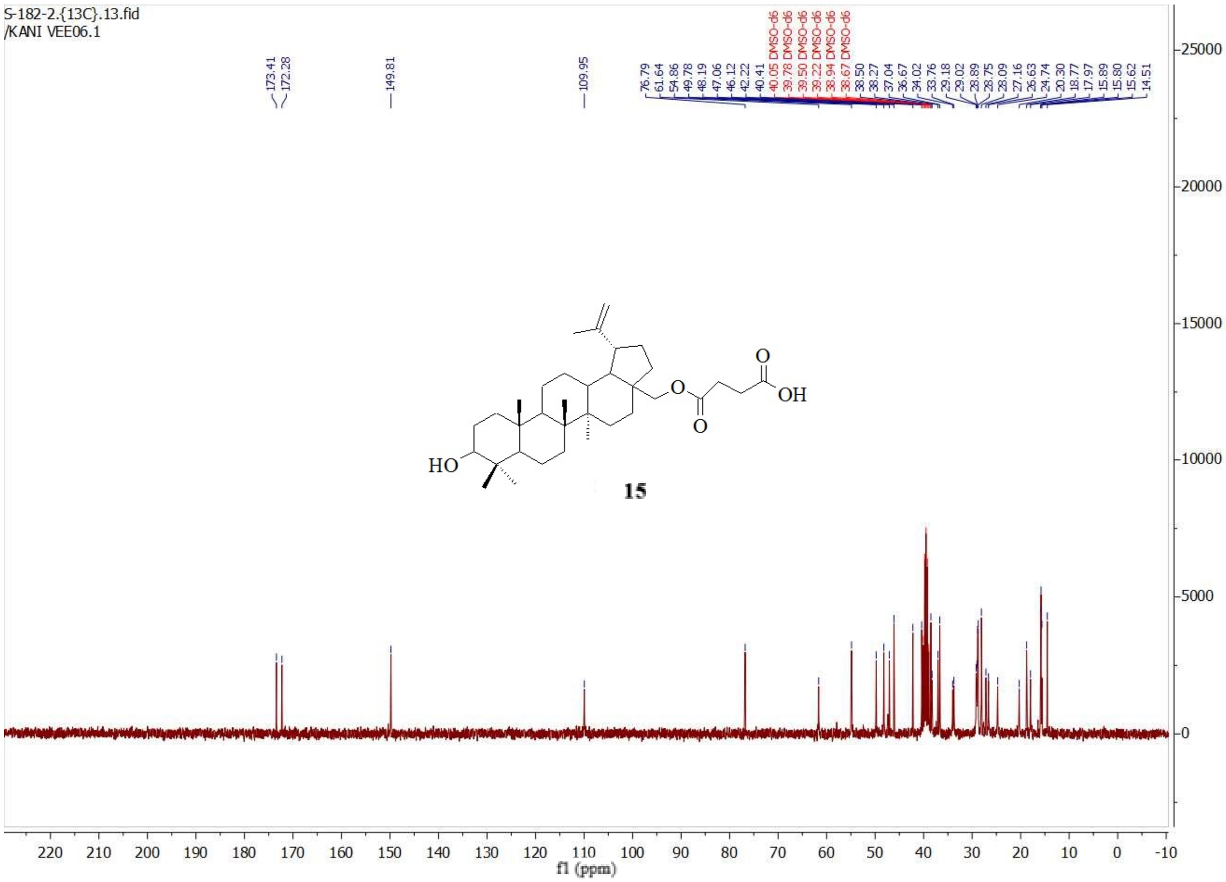

Figure S57 ^{13}C NMR spectra of compound **15.**

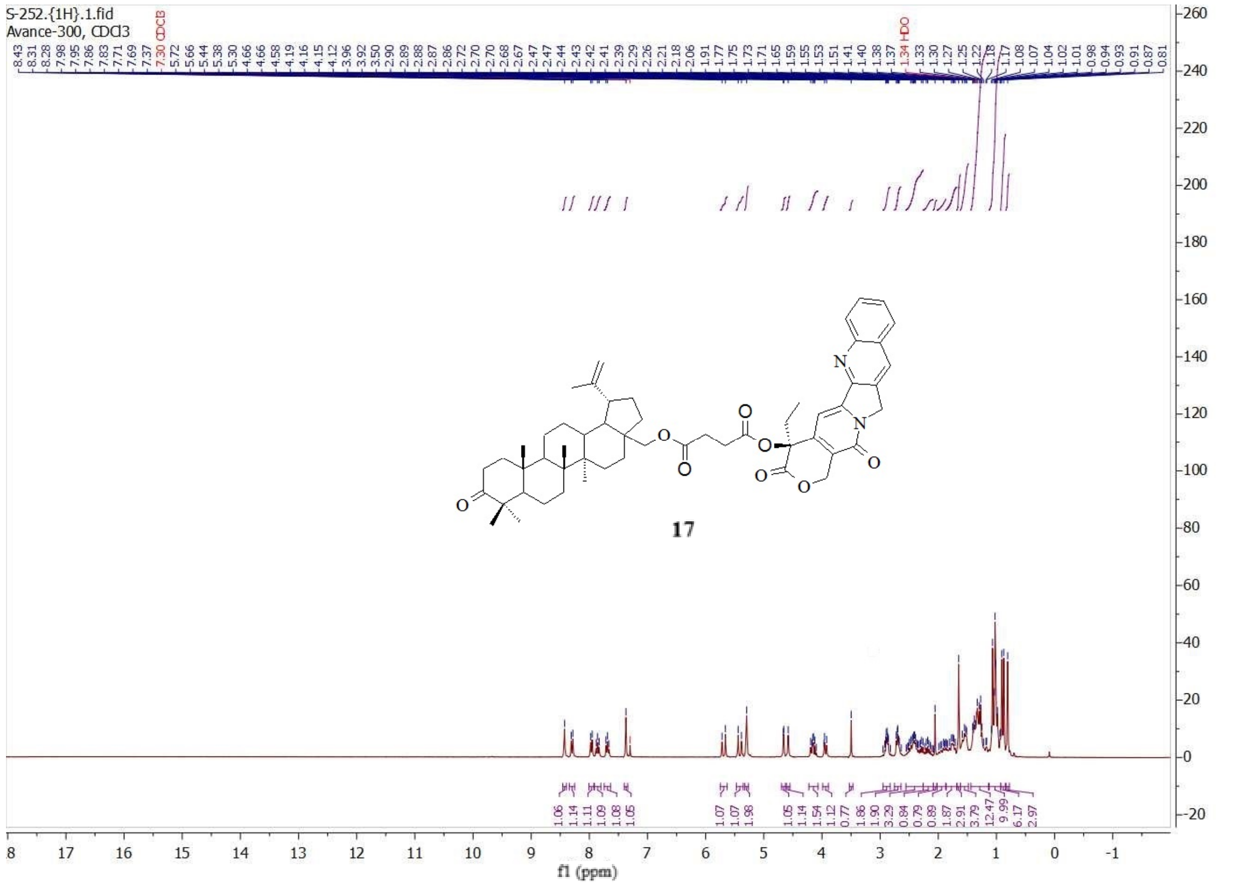

Figure S58 ^{1}H NMR spectra of compound **17.**

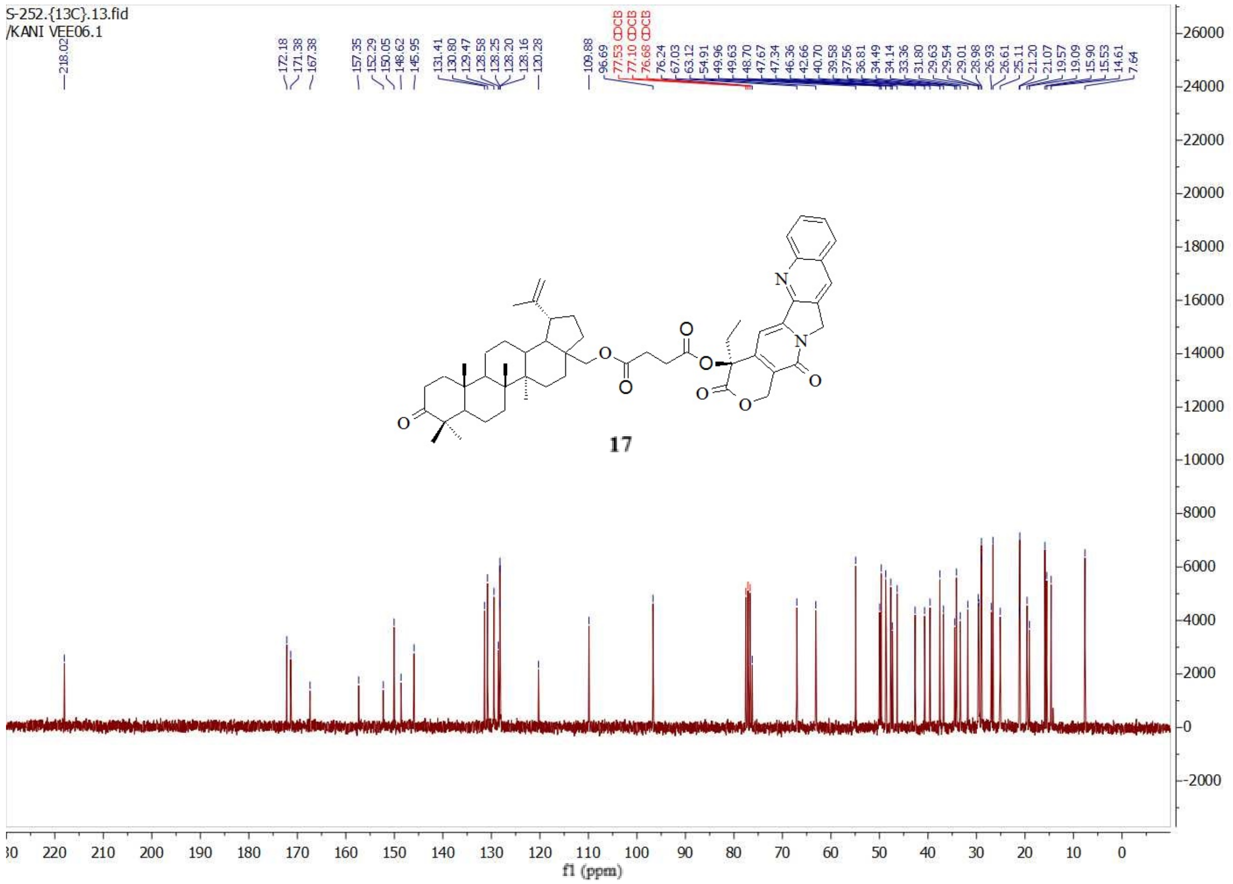

Figure S59 ^{13}C NMR spectra of compound **17.**

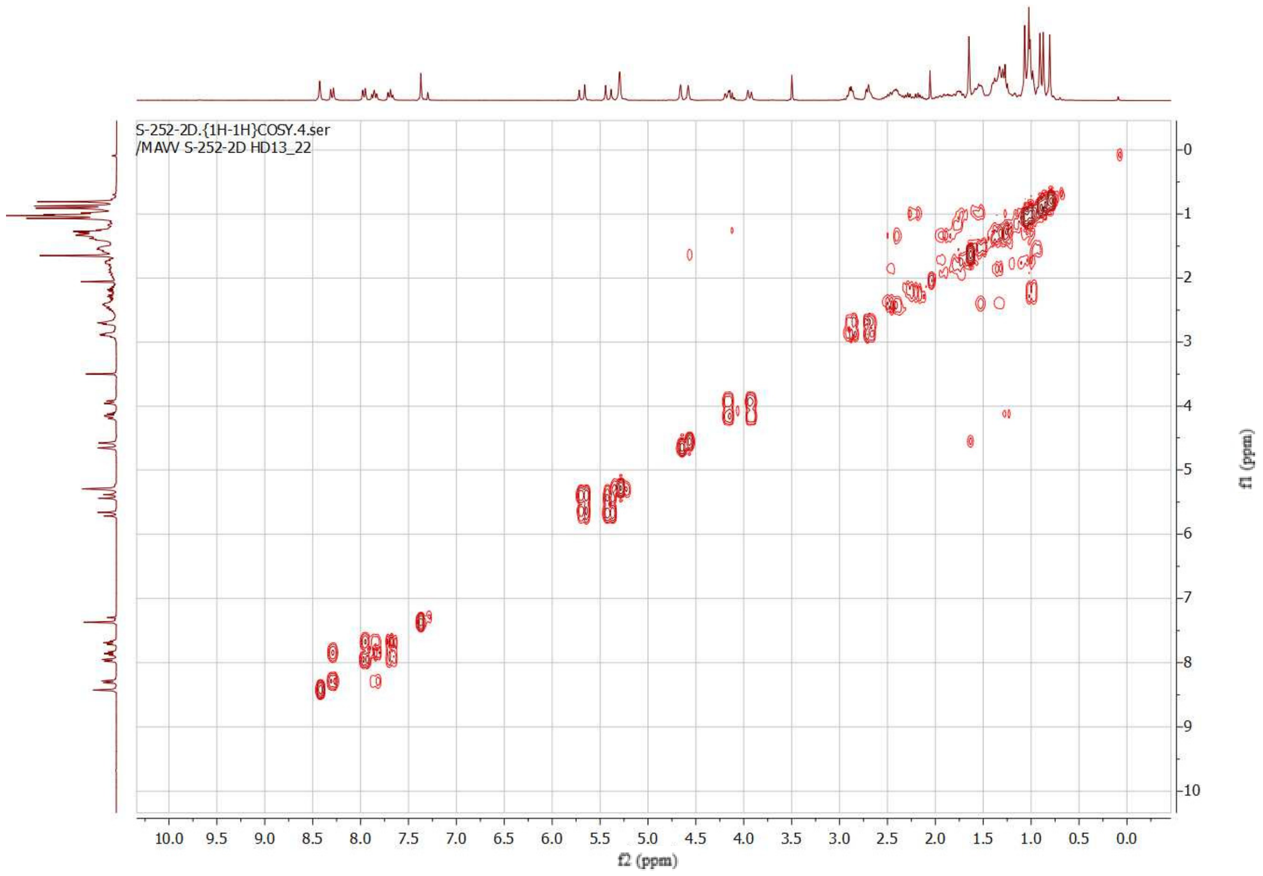

Figure S60 2D NMR (1H-1H) COSY spectra of compound **17.**

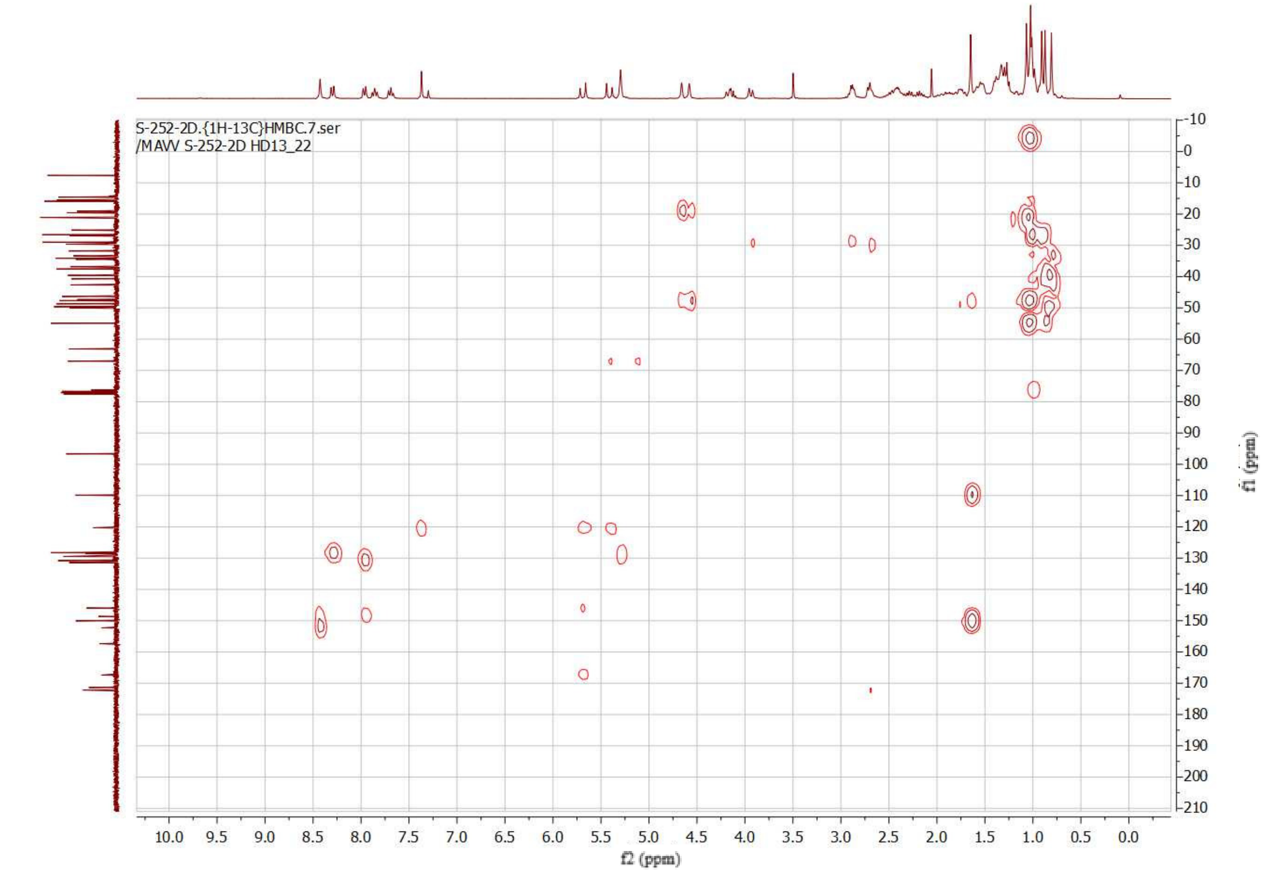

Figure S61 2D NMR (1H-13C) HMBC spectra of compound **17.**

Display Report

Analysis Info

Analysis Name	D:\Data\Kolotyrkina\2025\Chobanov\0603016.d	Acquisition Date	03.06.2025 12:26:30
Method	tune_low.m	Operator	BDAL@DE
Sample Name	/ABCD S-252	Instrument / Ser#	micrOTOF 10248
Comment	C54H66N2O8 clb added CH3OH		

Acquisition Parameter

Source Type	ESI	Ion Polarity	Positive	Set Nebulizer	0.4 Bar
Focus	Not active			Set Dry Heater	180 °C
Scan Begin	50 m/z	Set Capillary	4500 V	Set Dry Gas	4.0 l/min
Scan End	3000 m/z	Set End Plate Offset	-500 V	Set Divert Valve	Waste

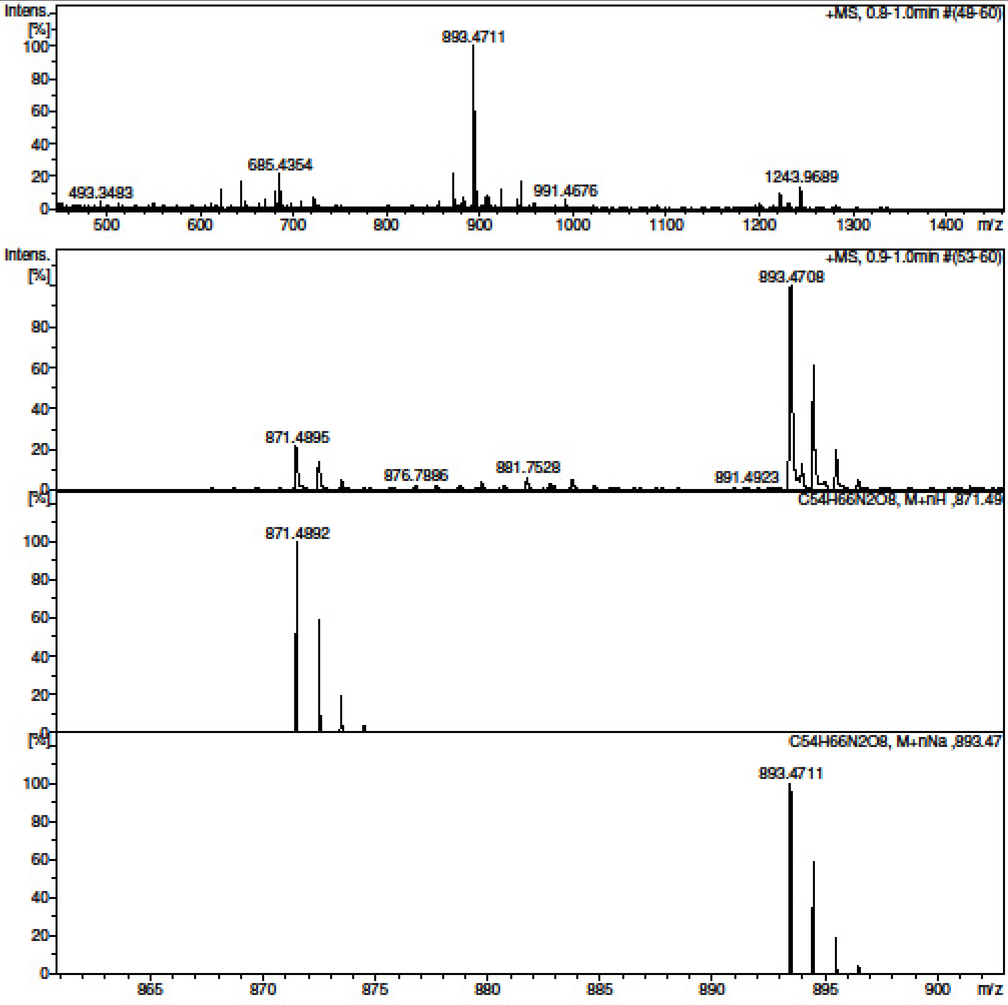

Bruker Compass DataAnalysis 4.0 printed: 03.06.2025 12:30:17 Page 1 of 1

Figure S62 HRMS/MS spectra of compound **17.**

Table S1. Cytotoxicity of synthesized hybrid molecules

Compound cipher	Cipher in article	Molecular weight (Mr. г/моль)	Jurkat	IC fibf/Jurk	HCT 116	IC fibr/HCT116	A549	IC fibr/A549	HL60	IC fibr/HL60	K562	IC fibr/K562	HeLa	IC fibr/HeLa	HEK293	IC fibr/HEK293	Fibroblasts
**Str**		466.541	0.214±0.01	12,00	1.514±0.08	1,60	2.94±0.34	0,80	1.68±0.36	1,50	1.92±0.27	1,30	2.04±0.18	1,30	2.03±0.19	2,03	2.57±0.67
**CPT**	CPT	348.358	0.574±0.02	1,60	1.01±0.048	0,90	1.27±0.56	0,70	1.16±0.11	0,60	1.46±0.68	0,60	0.83±0.26	1,10	0.92±0.06	0,90	0.93±0.06
**S-48**	**8**	901.15	0.443±0.04	1,20	0.523±0.04	1,00	0.57±0.01	0,90	0.47±0.05	1,20	0.57±0.06	0,90	0.67±0.02	0,80	0.48±0.04	0,50	0.56±0.069
**S-99**	**9**	901.15	0.443±0.03	0,70	0.376±0.04	0,80	0.394±0.03	0,80	0.315±0.05	1,00	0.45±0.01	0,70	0.412±0.06	0,80	0.524±0.02	0,50	0.324±0.04
**S-125-2**	**10**	901,49	0.617±0.05	1,90	0.855±0.03	1,30	0.945±0.09	1,20	0.722±0.03	1,60	0.534±0.06	2,20	0.896±0.07	1,30	1.003±0.05	1,10	1.193±0.03
**S-253-2**	**11**	915,47	0.308±0.03	2,50	0.344±0.03	2,30	0.563±0.09	1,40	0.541±0.03	1,40	0.502±0.06	1,50	0.489±0.07	1,60	0.701±0.05	1,10	0.793±0.03
**S-95**	**12**	819.10	0.366±0.05	2,40	0.307±0.06	2,90	0.260±0.05	3,50	0.130±0.05	7,00	0.315±0.06	2,90	0.170±0.01	5,30	0.580±0.06	0,60	0.910±0.05
**S-90-1**	**13**	817.08	0.489±0.06	1,50	0.645±0.01	1,10	0.371±0.06	2,00	0.496±0.03	1,50	0.387±0.07	1,90	0.690±0.04	1,10	0.791±0.02	0,70	0.764±0.07
**S-51**	**14**	819.05	0.488±0.06	1,70	0.393±0.02	2,10	0.683±0.07	1,20	0.578±0.01	1,40	0.616±0.04	1,30	0.573±0.01	1,40	0.615±0.03	0,60	0.833±0.01
**S-252**	**17**	871,49	0.418±0.08	1,80	0.439±0.03	1,70	0.522±0.09	1,40	0.589±0.06	1,20	0.634±0.03	1,10	0.444±0.03	1,60	0.693±0.05	1,00	0.753±0.04
**S-114**	**24**	973,52	0.311±0.11	3,50	0.343±0.07	3,10	0.698±0.11	1,50	0.868±0.03	1,20	0.466±0.06	2,30	0.398±0.01	2,70	0.987±0.01	1,10	1.093±0.02
**S-115**	**25**	973,52	0.293±0.09	3,30	0.401±0.04	2,40	0.517±0.09	1,90	0.654±0.08	1,50	0.555±0.02	1,70	0.423±0.05	2,30	0.887±0.02	1,10	0.993±0.03
**S-126**	**26**	929,49	0.275±0.03	3,10	0.212±0.02	4,10	0.287±0.01	3,00	0.356±0.05	2,40	0.289±0.01	3,00	0.345±0.04	2,50	0.635±0.01	1,30	0.875±0.07

Table S2. Physico-chemical properties and ADME prediction

ADME characteristic	Compounds											
	**CPT**	**8**	**9**	**10**	**11**	**12**	**13**	**`14**	**17**	**24**	**25**	**26**
**TPSA (topological polar surface area)**	81.42	140.09	140.09	140.09	157.16	113.79	113.79	140.09	130.86	166.39	166.39	157.16
**Rat Oral Acute Toxicity LD50 upon administration (LD50 of acute toxicity) –log**	0.729	0.666	0.738	0.739	0.584	0.277	0.45	0.372	0.476	0.377	0.502	0.327
**FDA Maximum (Recommended) Daily Dose (FDAMDD) in human**	0.99	0.999	1.0	1.0	1.0	1.0	1.0	1.0	0.999	0.999	0.999	0.998
**mol/kg (mg/kg),**	348.11	900.49	900.49	898.48	914.47	818.49	816.47	818.41	870.48	972.51	972.51	928.49
**Solubility LogS, log mol/L (mg/mL)**	-3.392	-6.855	-6.339	-7.585	-5.800	-6.902	-6.836	-6.357	-6.919	-7.254	-7.681	-6.978

**Distribution coefficient D LogD7,4**	1.799	4.455	4.227	4.530	3.458	5.864	5.662	4.687	4.477	4.568	4.685	4.452
**Distribution coefficient P LogP**	1.442	5.885	5.370	6.300	3.781	9.233	8.970	6.809	5.409	6.662	6.788	5.749
**PAPP (Caco-2 permeability), cm/s**	-5.091	-5.388	-5.429	-5.358	-5.406	-5.176	-5.172	-5.381	-5.313	-5.428	-5.401	-5.143
**Ability to inhibit glycoprotein-P**	-	+++	+++	+++	+++	+++	+++	+++	+++	+++	+++	+++
**Ability to be glycopro- tein-P substrate**	++	--	---	---	---	----	----	---	---	---	---	---
**Human intestinal ab- sorption**	---	-	---	---	---	---	----	---	---	---	---	---
**30% Bioavailability (F30%)**	---	+++	+++	+++	+++	+++	+++	+++	+++	+++	+++	+++
**Plasma protein binding, %**	98.9 %	98.4 %	98.2 %	98.6 %	98.1 %	99.0 %	98.7 %	97.7 %	98.8 %	97.0 %	97.0 %	98.1 %

**Volume of distribution, L/kg**	-0.366	0.242	0.389	0.304	0.073	0.478	0.43	0.02	0.607	0.096	0.255	0.514
**Blood–brain barrier permeability**	-	-	---	---	---	---	---	---	+	---	---	--
**Halflife (T½), h**	1.497	0.519	0.639	0.594	0.429	0.387	0.347	0.37	0.504	0.508	0.561	0.523
**Clearance, mL/min*kg**	6.248	5.61	4.644	4.607	6.182	5.801	5.244	5.116	5.771	4.287	3.91	5.181
**Cardiotoxicity (hERG blockade)**	0.091	0.147	0.207	0.231	0.210	0.34	0.308	0.185	0.272	0.068	0.093	0.164
**Human hepatotoxicity**	0.974	0.978	0.984	0.98	0.995	0.991	0.98	0.789	0.973	0.928	0.946	0.957
**Skin sensitization**	0.941	0.887	0.901	0.974	0.954	0.974	0.944	0.87	0.96	0.954	0.922	0.863

A comprehensive evaluation of quantitative and qualitative parameters was conducted during the study. The evaluation of fundamental physicochemical properties encompassed the following parameters: water solubility (LogS), lipophilicity (LogD7.4), distribution between the aqueous phase (phosphate buffer at pH7.4) and an immiscible organic solvent (typically octanol), and lipophilicity distribution coefficient (LogP) at equilibrium concentrations. The assessment of intestinal absorption capacity was determined by predicted intestinal permeability (PAPP) using the Caco-2 cell line model. A qualitative evaluation was conducted to assess the capacity of compounds to function as either inhibitors (Pgp-Inhibitor) or substrates (Pgp-Substrate) of glycoprotein-P, and to determine their impact on human intestinal absorption (HIA). A classification model based on physicochemical descriptors was used to calculate bioavailability greater than 30% for oral administration (F30%). The distribution of hybrid molecules was evaluated by three parameters: binding to plasma proteins (PPB, %), volume of distribution (VD), and a categorical index of the ability to penetrate the blood-brain barrier (BBB permeability). The metabolism was determined by the potential for interaction with five cytochromes: CYP1A2, CYP3A4, CYP2C19, CYP2C9, and CYP2D6. The assessment of elimination was conducted by calculating the clearance and elimination half-life. A comprehensive evaluation of toxicity was conducted, encompassing six qualitative indicators. Additionally, the acute toxicity dose when administered orally (LD50) was calculated. The assessment of cardiotoxicity was determined by the probability of hERG gene blockade, which has the potential to result in arrhythmias and QT interval prolongation. Hepatotoxicity (H-HT) was determined by the toxicity of reactive metabolites, and mutagenicity was determined by the Ames test (AMES). The probability of immune reactions resulting in skin sensitization (SkinSen), drug-induced liver injury (DILI), and the maximum recommended daily dose for humans (FDAMDD) was also assessed. FDAMDD is the estimated upper dose limit beyond which the efficacy of a drug does not increase and/or undesirable side effects begin to outweigh the beneficial effects. Rat oral acute toxicity is a measure of the short-term poisoning potential of a substance when ingested. This potential is commonly expressed as the LD50 (median lethal dose) in rats. The LD50 is the amount of a substance that is expected to cause death in 50% of a test group of rats after a single dose over a set period (e.g., 14 days). This testing is employed for the identification and regulation of hazards, including chemical classification and labeling. However, alternative computational and in vitro methods are being developed. The following link contains additional information on the subject: https://www.molecular-modelling.ch/swiss-drug-design.html. The machine-learning algorithm employed by the web server is designed to assess the degree of similarity between the compound under study and existing drugs, with a focus on ADMET and related properties. The program utilizes heuristic approaches to facilitate the identification of optimal bioavailability of the molecule when administered orally. The fundamental principle of Lipinski's rule is predicated on the premise that the molecular weight of a given compound should not exceed 500, that the content of nitrogen or oxygen atoms should not exceed 10, and that the number of hydroxy or amine groups serving as hydrogen bond donors should not exceed 5. Moreover, the requisite solubility in water is defined by a logP value that should not exceed 5. The Ghosh filter constitutes a modification of the original formula and is based on the following parameters: LogP: $-5.6 < LogP < -0.4$; molecular weight: $160 < Mw < 480$; molar refraction: $40 < Mr < 130$; number of atoms: $20 < n$ atoms < 70. However, Lipinski's rule was only met in 75% of compounds due to the issue of low solubility. Oprey's rule establishes a quantitative framework for evaluating the propensity of chemical compounds to serve as drug candidates. The rule stipulates that the probability of a compound meeting the

criteria as a drug candidate is elevated if the number of rings is limited to three, the presence of rigid bonds is constrained to a maximum of 18, and the number of rotational bonds is restricted to six. As demonstrated in Figure 1, Oprey's rule was fulfilled for all compounds except camptothecin, which has a low number of rotatable bonds. The experimental results obtained from the synthesis of hybrid molecules conformed to the established principles of Weber's and Varm's rules.
